# Single-cell spatial pharmacobiology identifies conserved stromal barriers to therapeutic antibody delivery in human solid tumors

**DOI:** 10.1038/s41587-026-03152-x

**Published:** 2026-06-03

**Authors:** Guolan Lu, John W. Hickey, Maximilian Haist, Xulei Qin, Emily Zhao, Abdullah Naveed, Erna Forgo, Marc-A. Baertsch, Lucas Mani, Xavier Rovira-Clavé, Andrey Finegersh, Yury Goltsev, Chiara Caraccio, Nynke S. van den Berg, Marisa Hom, Deana R. Colburg, Brock A. Martin, Christina S. Kong, Natalie S. Lui, George A. Fisher, A. Dimitrios Colevas, Robert B. West, Greg M. Thurber, George A. Poultsides, Garry P. Nolan, Eben L. Rosenthal

**Affiliations:** 1 Department of Urology, Stanford University School of Medicine, Stanford, CA, USA.; 2 Department of Otolaryngology, Stanford University School of Medicine, Stanford, CA, USA.; 3 Department of Microbiology and Immunology, Stanford University School of Medicine, Stanford, CA, USA.; 4 Department of Pathology, Stanford University School of Medicine, Stanford, CA, USA.; 5 Department of Biomedical Engineering, Duke University, Durham, NC, USA.; 6 Department of Dermatology, University Medical Center of the Johannes Gutenberg University Mainz, Mainz, Germany.; 7 Department of Surgery, Stanford University, Stanford, CA, USA.; 8 Department of Otolaryngology, Vanderbilt University Medical Center, Nashville, TN, USA.; 9 Department of Otolaryngology, Tulane University School of Medicine, New Orleans, LA, USA.; 10 Department of Pathology, Robert J. Tomsich Pathology & Laboratory Medicine Institute, Cleveland Clinic, Cleveland, OH, USA.; 11 Department of Hematology, Oncology and Rheumatology, Heidelberg University Hospital, Heidelberg, Germany.; 12 Clinical Cooperation Unit Molecular Hematology/Oncology, German Cancer Research Center, Heidelberg, Germany.; 13 Institute for Bioengineering of Catalonia, Barcelona Institute of Science and Technology, Barcelona, Spain.; 14 Department of Cardiothoracic Surgery, Stanford University School of Medicine, Stanford, CA, USA.; 15 Department of Medicine (Oncology), Stanford University School of Medicine, Stanford, CA, USA.; 16 Department of Chemical Engineering, University of Michigan, Ann Arbor, MI, USA.; 17 Department of Biomedical Engineering, University of Michigan, Ann Arbor, MI, USA.; 18 Rogel Cancer Center, University of Michigan, Ann Arbor, MI, USA.

## Abstract

The development of effective antibody therapeutics has been hampered by a lack of methods to measure drug delivery and activity within tumors at single-cell resolution. Here we introduce single-cell spatial pharmacobiology (SSP), an experimental and analytical framework that integrates in situ imaging of a systemically infused, fluorescently labeled therapeutic antibody with high-plex spatial proteomics to quantify antibody distribution, target engagement and tumor microenvironment (TME) architecture. We applied SSP to tumor tissues from participants with head and neck squamous cell carcinoma and pancreatic ductal adenocarcinoma who received the antibody panitumumab-IRDye800 in phase 1 trials. SSP identified pronounced spatial heterogeneity in single-cell drug delivery and target engagement, shaped by conserved stromal barriers, including periostin-rich extracellular matrix assemblies and fibroblast-activation-protein-positive cancer-associated fibroblast neighborhoods, which were associated with reduced antibody delivery in both tumor types. SSP measures drug–target–TME interactions in human tumors and can support studies of resistance mechanisms, dosing strategies and discovery of spatial biomarkers for precision oncology.

Antibody-based therapeutics have transformed lives across cancer, autoimmunity and inflammatory diseases, offering high target specificity, reduced toxicity and durable responses^1–3^. However, their clinical success remains limited, with only ~10% likelihood of approval from phase 1 clinical trials for all agents and half of that for oncology^4,5^ and response rates in solid tumors hovering around 20% (refs. 6,7). One major reason lies in our limited understanding of how therapeutic antibodies distribute (pharmacokinetics, PK), engage their molecular targets (pharmacodynamics, PD) and are shaped by the tumor microenvironment (TME) in human tumors.

Standard clinical pharmacology tools, such as plasma-based assays^8^ and positron emission tomography (PET) and single-photon emission computed tomography (SPECT) imaging of radiolabeled antibodies^9^, provide systemic or organ-level drug distribution but lack the spatial resolution to interrogate intratumoral drug distribution or cell-specific drug–target interactions^10^. Preclinical models and computational simulations offer mechanistic insights but fail to capture the structural and cellular complexity of human tumors, limiting their translational predictive value^11–17^. Consequently, it remains unclear whether therapeutic failure in solid tumors is primarily driven by insufficient drug penetration, suboptimal target engagement or TME resistance mechanisms. This underscores a critical unmet need for spatially resolved methods that can simultaneously measure drug delivery, target engagement and TME architecture at cellular resolution in intact human tumors.

To address this gap, we developed single-cell spatial pharmacobiology (SSP)—an experimental and analytical framework that decodes drug–target–TME interactions in situ by integrating high-resolution imaging of systemically infused therapeutic antibody with highly multiplexed spatial proteomics. As an initial demonstration, we used panitumumab-IRDye800 (pan800), a near-infrared dye-labeled EGFR-targeting antibody, as a surrogate to visualize antibody distribution. To map drug distribution within the TME, we constructed a comprehensive DNA-barcoded antibody panel to map the spatial composition and organization of major classes of extracellular matrix (ECM) constituents (collagens and glycoproteins), cancer-associated fibroblasts (CAFs), tumor cells, immune cells and vasculature. This integrative approach enabled us to explore previously unanswerable questions in drug pharmacology in human tumors, including how the drug is distributed inside the tumor, which cell types it interacts with, the degree of engagement with its molecular target and how the TME influences drug delivery and activity.

We applied SSP to tumor tissues from participants with head and neck squamous cell carcinoma (HNSCC) and pancreatic ductal adenocarcinoma (PDAC), who received intravenous infusion of pan800 in phase 1 clinical trials^18,19^. While antibody therapeutics including EGFR inhibitors show modest efficacy in HNSCC, they have largely failed in PDAC. We reasoned that investigating drug–target–TME interactions in these two solid cancers could reveal common mechanisms restricting therapeutic efficacy. Using SSP, we observed pronounced spatial heterogeneity in single-cell drug delivery and target engagement, shaped by local ECM assemblies and CAF-rich niches. Specifically, peritumoral fibroblast activation protein (FAP)^+^ CAFs and periostin-rich ECM neighborhoods were associated with impaired antibody penetration, which were conserved across both tumor types, implicating stromal barriers as a potential drug resistance mechanism.

SSP provides a framework for dissecting how the spatial organization of cells and ECM influence drug pharmacology at cellular resolution within intact human tumors. This approach is generalizable to a wide range of antibody therapeutics, including immune checkpoint inhibitors and antibody–drug conjugates. When implemented in early-stage clinical trials, SSP has the potential to advance the development of next-generation cancer therapeutics by uncovering mechanisms of response and resistance, guiding individualized dosing and identifying spatial biomarkers for participant stratification.

## Results

### SSP enables quantification of therapeutic antibody delivery and activity at cellular resolution in the intact human TME

**Fig. 1 |:**
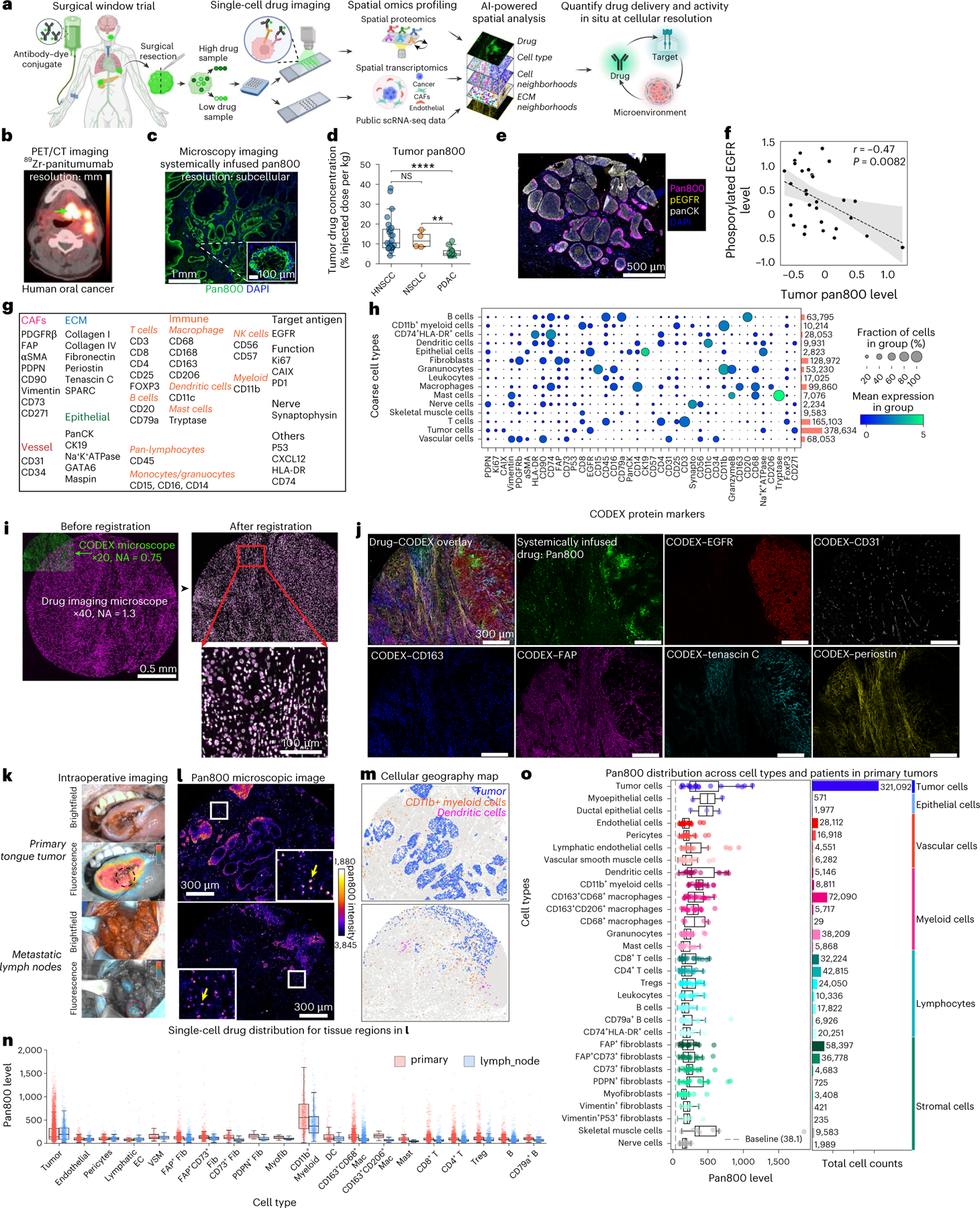
SSP quantifies therapeutic antibody delivery and activity in native tissue at single-cell resolution. **a**, Overview of the SSP methodology. **b**, Representative PET/CT imaging of ^89^Zr-panitumumab uptake in a participant with oral cancer. **c**, High-resolution microscopic imaging of systemically infused pan800 distribution in a representative participant with HNSCC. **d**, Average pan800 concentrations measured from bulk tumor tissue homogenates across tumor types. Each dot represents one participant (HNSCC, *n* = 25; NSCLC, *n* = 4; PDAC, *n* = 14). Box plots indicate the median and interquartile range. Statistical comparisons were performed using a two-tailed Mann–Whitney *U*-test: HNSCC versus NSCLC, *P* = 1.00; HNSCC versus PDAC, *P* = 4.42 × 10^−5^; NSCLC versus PDAC, *P* = 7.84 × 10^−3^. *P < 0.05, **P < 0.01, ***P < 0.001 and ****P < 0.0001. **e**, Representative image overlay for pEGFR and pan800 in the HNSCC tissue. **f**, Scatter plot depicting the inverse correlation between *z*-normalized pEGFR expression and log-transformed pan800 intensity in tumor cells from HNSCC. Each point represents one tissue region (TMA core; *n* = 30). The Pearson correlation coefficient (*r*) and *P* value (two-sided) are shown, alongside a linear regression plot with the 95% confidence interval indicated by shaded area. **g**, Panel of DNA-barcoded antibodies used for CODEX multiplexed imaging to detect CAFs, vessels, ECM, immune cells, nerves, tumor cells and target antigen. **h**, Dot plot of protein marker expression across major cell types identified from segmented single cells in surgical specimens from *n* = 18 participants with HNSCC. Dot size represents the fraction of cells expressing the marker and color indicates the mean normalized expression. **i**, Demonstration of coregistration of systemically infused pan800 microscopic imaging with CODEX multiplexed imaging for HNSCC tissues. **j**, Representative images of spatial colocalization of pan800 with EGFR, CD31, CD163, FAP, tenascin C and periostin. Images are representative of multiple independent tissue regions across participants processed using the same registration pipeline with consistent results. **k**–**n**, Intraoperative wide-field imaging of pan800 (**k**), high-resolution microscopic pan800 imaging (**l**), CODEX-derived cell phenotypes maps (**m**) and single-cell drug distribution (**n**) from a primary tongue tumor and its matched metastatic lymph node in a representative participant. In **n**, each dot represents one cell; these cells are from one primary tumor region and one metastatic lymph node region matching (**l**). **o**, Comparison of single-cell pan800 fluorescence intensity across major cell types in primary HNSCC tumors (*n* = 18 participants). Each dot represents one participant, with values calculated as the mean pan800 intensity per cell type averaged across tumor regions. A vertical dashed line indicates baseline near-infrared imaging signal measured from noninfused control tissues. Differences across cell types reflect relative pan800 enrichment above background. Box plots in **n**,**o** show the median (center line), interquartile range (box bounds, 25th–75th percentiles) and whiskers extending to the minimum and maximum values within 1.5× the interquartile range from the lower and upper quartiles, respectively. Data points beyond this range are not shown. Panel **a** created in BioRender; Lu, G. https://biorender.com/mq08om0 (2026).

The SSP method involves optical labeling of a therapeutic antibody, intravenous infusion of the labeled drug into individuals before standard-of-care surgical resection and multimodal imaging of the drug, drug target and TME in excised tissues (Fig. 1a). From 2018 to 2021, commercially available panitumumab was fluorescently labeled under Good Manufacturing Practices conditions and systemically administered in 50 participants with HNSCC, PDAC or non-small cell lung cancer (NSCLC). Participants were enrolled in three separate, phase 1 clinical trials at Stanford Cancer Institute investigating the safety and PK of the EGFR inhibitor pan800 as an optical imaging agent^19,20^. In these single-arm, open-label clinical trials, participants were administered pan800 at subtherapeutic doses for surgical guidance. This method differs notably from PET imaging of radiolabeled antibodies (for example, ^89^Zr-panitumumab), which is typically used to assess the whole-body antibody distribution. While PET imaging has insufficient spatial resolution to determine whether the drug binds its molecular targets (Fig. 1b), optical imaging of fluorescently labeled therapeutic antibody enables visualization of drug distribution at cellular resolution in clinical tumors. This allows for direct, in situ quantification of drug delivery and target engagement, offering a unique and complementary view to conventional approaches (Fig. 1c).

By comparing intratumoral drug concentrations at the participant level across HNSCC (*n* = 25), NSCLC (*n* = 4) and PDAC (*n* = 14), we found that mean drug concentrations were significantly lower in PDAC compared to HNSCC (two-tailed Mann–Whitney *U*-test, *P* < 0.0001; Fig. 1d) and NSCLC (two-tailed Mann–Whitney *U*-test, *P* < 0.01). This is consistent with the dense fibrotic stroma of PDAC that may hinder drug penetration. Notably, we observed substantial interparticipant heterogeneity in tumor drug concentrations, particularly in participants with HNSCC, where drug concentrations ranged from 6.4% to 38.1% injected dose per kg. Some HNSCC tumors exhibited low drug concentrations comparable to those observed in PDAC, suggesting that pathophysiological barriers to drug delivery also exist within HNSCC. To assess the molecular effect of EGFR inhibition by panitumumab, we evaluated phosphorylated EGFR (pEGFR) and systemically infused pan800 in tissues from the same participant (Fig. 1e). We found a significant inverse correlation between pan800 delivery and EGFR phosphorylation across representative tumor regions (Pearson *r* = −0.47, *P* = 0.0082, *n* = 30 regions; Fig. 1f), indicating that higher intratumoral drug delivery was associated with greater EGFR pathway inhibition. Because pan800 was administered at subtherapeutic imaging doses, pEGFR is interpreted here as a relative, local marker of EGFR target engagement rather than as evidence of full pathway inhibition at the tumor or participant level. Collectively, these data highlight the importance of investigating drug delivery barriers as a potential mechanism of resistance to antibody therapy.

**Extended Data Fig. 1 |:**
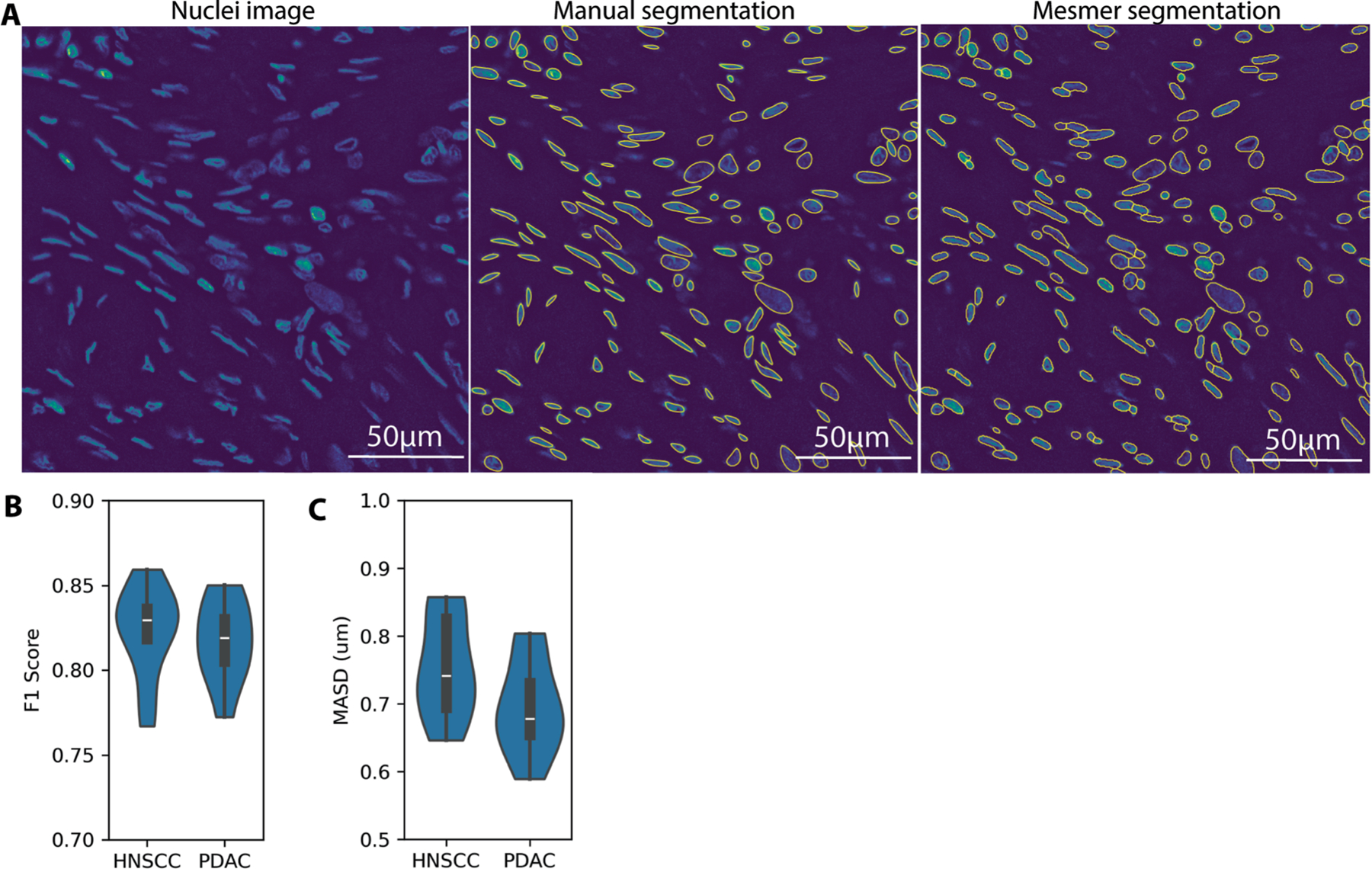
Segmentation benchmarking in dense stromal regions. (**a**) Representative images showing nuclei image, cell boundary overlays from manual segmentation by two independent experts, and from the Mesmer algorithm. (**b**) Violin plots of the F1 score for Mesmer segmentation compared with manual segmentation across 10 dense stromal regions from HNSCC and 10 from PDAC. (**c**) Violin plots of the mean absolute surface distance (MASD; μm) for Mesmer segmentation compared with manual segmentation across 10 dense stromal regions from HNSCC and 10 from PDAC. In (**b-c**), violin plots represent the distribution of the data estimated by kernel density. The embedded boxplots indicate the median (center line), interquartile range (box bounds, 25th–75th percentiles), and whiskers extending to the most extreme data points within 1.5× the interquartile range. The minima and maxima correspond to the lowest and highest data points within the whisker range.

**Extended Data Fig. 2 |:**
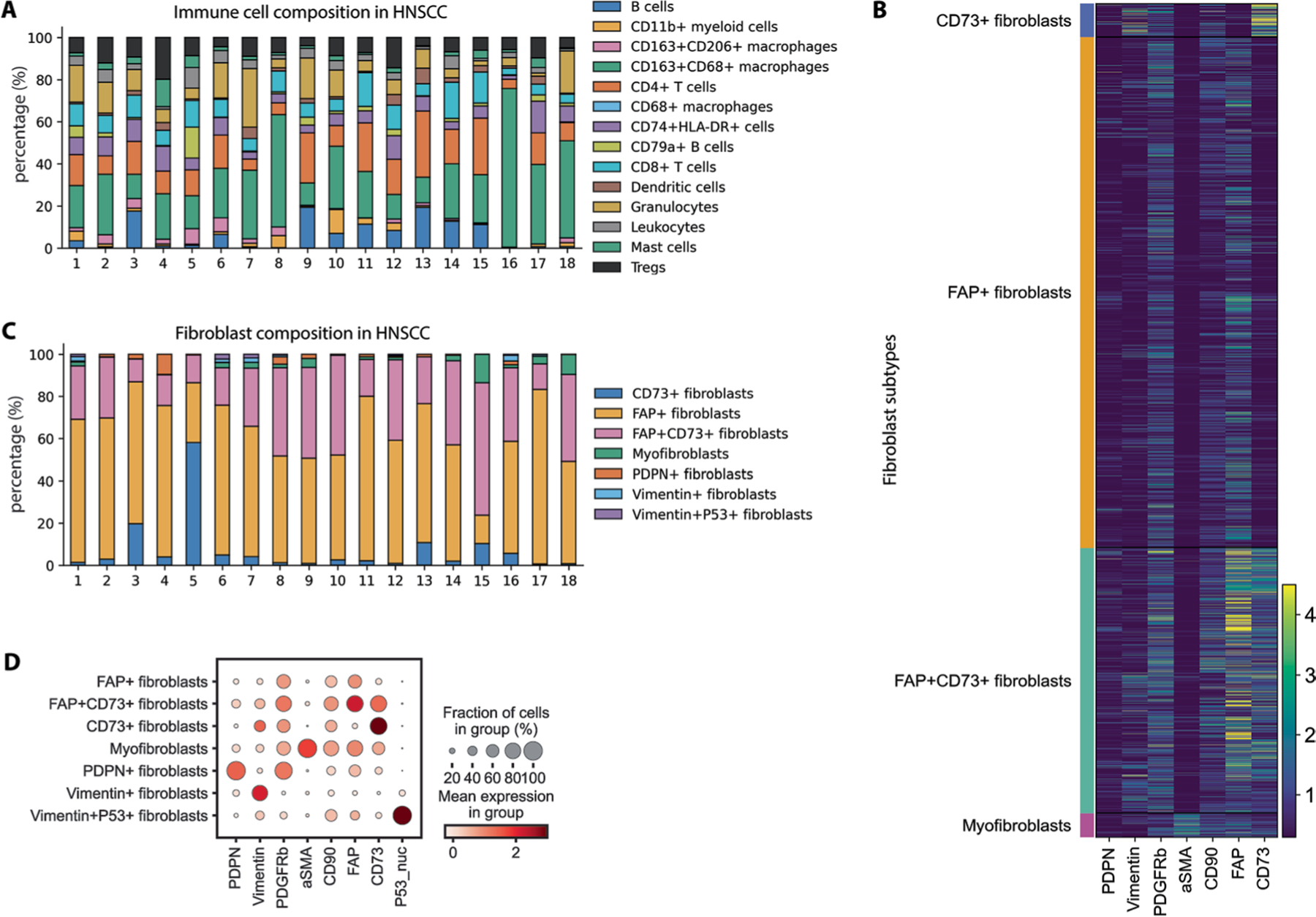
Cell type composition in HNSCC patients. (**a**) Composition of immune cells across HNSCC patients (N = 18 patients). (**b**) Characteristics of distinct fibroblast subsets identified in CODEX imaging of HNSCC tissues. CAFs were classified based on their expression of PDPN, vimentin, PDGFRb, αSMA, CD90, FAP, and CD73 (vimentin+ fibroblasts and vimentin + /P53+ fibroblasts are not shown due to limited cell numbers) (N = 18 patients). (**c**) Composition of fibroblasts across HNSCC patients (N = 18 patients). (**d**) Marker expression of the fibroblast subtypes.

To understand the mechanisms underlying the heterogeneous drug distribution, we applied the SSP method to participants with HNSCC (Supplementary Table 1 and Supplementary Fig. 1a), which included deep spatial profiling of formalin-fixed paraffin-embedded (FFPE) tissue sections using a panel of 50 DNA-barcoded antibodies (Fig. 1g and Supplementary Table 2). The panel was designed to detect tumor cells, CAFs, vasculature, immune cells, ECM proteins, target antigen EGFR and functional states of cells. CODEX multiplexed imaging was performed on tissue microarrays (TMAs) including tissues from 18 participants with HNSCC, followed by single-cell segmentation using MESMER—a pretrained deep learning model^21^. This approach detected over 1 million single cells. Given the presumed importance of stromal components in mediating drug delivery, we evaluated the robustness of cell segmentation by benchmarking MESMER performance against manual expert annotations in fibroblast-rich tissue areas. We analyzed 20 dense stromal regions (HNSCC, *n* = 10; PDAC, *n* = 10), comprising 3,887 cells, using consensus manual delineations from two independent experts as ground truth. In these regions, MESMER achieved high agreement with manual annotations, with an *F*_1_ score of 0.823 ± 0.026 in HNSCC and 0.817 ± 0.026 in PDAC and mean absolute surface distances below 1 μm in both tumor types. These results indicate that MESMER maintains subcellular-scale segmentation accuracy in fibroblast-rich TMEs, supporting its suitability for downstream analyses (Extended Data Fig. 1). Using unsupervised Leiden clustering^22^, we identified 15 cell types at the coarse level and 31 cell types at a fine granularity, according to canonical marker combinations (Fig. 1h and Supplementary Fig. 1b). We annotated tumor, immune and stromal compartments and identified major CAF subtypes (Extended Data Fig. 2 and Supplementary Fig. 1c–g), including FAP^+^ and FAP^+^CD73^+^ CAFs, which are highly reproducible (Supplementary Fig. 1f,g).

**Extended Data Fig. 3 |:**
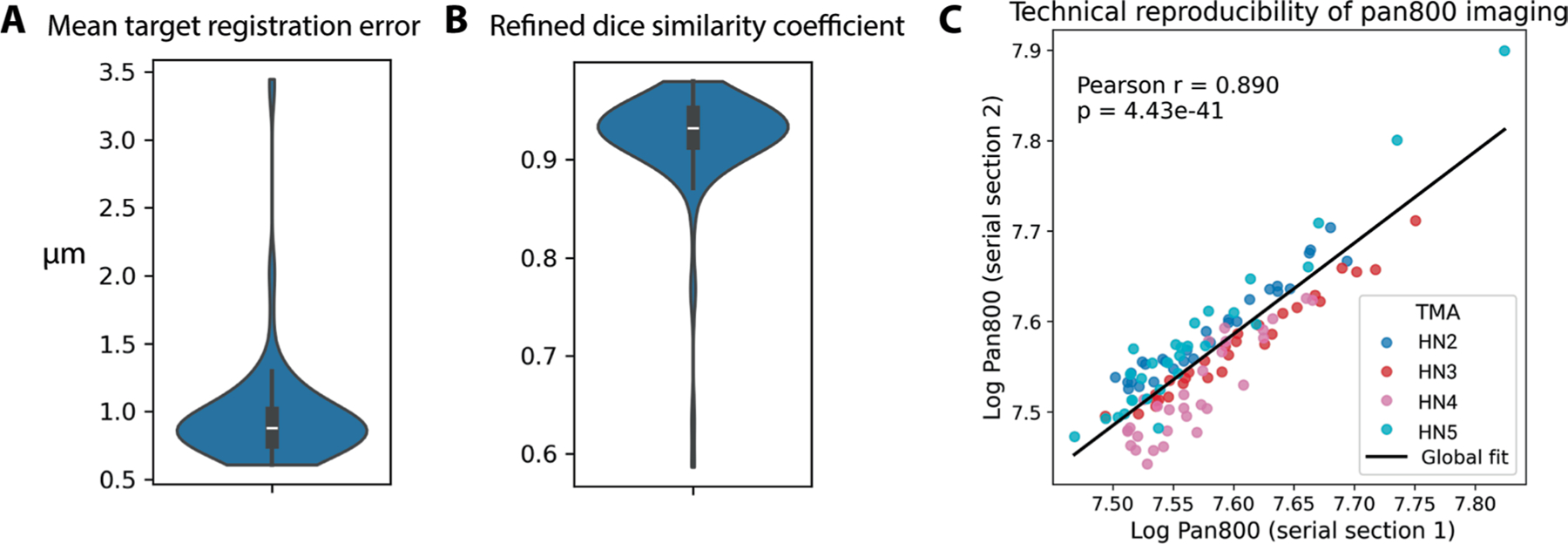
Image registration accuracy and technical reproducibility of pan800 imaging. (**a-b**) Violin plots showing quantitative evaluation of image registration accuracy (n = 111 tissue regions from 18 patients). A: violin plot of the mean target error in μm; B: violin plot of the refined dice similarity coefficient. (**c**) Technical reproducibility of pan800 imaging. Technical reproducibility was assessed using paired serial FFPE sections from the same tissue blocks across four TMAs (117 paired cores including control tissues). All images were acquired using identical microscope settings and stable LED illumination. Internal negative controls and multiple cores per patient were included to monitor within-assay variability. Pan800 quantification was highly consistent between sections (Pearson r = 0.89, two-sided p = 4.4 × 10^−41^), with low section-to-section variability (median coefficient of variation = 2.4%, IQR 1.1–3.9%). In (**a-b**), violin plots represent the distribution of the data estimated by kernel density. The embedded boxplots indicate the median (centre line), interquartile range (box bounds, 25th–75th percentiles), and whiskers extending to the most extreme data points within 1.5× the interquartile range. The minima and maxima correspond to the lowest and highest data points within the whisker range.

To identify specific cell types bound by the therapeutic antibody, we coregistered microscopic images of pan800 with CODEX images of the same tissue slides (Fig. 1i). This approach allowed us to assess the spatial relationships between pan800 and EGFR, vessels (CD31), macrophages (CD163), CAFs (FAP) and ECM (tenascin C and periostin) (Fig. 1j). We were able to visualize the systemically infused therapeutic antibody in participants in the operating room and in single cells in situ from primary HNSCC and metastatic lymph nodes (Fig. 1k,l). Quantitative evaluation of pan800–CODEX image coregistration demonstrated subcellular alignment accuracy (Extended Data Fig. 3a,b). Across 111 regions from pan800 infused participants, the mean target registration error was 0.97 ± 0.41 μm, and nuclear overlap showed a Dice similarity coefficient of 0.92 ± 0.05 with a one-pixel tolerance, indicating high single-cell correspondence. Furthermore, pan800 imaging was highly reproducible across paired serial FFPE sections (Pearson *r* = 0.89, *P* = 4.4 × 10^−41^, median coefficient of variation = 2.4%, *n* = 117 cores across four TMAs including control tissues; Extended Data Fig. 3c).

**Extended Data Fig. 4 |:**
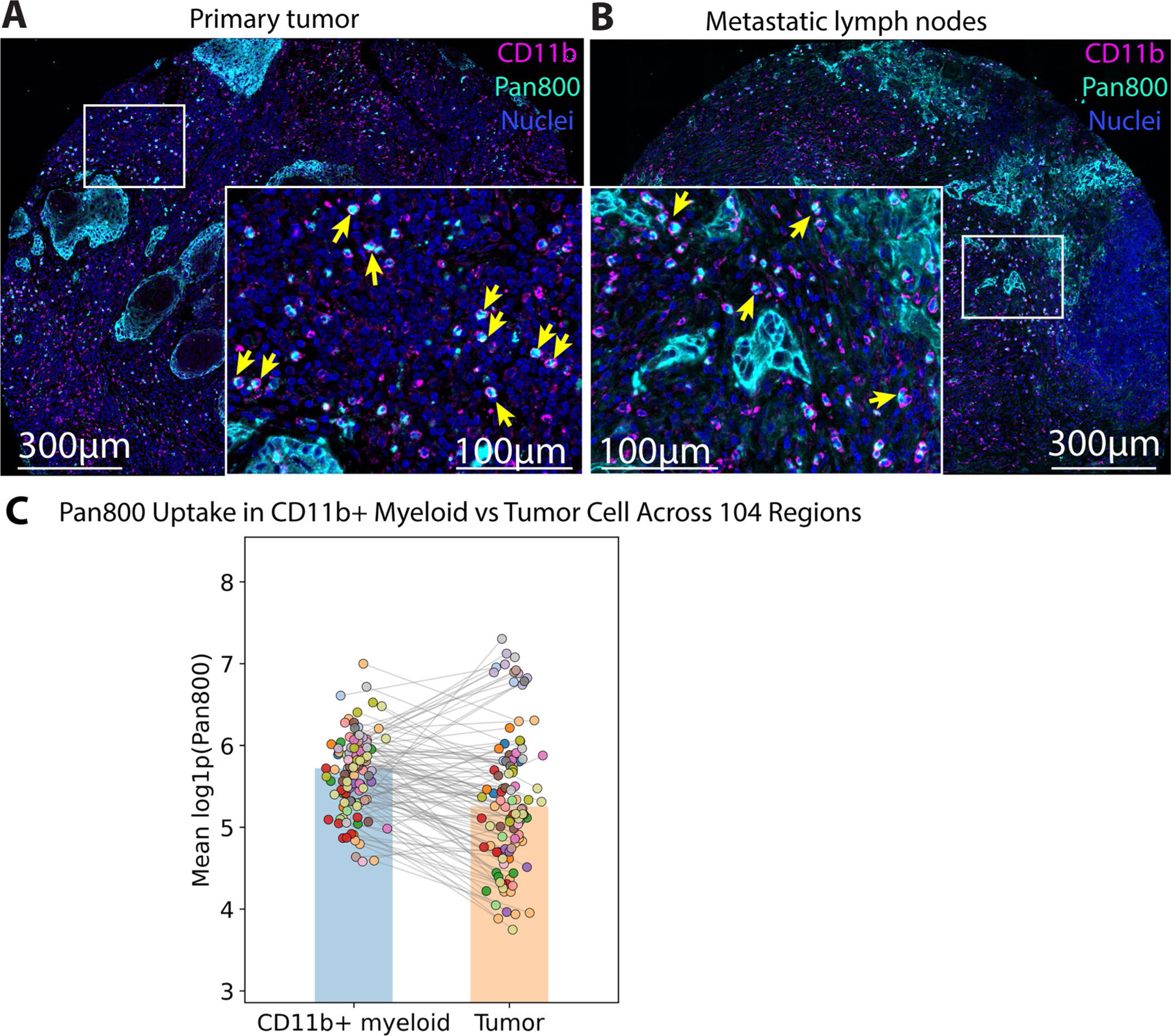
Comparison of pan800 signal between CD11b^+^ myeloid cells and tumor cells in the HNSCC cohort. (**a–b**) Representative overlay images of pan800 fluorescence and CD11b CODEX staining from a primary tumor (**a**) and a metastatic lymph node region (**b**), corresponding to the regions shown in Fig. 1l. Arrows indicate bright pan800 puncta localized within CD11b^+^ myeloid cells in the stromal compartment. (**c**) Bar plots with paired dot overlays showing region-level pan800 fluorescence intensity in CD11b^+^ myeloid cells and tumor cells across all analyzed paired tissue regions (n = 104 regions from 18 patients). Each paired dot represents one tissue region, and paired points are connected within the same region. Bars indicate medians. Pan800 signal was significantly higher in CD11b^+^ myeloid cells than in tumor cells (paired Wilcoxon signed-rank test, two-sided BH-adjusted p = 2.06 × 10^−5^).

While tumor cells exhibited high pan800 signal, we also observed elevated pan800 fluorescence signal in CD11b^+^ myeloid cells relative to tumor and other stromal populations as demonstrated in a representative participant (Fig. 1l–n and Extended Data Fig. 4a,b). Consistent with this example, region-level paired analysis showed significantly higher pan800 signal in CD11b^+^ myeloid cells than in tumor cells in 73 of 104 regions (70.2%) across 18 participants (paired Wilcoxon signed-rank test, two-sided Benjamini–Hochberg (BH)-adjusted *P* = 2.06 × 10^−5^; Extended Data Fig. 4c). At the participant level, myeloid lineages including CD11b^+^ myeloid cells and macrophage subsets ranked among the highest in single-cell pan800 intensity (Fig. 1o; each point represents pan800 signal across all cells of a given type within a participant). Together, these data demonstrate a reproducible enrichment of antibody-associated signal in myeloid populations across multiple participants and regions. While these findings are consistent with a role for myeloid cells in shaping therapeutic antibody distribution and exposure within tumors, we note that alternative explanations, such as uptake of antibody by phagocytic cells, remain possible and will require further mechanistic investigation. Among all participants with HNSCC, we observed considerable heterogeneity in pan800 intensity across cell types among individuals (Fig. 1o). The interparticipant and intraparticipant variability highlights the complex and spatially heterogeneous nature of drug delivery within the TME, emphasizing the need for spatially resolved PK in therapeutic assessment.

### Single-cell antibody–drug target engagement is linked to target cell states and spatial topology in HNSCC

**Extended Data Fig. 5 |:**
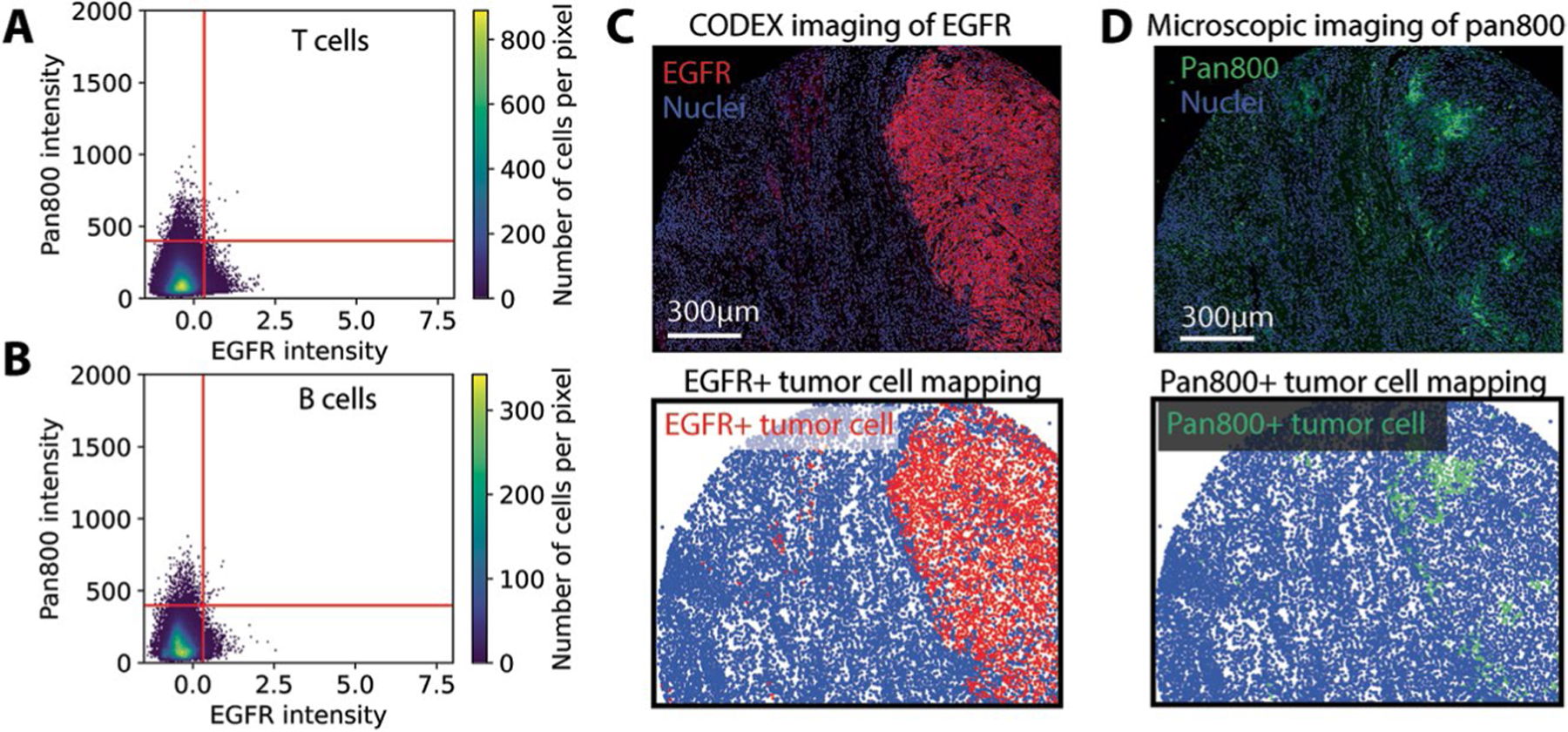
HNSCC tumor EGFR and pan800 thresholding. (**a-b**) Biaxial plots of EGFR and pan800 gating in A) T cells and B) B cells, which were set as references for EGFR and pan800 double-negative cells. (**c**) Representative CODEX image of EGFR staining of HNSCC tumor core and corresponding overlay of EGFR+ cell map. (**d**) Representative microscopic images of systemically infused pan800 and corresponding overlay of pan800+ tumor cell map. Representative images in (**c, d**) are from 89 tumor regions (n = 18 patients). Quantification and statistical analyses were performed across all regions shown in the corresponding plot in Fig. 2c.

**Fig. 2 |:**
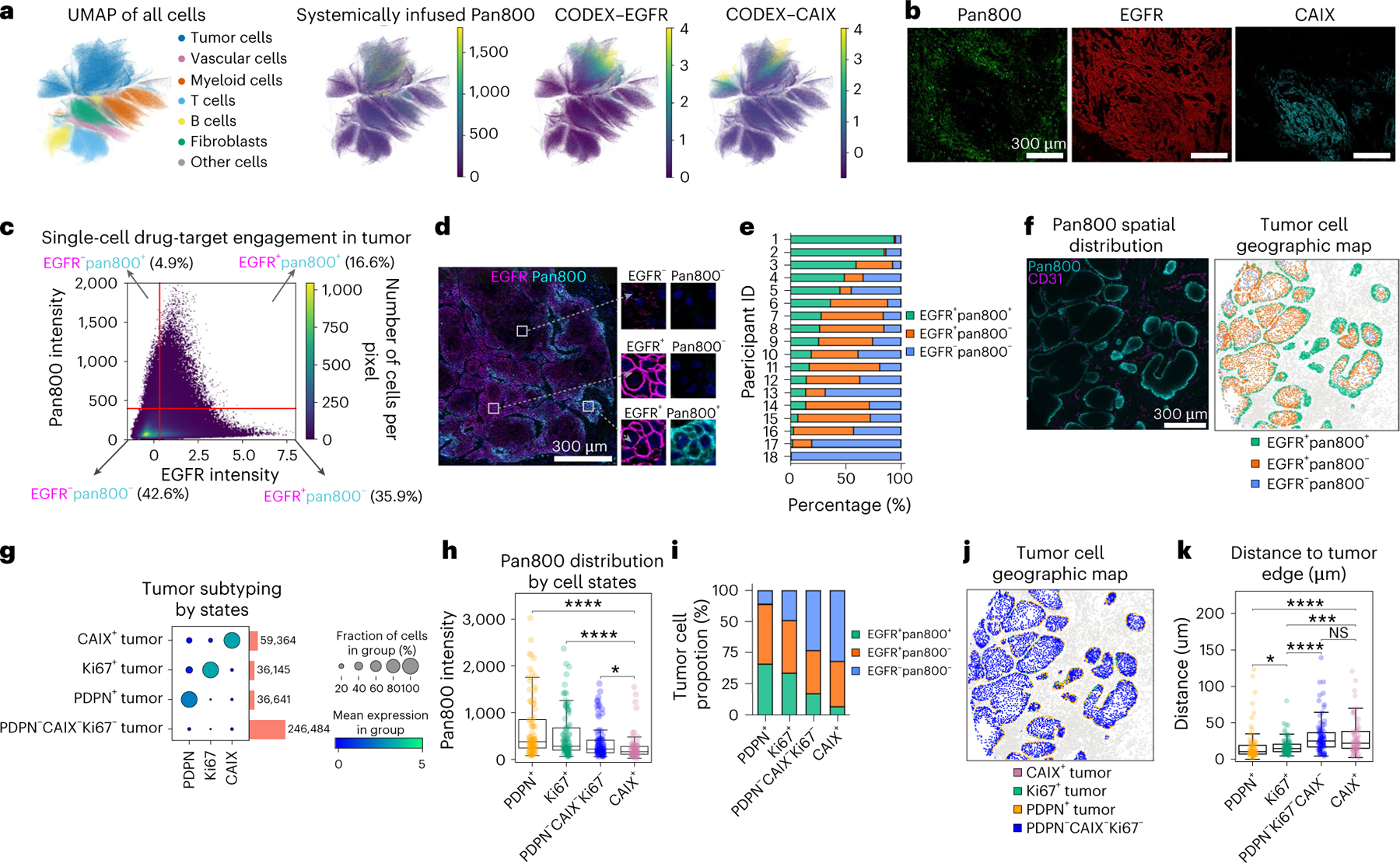
SSP quantifies antibody–target interactions and spatial antibody penetration patterns in the intact TME. **a**, UMAP visualizations of single-cell phenotypes identified across HNSCC tissues (*n* = 18 participants), colored by cell type, pan800 fluorescence intensity, EGFR expression and CAIX expression. **b**, Representative images showing the spatial distribution of pan800, EGFR and CAIX in primary HNSCC tissue. Images are representative of tumor regions analyzed across the cohort (*n* = 89 cores from 18 participants). Visualization of these markers across all cells and regions are shown in **a**. **c**, Biaxial scatter plot of pan800 fluorescence intensity versus EGFR expression for all tumor cells pooled across all participants with HNSCC (*n* = 18 participants), with gates defining EGFR^+^pan800^+^, EGFR^+^pan800^−^ and EGFR^−^pan800^−^ tumor cell populations. **d**, Representative images illustrating the spatial coexistence of EGFR^+^pan800^+^, EGFR^+^pan800^−^ and EGFR^−^pan800^−^ tumor regions within the same tumor. Images are representative of tumor regions analyzed across the cohort (*n* = 89 cores from 18 participants). Quantification analysis was performed across all regions and participants as shown in **e**. **e**, Percentages of EGFR^+^pan800^+^, EGFR^+^pan800^−^, EGFR^−^pan800^−^ and EGFR^−^pan800^+^ tumor cells in primary HNSCC tissues across participants (*n* = 18). **f**, Representative images showing the spatial distribution maps of the localization of EGFR^+^pan800^+^, EGFR^+^pan800^−^ and EGFR^−^pan800^−^ tumor cells. Images are representative of tumor regions analyzed across the cohort (*n* = 89 cores from 18 participants). Quantification and statistical analyses were performed across all regions and are shown in Extended Data Fig. 6b. **g**, Dot plot summarizing expression of tumor markers Ki67, CAIX and PDPN across four tumor cell expression programs identified from single-cell analysis (*n* = 18 participants). Dot size indicates the fraction of cells expressing the marker, and color denotes mean normalized expression. **h**, Comparison of pan800 fluorescence intensity across the four tumor subtypes at the region level. Each dot represents one tissue region (TMA core; *n* = 89 cores from 18 participants). Statistical comparisons were performed using two-tailed Mann–Whitney *U*-tests with Bonferroni correction: PDPN^−^CAIX^−^Ki67^−^ versus CAIX^+^, *P* = 1.52 × 10^−2^; Ki67^+^ versus CAIX^+^, *P* = 9.63 × 10^−6^; PDPN^+^ versus CAIX^+^, *P* = 2.54 × 10^−8^. *P < 0.05, **P < 0.01, ***P < 0.001 and ****P < 0.0001. **i**, Percentages of EGFR^+^pan800^+^, EGFR^+^pan800^−^ and EGFR^−^pan800^−^ tumor cells within each of the four tumor subpopulations (*n* = 18 participants). **j**, Spatial distribution maps showing the localization of CAIX^+^, Ki67^+^, PDPN^+^ and PDPN^−^CAIX^−^Ki67^−^ tumor cell programs within a representative tumor region. **k**, Distances from individual tumor cells to the nearest tumor nest boundary, stratified by tumor subtypes (PDPN^+^, Ki67^+^, PDPN^−^CAIX^−^Ki67^−^ and CAIX^+^), quantified across tissue regions (*n* = 89 TMA cores from 18 participants). Statistical comparisons were performed using two-tailed Mann–Whitney *U*-tests with Bonferroni correction: PDPN^+^ versus Ki67^+^, *P* = 2.62 × 10^−2^; Ki67^+^ versus PDPN^−^CAIX^−^Ki67^−^, *P* = 5.75 × 10^−6^; PDPN^−^CAIX^−^Ki67^−^ versus CAIX^+^, *P* = 1.00; Ki67^+^ versus CAIX^+^, *P* = 6.79 × 10^−4^; PDPN^+^ versus CAIX^+^, *P* = 4.54 × 10^−6^. *P < 0.05, **P < 0.01, ***P < 0.001 and ****P < 0.0001. Box plots in **h**,**k** show the median (center line), interquartile range (box bounds, 25th–75th percentiles) and whiskers extending to the minimum and maximum values within 1.5× the interquartile range from the lower and upper quartiles, respectively. Data points beyond this range are not shown.

Although single-cell technologies such as flow cytometry are often used to measure drug–target engagement in dissociated cells, these methods lack the ability to map the geospatial distribution of the drug across the tumor mass. SSP enables measurement of spatially resolved antibody–target engagement at the single-cell level. We analyzed about 1,000,000 cells segmented from CODEX images of the HNSCC tissues, projecting them into a two-dimensional uniform manifold approximation and projection (UMAP) plot (Fig. 2a). This analysis revealed considerable heterogeneity in pan800 distribution and EGFR expression across tumor cells. In a representative HNSCC region (Fig. 2b), pan800 accumulated at the tumor periphery despite relatively uniform EGFR expression, while the CAIX (carbonic anhydrase IX)^+^ hypoxic region showed low pan800 signal (Fig. 2b). By stratifying tumor cells based on EGFR expression and pan800 binding, we uncovered substantial variations in drug–target engagement across tumor cells (*n* = 378,634 tumor cells; Fig. 2c), tissue regions (Fig. 2d) and participants (Fig. 2e). Among all tumor cells across 18 participants, only 16.6% of tumor cells were both EGFR^+^ and pan800^+^, while 35.9% expressed EGFR but were negative for pan800 and 42.6% lacked EGFR expression and pan800 binding (Fig. 2c). The threshold for defining EGFR^+^ and pan800^+^ cells was determined using cell types that are EGFR-negative and pan800-negative (for example, T cells and B cells) as a reference (Extended Data Fig. 5a,b). The gated cells were also mapped to CODEX images for further validation (Extended Data Fig. 5c,d). Sensitivity analysis using ±10% shifts in the EGFR and pan800 intensity thresholds relative to the original cutoff values resulted in only minor changes in the fraction of EGFR^+^pan800^+^ tumor cells (Supplementary Fig. 2a), indicating that EGFR^+^pan800^+^ proportions are robust to reasonable threshold selection. We observed a small EGFR^−^pan800^+^ tumor cell population (4.9% of all tumor cells across the cohort). Because EGFR positivity was defined relative to immune cell references (T and B cells), a subset of low-EGFR tumor cells may fall below the EGFR^+^ gate yet retain measurable pan800 signal (representative examples in Supplementary Fig. 2b–d).

**Extended Data Fig. 6 |:**
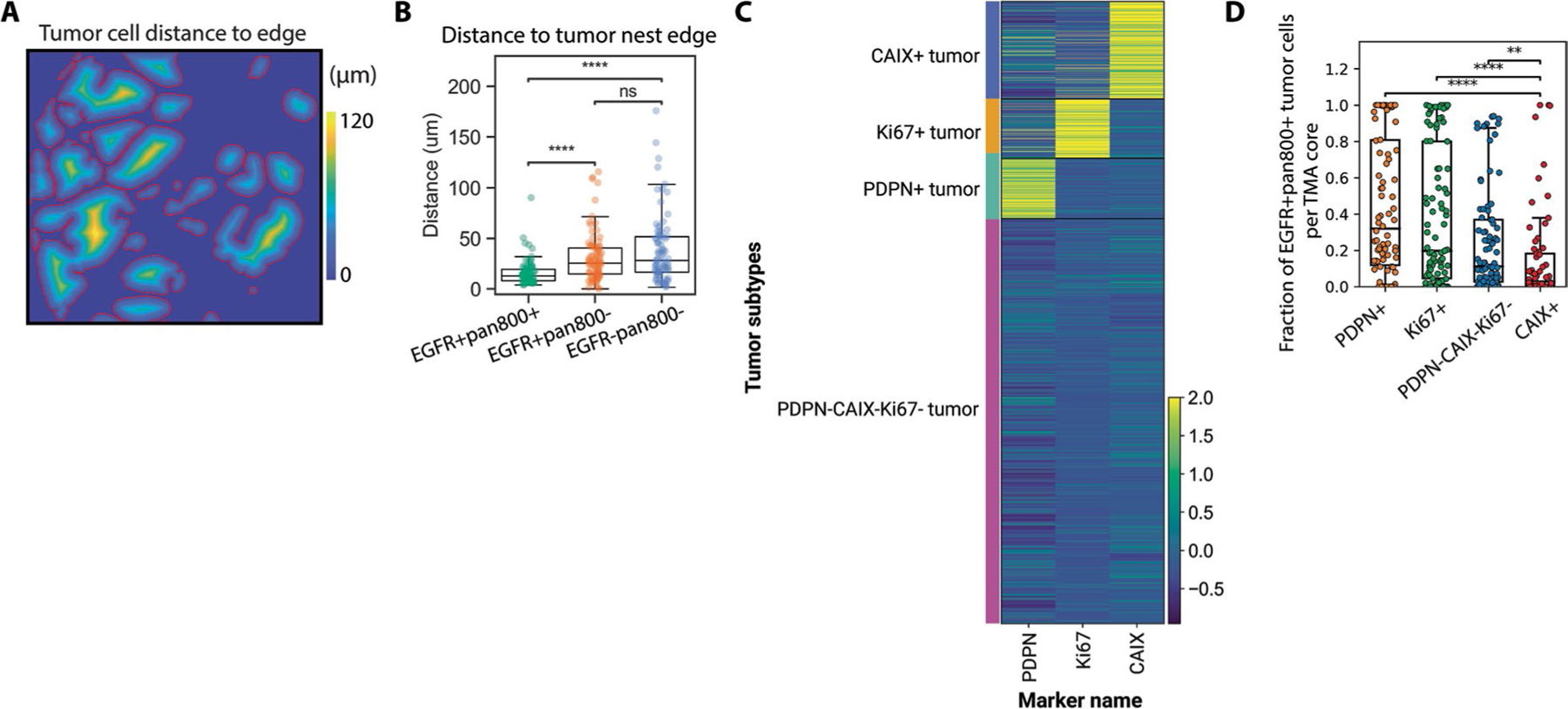
HNSCC tumor subtype analysis. (**a**) Euclidean distance map of pixels to their tumor nest edges for the tissue region in Fig. 2f. (**b**) Distances of EGFR^+^/pan800^+^, EGFR^+^/pan800^−^, and EGFR^−^/pan800^−^ tumor cells to tumor nest edges across TMA cores in primary HNSCC tissues (N = 89 TMA cores from 18 patients). Statistical comparisons were performed using two-sided Mann–Whitney U tests with Bonferroni correction: EGFR^+^pan800^+^ vs EGFR^+^pan800^−^, p = 1.04 × 10^−6^; EGFR^+^pan800^−^ vs EGFR^−^pan800^−^, p = 0.450; EGFR^+^pan800^+^ vs EGFR^−^pan800^−^, p = 3.85 × 10^−9^. ns = not significant; * p < 0.05, ** p < 0.01; *** p < 0.001, **** p < 0.0001. (**c**) Single-cell expression heatmap showing PDPN, Ki67, and CAIX across tumor cells. Color shows normalized protein expression. While individual cells may express low levels of multiple markers, clustering reflects dominant expression programs rather than strict marker exclusivity. (**d**) CAIX^+^ tumor cells exhibit the lowest fraction of EGFR^+^/ pan800^+^ cells. Each dot represents one TMA core. Statistical comparison was performed using two-sided Mann–Whitney U tests with Benjamini–Hochberg correction: PDPN^+^ tumor vs CAIX^+^ tumor, p = 2.30 × 10^−6^; Ki67^+^ tumor vs CAIX^+^ tumor, p = 5.89 × 10^−5^; PDPN^−^/CAIX^−^/Ki67^−^ tumor vs CAIX^+^ tumor, p = 0.002. Significance is indicated by asterisks: ***p < 0.001; ****p < 0.0001). Box plots in (**b, d**) show the median (center line), interquartile range (box bounds, 25th–75th percentiles), and whiskers extending to the minimum and maximum values within 1.5× the interquartile range from the lower and upper quartiles, respectively. Data points beyond this range are not shown.

To evaluate the geographic distribution of the drug–target engagement, we spatially mapped EGFR^+^pan800^+^, EGFR^+^pan800^−^ and EGFR^−^pan800^−^ tumor cells at single-cell resolution. We observed that EGFR^+^pan800^+^ tumor cells were predominantly located at the leading edge of tumor nests near blood vessels, whereas EGFR^+^pan800^−^ tumor cells were more frequently found in the core of tumor nests (Fig. 2f). This distribution pattern is consistent with the ‘binding-site barrier’ hypothesis, which postulates that there is a stalled saturation front in tumors that can be exacerbated by low drug dose, high antigen density and high binding affinity^23^. To quantify this effect, we segmented individual tumor nests and spatially mapped the Euclidean distance of each pixel to the edge of its tumor nest (Extended Data Fig. 6a). We found that the EGFR^+^pan800^+^ tumor cells were significantly closer to the tumor nest edge than both EGFR^+^pan800^−^ and EGFR^−^pan800^−^ tumor cells (two-tailed Mann–Whitney *U*-test, *P* < 0.0001, *n* = 89 cores from 18 participants; Extended Data Fig. 6b).

To explore whether tumor cell states influence drug–target engagement, we conducted unsupervised clustering to classify tumor cells based on expression of Ki67 (proliferation marker), CAIX (hypoxia marker) and PDPN (partial epithelial–mesenchymal transition, pEMT marker). This analysis identified four dominant tumor cell expression programs characterized by high PDPN, Ki67 or CAIX expression, as well as a population with low expression of all three markers (Fig. 2g and Extended Data Fig. 6c). Tumor cells in the states of pEMT (PDPN^+^) and growth (Ki67^+^) exhibited the highest levels of pan800 binding, whereas tumor cells with low metabolic activities (CAIX^+^) displayed the lowest pan800 binding (two-tailed Mann–Whitney *U*-test, *P* < 0.0001, *n* = 89 TMA core from 18 participants; Fig. 2h). Single-cell analysis further confirmed that the CAIX^+^ tumor subtypes had the lowest proportion of EGFR^+^pan800^+^ tumor cells (two-tailed Mann–Whitney *U*-test, *P* < 0.01, *n* = 89 TMA cores from 18 participants; Fig. 2i and Extended Data Fig. 6d), consistent with reduced antibody access in hypoxic tumor states^24,25^.

In line with this, we found that PDPN^+^ tumor cells and Ki67^+^ tumor cells were preferentially located in the periphery of tumor nests, whereas CAIX^+^ tumor cells and PDPN^−^CAIX^−^Ki67^−^ tumor cells were farther away from the tumor nest edges (two-tailed Mann–Whitney *U*-tests with Bonferroni correction, *P* < 0.05 for PDPN^+^ versus Ki67^+^; *P* < 0.0001 for Ki67^+^ versus PDPN^−^CAIX^−^Ki67^−^; *P* = 1.00 for PDPN^−^CAIX^−^ Ki67^−^ versus CAIX^+^; *P* < 0.001 for Ki67^+^ versus CAIX^+^; *P* < 0.0001 for PDPN^+^ versus CAIX^+^; Fig. 2j,k).

### Periostin-rich ECM correlates with reduced antibody penetration into HNSCC

We next sought to identify the TME architectural barriers to drug penetration in participants with HNSCC. Previous studies have primarily characterized the TME architecture in terms of cells and multicellular modules^26–30^; the spatial organization of ECM and its impact on drug distribution remain poorly understood. The study of drug delivery barriers in solid tumors is currently limited to bulk tissue measurements such as collagen and hyaluronan concentrations^31,32^ and tissue elasticity^33^. We hypothesized that specific ECM assemblies surrounding tumor nests and endothelial cells may serve as physical barriers to antibody penetration. To test this, we spatially profiled major ECM proteins, including collagen I, collagen IV, fibronectin, periostin and tenascin C, alongside pan800 and markers of cell phenotypes and states within the same tissue sections.

**Fig. 3 |:**
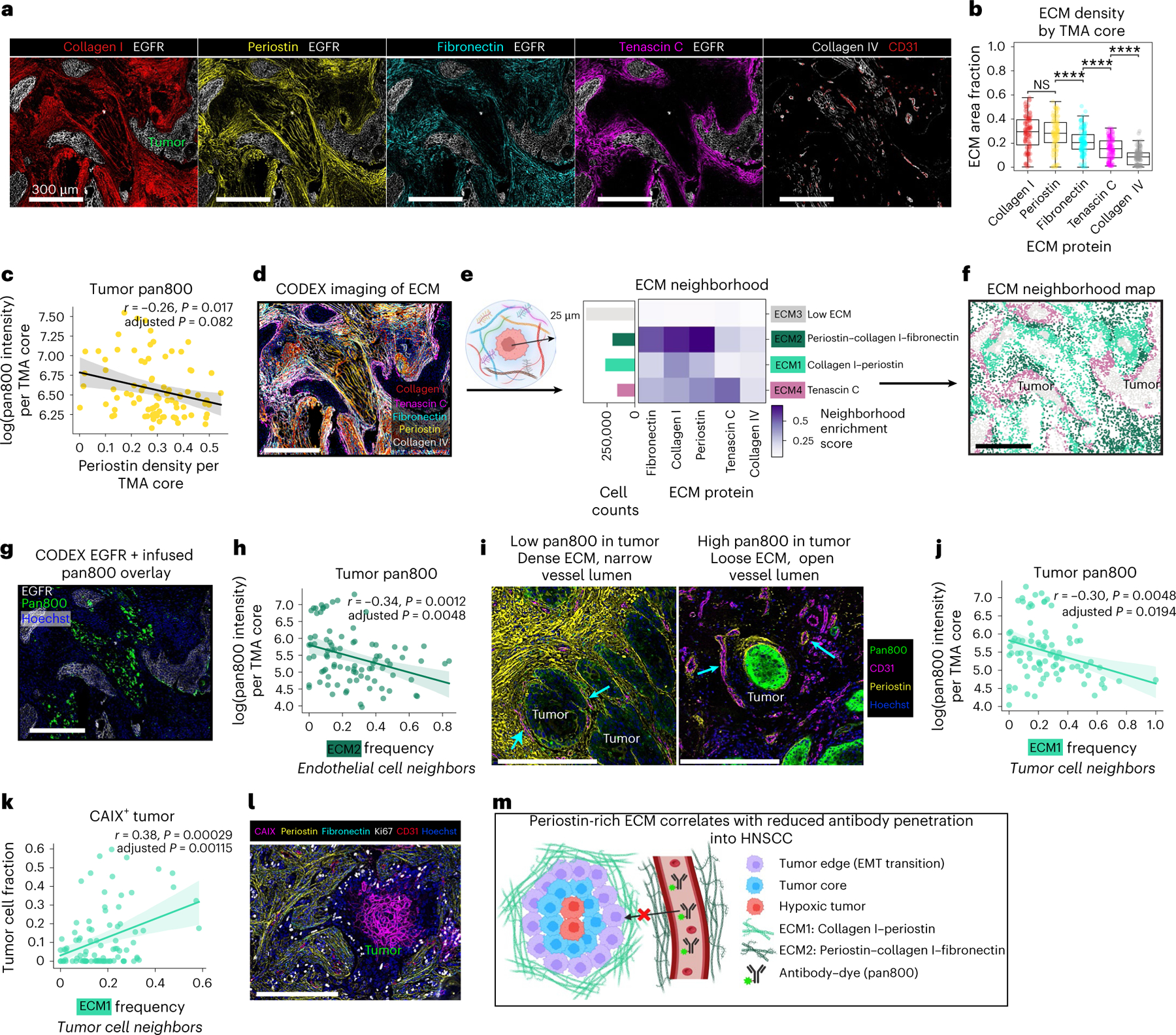
Periostin-rich ECM correlates with reduced antibody penetration into HNSCC tumors. **a**, Representative images showing the spatial distribution of five ECM proteins in a primary HNSCC tumor region. Scale bar, 300 μm. **b**, Box plots summarizing the area fractions of five ECM proteins by TMA cores (TMA cores; *n* = 111 cores from 18 participants). Each dot represents a TMA core. Box plots show the median (center line), interquartile range (box bounds, 25th–75th percentiles) and whiskers extending to the minimum and maximum values within 1.5× the interquartile range from the lower and upper quartiles, respectively. Data points beyond this range are not shown. Statistical comparisons were performed using two-tailed Mann–Whitney *U*-tests with Bonferroni correction: Collagen I versus periostin, *P* = 1.00; periostin versus fibronectin, *P* = 6.80 × 10^−5^; fibronectin versus tenascin C, *P* = 8.52 × 10^−5^; tenascin C versus collagen IV, *P* = 6.01 × 10^−8^. *P < 0.05, **P < 0.01, ***P < 0.001 and ****P < 0.0001. **c**, Scatter plot showing the linear correlation between periostin area fraction and the log-transformed tumor pan800 intensity at the regional level. Each point represents one TMA core containing EGFR^+^pan800^+^ tumor cells (*n* = 85 cores from 18 participants). **d**, Composite image of five ECM proteins for the primary tumor sample in **a**. Scale bar, 300 μm. **e**, Identification of four distinct ECM neighborhoods based on the area fraction of the five ECM proteins evaluated within the 25-μm radius of each anchor cell (*n* = 18). The cell illustration was created with BioRender. **f**, Spatial map of the ECM neighborhoods for tissue region in **d**. **g**, Overlay of pan800 and EGFR signals aligned with the ECM map shown in **f**. Scale bar, 300 μm. **h**, Scatter plot of log-transformed tumor pan800 intensity versus frequency of ECM2 in the neighborhood of endothelial cells (*n* = 89 TMA cores from 18 participants). **i**, Representative images showing reduced vessel luminal area in dense ECM with low drug delivery in tumor (left) and rounded vessel lumens in ECM-loose stroma with high drug delivery in tumor cells (right). Representative images from 89 tumor regions (*n* = 18 participants). Quantification and statistical analyses were performed across all regions shown in the corresponding plot (**h**). Scale bar, 300 μm. **j**, Scatter plots of the linear correlations between log-transformed tumor pan800 intensity and ECM1 frequency around tumor cells (*n* = 85 TMA cores from 18 participants). **k**, Scatter plot of CAIX^+^ tumor cell fraction versus ECM1 frequency around tumor cells (*n* = 89 TMA cores from 18 participants). **l**, Representative region of hypoxic tumor encapsulated by dense layers of periostin and fibronectin. Representative images from 89 tumor regions (*n* = 18 participants). Quantification and statistical analyses were performed across all regions shown in the corresponding plot (**k**). Scale bar, 300 μm. **m**, Schematic illustration of the ECM neighborhoods associated with reduced therapeutic antibody delivery into HNSCC TME. The schematic was created with BioRender. For all scatter plots (**c**,**h**,**j**,**k**), associations were assessed using Pearson’s correlation, with two-tailed *P* values adjusted for multiple comparisons using the BH method. The scatter plots are displayed with linear regression fits and 95% confidence intervals indicated by shaded areas.

We observed that different ECM proteins have distinct spatial distribution patterns that likely relate to their cellular origin and cell–matrix interactions (Fig. 3a). While collagen I, periostin and fibronectin were broadly distributed throughout the tumor stroma, fibronectin and periostin sometimes formed a close lining around endothelial cells. We also observed linearization and alignment of collagen I, periostin, fibronectin and tenascin C in the interstitial matrix adjacent to tumor cells, suggesting ECM stiffening during tumorigenesis^34^. By segmenting ECM from CODEX images (Supplementary Fig. 3a), we quantified ECM density normalized by the extracellular tissue area in each TMA core. We found heterogeneous distributions of ECM proteins across participants (Supplementary Fig. 3b) and tissue regions (Fig. 3b). Among the ECM components analyzed, periostin and collagen I densities were significantly higher than densities of fibronectin, tenascin C and collagen IV (Supplementary Fig. 3b). Notably, periostin displayed a strong peritumor and perivascular localization, distinct from collagen I (Supplementary Fig. 3c). Among all five ECM proteins analyzed, periostin was the only one that showed a significant inverse correlation with the pan800 intensity (Pearson *r* = −0.26, *P* = 0.017, *n* = 85 TMA cores from 18 participants; Fig. 3c), suggesting that periostin-rich ECM may act as a barrier to therapeutic antibody penetration.

We reasoned that these ECM proteins do not function independently but instead assemble into distinct pericellular structures that regulate drug accessibility. To systematically assess ECM organization, we developed an algorithm to categorize the pericellular ECM neighborhoods. We defined the ECM neighborhood of each cell as a circular window (25-μm radius) centered on each cell and calculated the area fractions of collagen I, collagen IV, fibronectin, tenascin C and periostin within these windows (Fig. 3d,e). The 25-μm radius was chosen empirically to provide good spatial granularity. Next, we performed unsupervised clustering on the basis of the ECM area fractions and identified four distinct ECM compositions or neighborhoods clusters (Fig. 3e and Supplementary Fig. 3d,e). ECM1 was enriched in collagen I and periostin, ECM2 was enriched in periostin, collagen I and fibronectin, ECM3 had low density of all markers and ECM4 was enriched in tenascin C. Among these, ECM2 exhibited the highest density of ECM proteins, suggesting a physically restrictive ECM structure. Different ECM neighborhoods were located in spatially distinct regions in relation to tumor cells and to pan800 (Fig. 3f,g). We further demonstrated that ECM neighborhood identification is robust to clustering resolution (*k* = 4–6; Supplementary Fig. 4a–e), clustering method (*k*-means versus hierarchical clustering) and neighborhood radius (12.5, 25, 50 and 100 μm; Supplementary Fig. 4f–i).

To evaluate whether ECM organization impacts drug delivery, we examined the relationships between perivascular and peritumoral ECM neighborhoods and tumor pan800 levels. At perivascular sites, the frequency of ECM2 (enriched in periostin, collagen I and fibronectin) was inversely correlated with the pan800 uptake in tumor cells (Pearson’s *r* = −0.34, BH-adjusted *P* = 0.0048, *n* = 89 TMA cores from 18 participants; Fig. 3h). This suggests that ECM2 structures around endothelial cells restrict drug transport from blood vessels into the tumor parenchyma. In representative regions, vessels embedded within dense periostin-rich ECM exhibited reduced vascular area compared to vessels in ECM-low stroma (Fig. 3i). Consistent with these observations, region-based analysis across TMA cores spanning the HNSCC cohort revealed a significant inverse association between tumor-adjacent vessel area fraction and perivascular periostin density (Pearson *r* = −0.385, *P* < 0.01, *n* = 102 TMA cores containing tumor and vessels; Supplementary Fig. 5). Together with the reduced tumor drug delivery observed in periostin-rich regions, these findings indicate that periostin-rich ECM is associated with both reduced tumor-adjacent vascular area and impaired antibody access. At peritumoral sites, we found that tumor cells encapsulated by dense ECM1 neighborhoods (ECM rich in collagen I and periostin) exhibited lower pan800 intensity (Pearson’s *r* = −0.30, BH-adjusted *P* = 0.0194, *n* = 85 TMA cores from 18 participants; Fig. 3j), suggesting that dense ECM1 assembly may obstruct drug accessibility to tumor cells. Additionally, we found that ECM1-enriched regions contained a higher percentage of CAIX^+^ tumor cells (Pearson’s *r* = 0.38, BH-adjusted *P* = 0.0012, *n* = 89 TMA cores from 18 participants; Fig. 3k), indicating that ECM1 structures around tumor cells may contribute to hypoxia and restricted drug diffusion (Fig. 3l).

To assess potential batch effects across TMAs and coverslips, we evaluated cell-level and region-level structure following TMA-specific *z* normalization (Supplementary Fig. 6). UMAP embeddings colored by TMA identity showed no segregation into TMA-specific clusters, with cells from all four TMAs intermingled throughout the embedding (Supplementary Fig. 6a). The only partial separation observed corresponded to a biologically distinct hypoxic tumor subtype rather than TMA identity (Supplementary Fig. 6b). Consistent with this, representative region-level scatter plots colored by TMA identifier showed no stratification by slide or batch, with cores from different TMAs similarly interspersed (Supplementary Fig. 6c,d). Together, these analyses indicate that batch effects across TMAs were minimal after normalization and that the observed structure reflects biological rather than technical variation.

Collectively, these data indicate that spatial composition and localization of ECM neighborhoods have functional implications for drug delivery. Our analysis revealed that periostin-rich ECM assemblies are associated with reduced antibody penetration into HNSCC tumors (Fig. 3m). These results provide a framework for investigation of stroma-modulating strategies to improve therapeutic antibody delivery in solid tumors.

### FAP^+^ CAFs correlate with increased periostin-rich ECM and reduced antibody delivery in HNSCC

CAFs are major producers of ECM proteins and have been shown to modulate cancer progression and treatment response^35^. Single-cell RNA sequencing (scRNA-seq) studies have identified distinct CAF subtypes in the TME^36^, yet how these subtypes are spatially arranged within the TME and how their spatial organization influences ECM and drug delivery remain largely unexplored. Given the localization of ECM structures adjacent to tumor and endothelial cells, we hypothesized that peritumoral and perivascular CAFs contribute to ECM deposition and modification, which create physical barriers to antibody penetration.

**Fig. 4 |:**
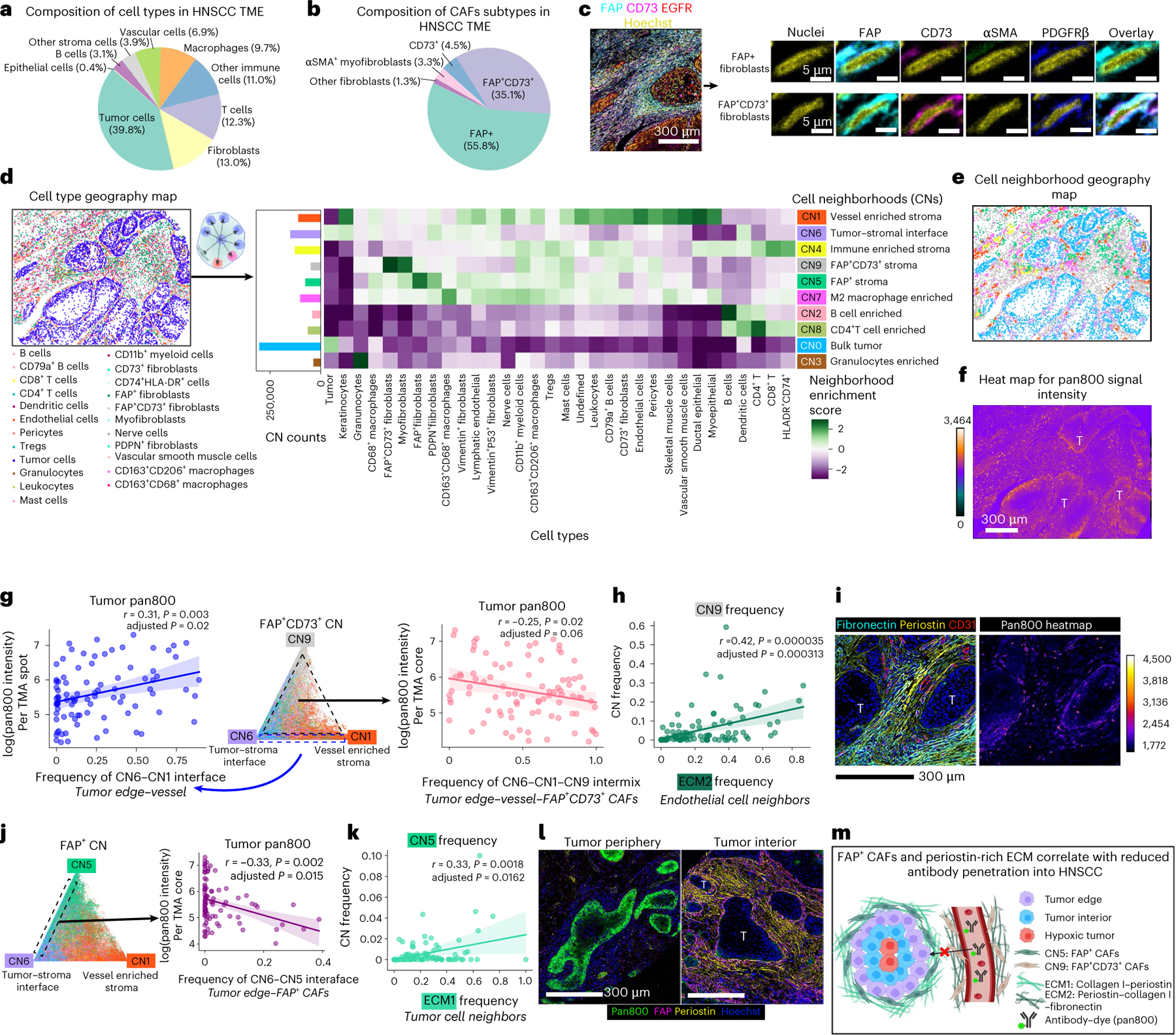
FAP^+^ CAFs are associated with increased periostin-rich ECM and reduced drug–target binding in HNSCC. **a**, Pie chart showing cell type compositions of the TME across *n* = 18 participants with HNSCC. **b**, Pie chart showing relative abundance of CAF subtypes across the same cohort (*n* = 18 participants). **c**, Representative images showing FAP^+^ fibroblasts and FAP^+^CD73^+^ fibroblasts in HNSCC tissue. Representative high-magnification views of fibroblast phenotypes observed across 18 participants with HNSCC. **d**, Left, a representative spatial map of cell phenotypes; Right, a heat map of cell type frequencies in the ten distinct CNs, along with total counts of each CN across the HNSCC cohort (*n* = 18 participants). The cellular illustration was created with BioRender. **e**, Spatial map of CN assignments corresponding to the cell type map shown in **d**. **f**, Heat map of pan800 fluorescence intensity aligned with the CN spatial map shown in **e**. **g**, Left, scatter plot showing the association between log-transformed pan800 intensity and the frequency of CN6–CN1 interfaces per tissue region (TMA core). Middle, barycentric coordinate projection of spatial windows enriched for CN1, CN5 or CN9. Right, scatter plot showing the association between log-transformed pan800 intensity and the frequency of CN6–CN9–CN1 intermixing per TMA core (*n* = 88 cores from 18 participants). Points in the barycentric projection are colored by CN assignment. **h**, Scatter plot showing the association between CN9 frequency and ECM2 neighborhood frequency in the perivascular region, quantified per TMA core (*n* = 89 cores from 18 participants). **i**, Representative CODEX marker overlays with corresponding pan800 heat maps. ‘T’ indicates tumor nests. Representative images from 89 tumor regions (*n* = 18 participants) are shown. Quantification and statistical analyses were performed across all regions shown in the corresponding plot (**h**). **j**, Left, barycentric coordinate projection of spatial windows enriched for CN1, CN5 or CN6. Right, scatter plot showing the association between log-transformed pan800 intensity and the frequency of CN6–CN5 interfaces per TMA core (*n* = 86 cores from 18 participants). Points in the barycentric projection are colored by CN assignment. **k**, Scatter plot of frequencies of CN5 versus ECM1 in the neighborhood of tumor cell per TMA core (*n* = 85 cores from 18 participants). **l**, Representative overlay images of pan800, FAP and periostin for tumor periphery and interior regions. ‘T’ indicates tumor nests. Representative images from 18 participants with HNSCC. Quantification and statistical analyses were performed across all tissues shown in the corresponding plot (**j**,**k**). **m**, Summary illustration depicting the peritumoral and perivascular ECM and CAF neighborhoods that act as barriers to drug delivery in persons with HNSCC. The illustration was created with BioRender. For all scatter plots (**g**,**h**,**j**,**k**), associations were assessed using Pearson’s correlation, with two-tailed *P* values adjusted for multiple comparisons using the BH method. The scatter plots are displayed with linear regression fits and 95% confidence intervals indicated by shaded areas.

In primary HNSCC tumors, the most abundant stromal cells characterized were fibroblasts (13.0%), T cells (12.3%), macrophages (9.7%) and vascular cells (6.9%) (Fig. 4a). Among the 104,647 fibroblast cells segmented from primary tumors, the two predominant CAF subtypes were FAP^+^ CAFs and FAP^+^CD73^+^ CAFs (Fig. 4b,c).

To systematically map CAFs and their interaction with other TME components, we used a spatial analysis method based on cellular neighborhoods (CNs), which define recurring patterns of spatially proximal cells^27,37^. Briefly, we raster-scanned each cell and its *n* − 1 nearest neighboring cells with a window of size *n* cells, followed by unsupervised clustering based on cell type frequencies within each window (Fig. 4d). CN identification was evaluated across window sizes of 5–25 nearest cells, with window sizes of 9 providing optimal separation of tumor, vessel-associated and FAP^+^ fibroblast neighborhoods without oversmoothing in the HNSCC cohort (Supplementary Fig. 7a). Ten CNs (Supplementary Fig. 7b,c) were identified in HNSCC tissue using a window size of 9. The identified CNs included two tumor CNs (CN0, bulk tumor; CN6, tumor–stromal interface), one vessel-enriched stromal neighborhood CN1, two fibroblast CNs (CN5 and CN9, enriched in FAP^+^ CAFs and FAP^+^CD73^+^ CAFs, respectively) and five immune cell CNs (CN3, granulocyte enriched; CN2, B cell enriched; CN7, M2 macrophage enriched; CN8, CD4^+^ T cell enriched; CN4, immune enriched). Each CN was spatially mapped onto the original images (Fig. 4e) and aligned with pan800 images to evaluate their influence on drug penetration (Fig. 4f). As expected, higher levels of CN1, the vessel-enriched stromal neighborhood surrounding tumor nests, correlated with higher drug binding (Supplementary Fig. 7d).

To assess whether interactions among CNs influence drug–target engagement and ECM organization, we examined higher-order multicellular structures, formed by recurring CN–CN interaction within the TME^37^. To quantify these interactions, we extracted windows containing the 180 nearest spatially neighboring cells around each index cell. We then selected cells whose windows consisted predominantly of cells allocated to CN6 (tumor–stromal interface), CN1 (vessel-enriched) and either CN5 (FAP^+^ stroma) or CN9 (FAP^+^CD73^+^ stroma). The composition of these CNs was then projected into a barycentric coordinate system, which allowed visual interpretation of spatial relationships at the single-cell level (Fig. 4g). In this coordinate system, yjr vertex, edge and center represent cells with windows that consist of different CNs. Specifically, if a cell is close to the vertex, its window consists predominately of cells assigned to the CN indicated by that vertex; if a cell is along an edge of the triangle, those cells adjoin the two CNs connected by the edge; if a cell is close to the center of the triangle, its window consists of cells assigned to all three CNs.

Using this coordinate system, we observed that the frequency of tumor edge-to-vessel interface (CN6–CN1 combinations), was positively correlated with tumor pan800 level, indicating favorable drug penetration. In contrast, the frequency of CN6, CN1 and CN9 intermixing was inversely associated with tumor pan800 level, suggesting that increased FAP^+^CD73^+^ CAFs adjacent to vessels may lead to reduced drug entry into tumors (Fig. 4g). Given the association between perivascular FAP^+^CD73^+^ CAFs and reduced drug delivery, we examined whether these CAFs were linked to ECM structures that correlated with reduced antibody transport. We found that the enrichment of ECM2 (periostin, collagen I and fibronectin) was positively correlated with CN9 (FAP^+^CD73^+^ stroma) around endothelial cells (Pearson’s *r* = 0.42, BH-adjusted *P* = 0.0003, *n* = 89 TMA cores from 18 participants; Fig. 4h). This suggests that perivascular FAP^+^CD73^+^ CAFs might contribute to the assembly of ECM2 barriers, restricting antibody diffusion from blood vessels into tumors (Fig. 4i). Similarly, at peritumoral sites, we found that the frequency of the tumor edge to FAP^+^ CAF interface (CN6–CN5 combinations) was inversely correlated with tumor pan800 level (Pearson’s *r* = −0.33, BH-adjusted *P* = 0.015, *n* = 86 TMA cores from 18 participants; Fig. 4j). This suggests that dense layers of FAP^+^ CAFs encompassing tumor nests might shield tumor cells from therapeutic antibody in a subset of participants with HNSCC. Importantly, we found that the enrichment of ECM1 (collagen I and periostin), was positively correlated with CN5 (FAP^+^ stroma) around tumor nests (Pearson’s *r* = 0.33, BH-adjusted *P* = 0.016, n = 85 TMA cores from 18 participants; Fig. 4k), suggesting that FAP^+^ CAFs might contribute to ECM1 formation, thereby reducing antibody penetration into tumors (Fig. 4l). We performed a sensitivity analysis of the spatial-context window size using *k* = 100, 140, 180, 220 and 260 nearest neighbors in HNSCC tissues. Across this range, the frequency of the tumor–FAP^+^ stroma interface consistently exhibited an inverse association with mean tumor pan800 (Pearson *r* ≈ −0.34 to −0.40, *P* < 0.05; Supplementary Fig. 8). Collectively, these data suggest that the spatial organization of FAP^+^ CAFs has a critical role in forming ECM assemblies and impeding drug penetration into tumors (Fig. 4m).

**Extended Data Fig. 7 |:**
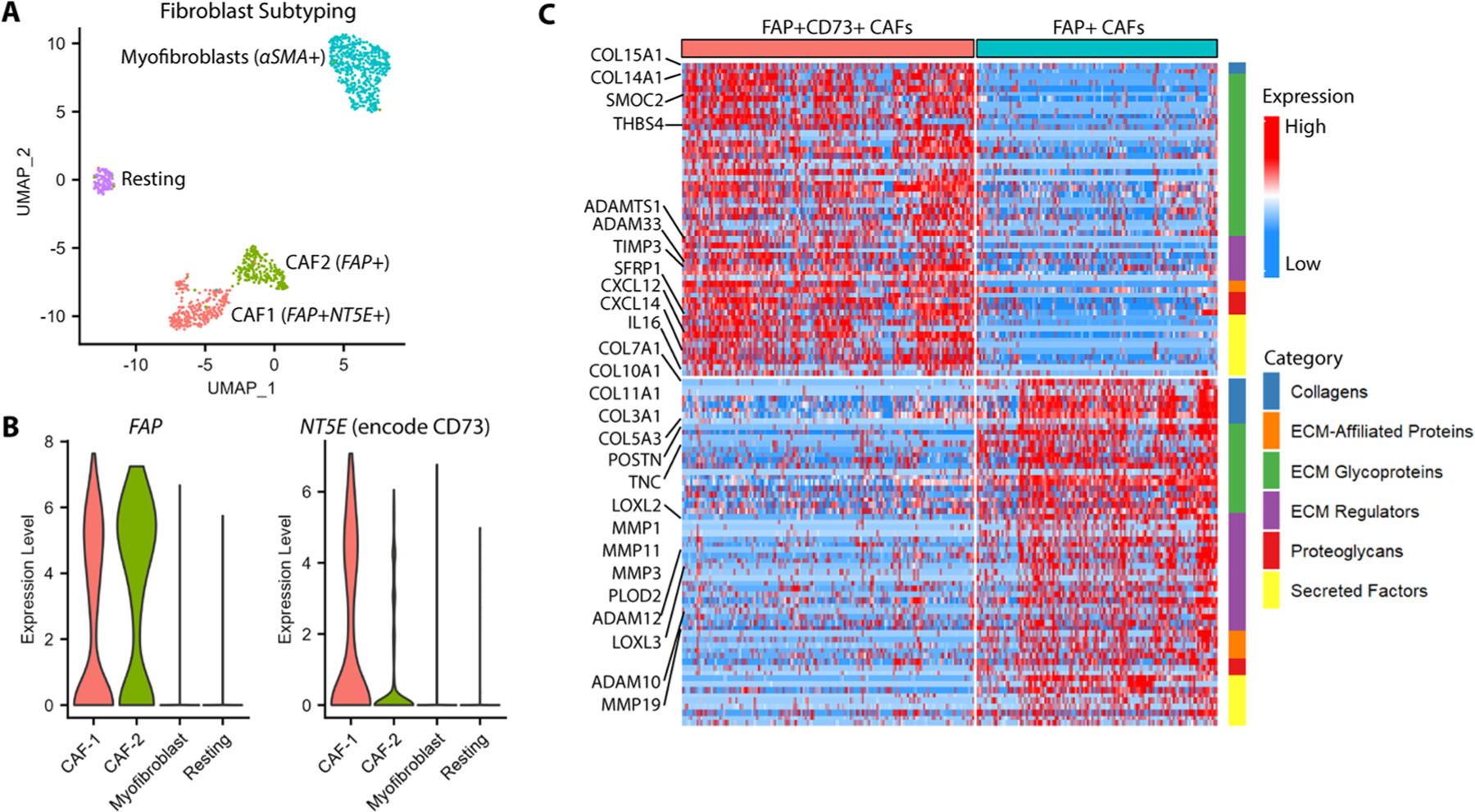
Single-cell RNA-seq analysis for HNSCC tissues. (**a**) UMAP of fibroblast clusters using publicly available scRNA-seq data of HNSCC patient tissue (N = 18 patients). (**b**) Violin plot showing FAP and NT5E (which encodes CD73) expression in the CAF subtypes (N = 18 patients). (**c**) Differentially enriched matrisome genes in CAF subtypes (N = 18 patients).

To further validate these findings at the transcriptomic level, we analyzed a publicly available scRNA-seq dataset of participants with HNSCC^38^ (Extended Data Fig. 7a,b). Consistent with our protein-based CODEX analysis, we identified *FAP*^+^ CAFs and *FAP*^+^*NT5E*^+^ CAFs as the two dominant subtypes. *NT5E* encodes CD73; hereafter, we refer to the CAF subtype as *FAP*^+^*CD73*^+^ for consistency with the CODEX data. Transcriptomic analysis revealed distinct ECM profiles between these CAF subsets (Extended Data Fig. 7c). Compared to the *FAP*^+^*CD73*^+^ CAFs, *FAP*^+^ CAFs expressed higher levels of ECM-associated genes (for example, *POSTN*, *TNC*, *COL3A1*, *COL5A3* and *COL7A1*) and genes encoding ECM-modifying enzymes (for example, *LOXL2*, *PLOD2*, *MMP1*, *MMP3*, *MMP11* and *MMP19*). The upregulation of these ECM-associated genes suggests that *FAP*^+^ CAFs drive ECM synthesis, deposition and remodeling, which may increase ECM density, reduce ECM pore size and create physical barriers to drug transport. *FAP*^+^*CD73*^+^ CAFs exhibited higher expression of genes associated with certain ECM proteins (for example, *COL14A1*, *COL15A1*, *TIMP3* and *ADAMTS1*), inflammation (for example, *C3*, *CXCL12* and *CXCL14*) and angiogenesis (for example, *SFRP1* and *SMOC2*) (Extended Data Fig. 7c), suggesting a distinct role.

We next performed region-based spatial transcriptomic profiling (GeoMx DSP platform) on a subset of participants with HNSCC (*n* = 6 participants). Our spatial transcriptomics data showed that ECM degradation pathways were significantly enriched in participants with HNSCC with high drug delivery (Supplementary Fig. 9a,b), suggesting a more permissive ECM environment in these participants. Additionally, EGFR downregulation pathways were significantly enriched in participants with high drug delivery (Supplementary Fig. 9c), suggesting a potential pharmacological effect of the anti-EGFR antibody panitumumab in these participants.

### Periostin-rich ECM and FAP^+^ CAFs represent a conserved stromal niche associated with reduced antibody delivery in both PDAC and HNSCC

PDAC is a lethal cancer highly resistant to systemic therapy, largely attributed to poor penetration of therapeutic agents through the dense fibrotic stroma surrounding tumor cells. However, multiple mechanisms contribute to therapeutic resistance and measuring antibody delivery in human PDAC has been challenging, limiting our ability to conclusively identify stromal components responsible for therapeutic resistance. Here, we leverage SSP to directly quantify therapeutic antibody delivery and identify specific barriers limiting drug penetration in human PDAC (Supplementary Fig. 10a). Given that HNSCC and PDAC differ markedly in the extent of fibrosis and therapeutic resistance, comparing these tumor types provides a unique opportunity to pinpoint conserved ECM and cellular barriers that restrict intratumoral drug penetration.

**Fig. 5 |:**
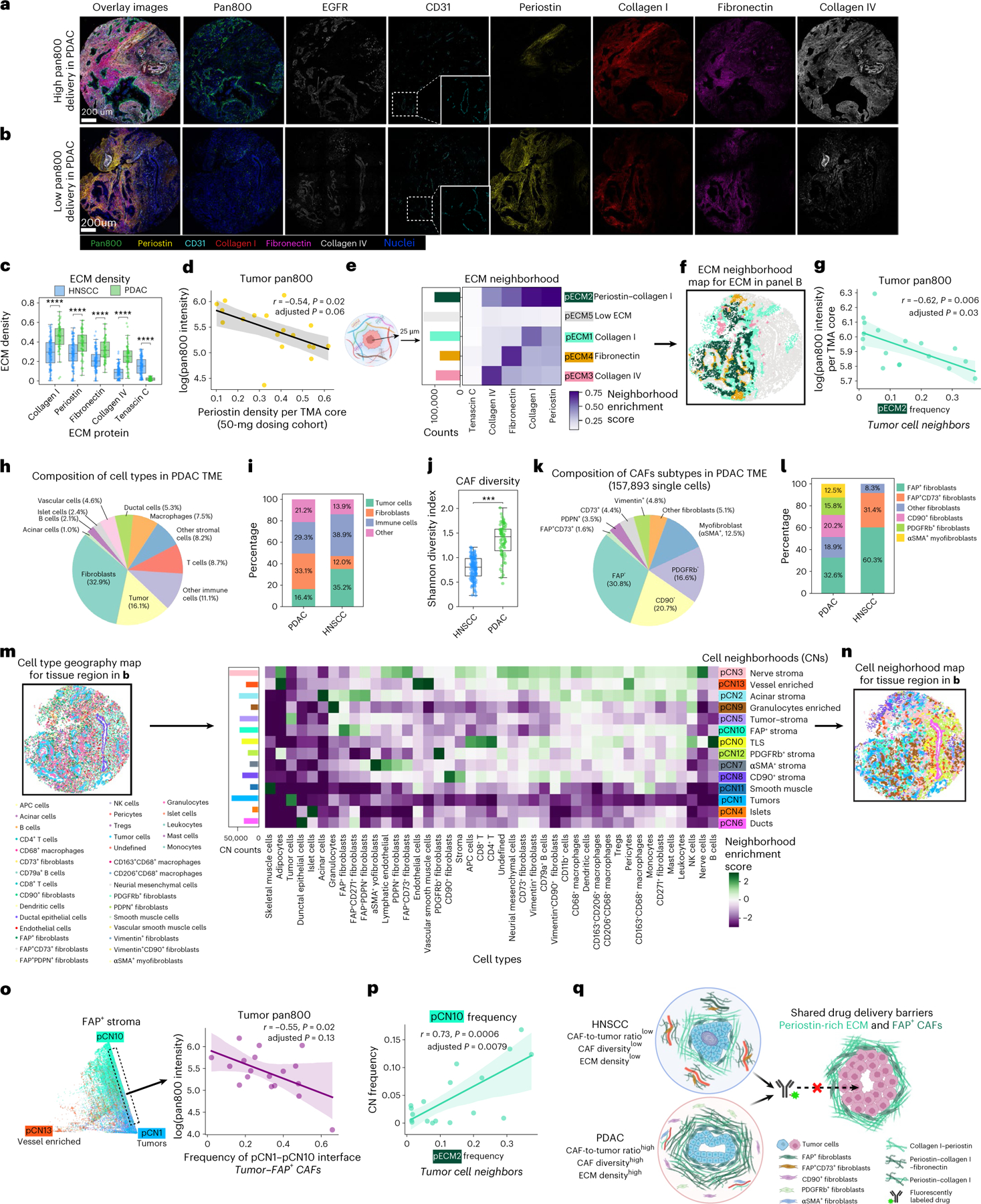
Periostin-rich ECM and FAP^+^ CAF are associated with reduced therapeutic antibody delivery in PDAC. **a**,**b**, Representative overlay and individual images of pan800, CD31, periostin, collagen I, fibronectin and collagen IV in PDAC tissue regions exhibiting high pan800 delivery (**a**) and low pan800 delivery (**b**). **c**, Box plots comparing ECM density across TMA cores from participants with PDAC (65 cores from 12 participants) and HNSCC (111 cores from 18 participants). Each dot represents a TMA core. Statistical comparisons were performed using two-tailed Mann–Whitney *U*-tests with Bonferroni correction: periostin (HNSCC versus PDAC), *P* = 4.04 × 10^−5^; collagen I, *P* = 2.18 × 10^−11^; fibronectin, *P* = 2.18 × 10^−8^; collagen IV, *P* = 3.94 × 10^−17^; tenascin C, *P* = 8.08 × 10^−21^. *P < 0.05, **P < 0.01, ***P < 0.001 and ****P < 0.0001. **d**, Scatter plot showing the association between periostin area fraction and log-transformed tumor pan800 intensity at the region level in PDAC. Each point represents one TMA core (*n* = 18 cores from four participants with PDAC; 50-mg dosing cohort). **e**, Identification of five distinct ECM neighborhoods based on the area fraction of the five ECM proteins evaluated within the 25-μm radius of each anchor cell, defined across PDAC samples (*n* = 12 participants). The cellular illustration was created with BioRender. **f**, Spatial map of ECM neighborhood assignments for the PDAC tissue region shown in **b**. **g**, Scatter plot showing the association between log-transformed tumor pan800 intensity and the frequency of pECM2 neighborhoods in the peritumoral region. Each point represents one TMA core (*n* = 18 cores from four participants with PDAC; 50-mg dosing cohort). **h**, Pie chart showing cell type composition of the TME across *n* = 12 participants with PDAC. **i**, Stacked bar plots comparing major cell type compositions between PDAC (*n* = 12 participants) and HNSCC (*n* = 18 participants) cohorts. **j**, Box plots comparing Shannon diversity index of CAF subtypes between HNSCC and PDAC cohort (*n* = 111 cores from 18 participants with HNSCC, *n* = 65 cores from 12 participants with PDAC). Each dot represents a TMA core. Statistical comparisons were performed using a two-tailed Mann–Whitney *U*-test (*P* = 1.00 × 10^−19^). *P < 0.05, **P < 0.01, ***P < 0.001 and ****P < 0.0001. **k**, Pie chart showing relative abundance of fibroblast subtypes in the TME of PDAC tumors (*n* = 12 participants). **l**, Stacked bar plots comparing fibroblast subtype composition between PDAC and HNSCC cohorts (*n* = 18 participants with HNSCC, *n* = 12 participants with PDAC). **m**, Left, spatial map of cell phenotypes for the PDAC tissue region shown in **b**. Right, a heat map of cell type frequencies in distinct CNs, along with total counts of each CN across the PDAC cohort (*n* = 12 participants). **n**, Spatial map of CN assignments corresponding to the tissue region shown in **m**. **o**, Left, barycentric coordinate projection of spatial windows enriched for pCN1, pCN13 or pCN10. Right, scatter plot showing the association between log-transformed pan800 intensity and the frequency of pCN1–pCN10 interfaces per TMA core (*n* = 18 cores from four participants with PDAC; 50-mg dosing cohort). **p**, Scatter plot showing the association between pCN10 frequency and pECM2 neighborhood frequency in the peritumoral region. Each point represents one TMA core (*n* = 18 cores from four participants with PDAC; 50-mg dosing cohort). **q**, Schematic summary illustrating distinct TME organization in HNSCC and PDAC and shared ECM–CAF spatial barriers to antibody delivery observed across both tumor types. The schematic was created with BioRender. For all scatter plots (**d**,**g**,**o**,**p**), associations were assessed using Pearson’s correlation, with two-tailed *P* values adjusted for multiple comparisons using the BH method. The scatter plots are displayed with linear regression fits and 95% confidence intervals indicated by shaded areas. These analyses are restricted to the 50-mg dosing cohort (*n* = 4 participants, 18 cores) and are presented as supportive evidence of directional concordance with HNSCC rather than standalone statistical proof. Box plots in **c**,**j** show the median (center line), interquartile range (box bounds, 25th–75th percentiles) and whiskers extending to the minimum and maximum values within 1.5× the interquartile range from the lower and upper quartiles, respectively. Data points beyond this range are not shown.

We confirmed that therapeutic antibodies can penetrate and bind to human PDAC tumors, albeit with substantial heterogeneity across participants (representative PDAC regions of two participants in Fig. 5a,b). Differences in vasculature, ECM composition and ECM spatial organization appear to contribute to this variable drug distribution (Fig. 5a,b). Notably, quantitative analysis revealed that collagen I, periostin, fibronectin and collagen IV were significantly more abundant in PDAC than HNSCC across TMA cores (two-tailed Mann–Whitney *U*-test, *P* < 0.0001, *n* = 65 cores from 12 participants with PDAC, *n* = 111 cores from 18 participants with HNSCC; Fig. 5c), suggesting that PDAC tumors harbor more substantial ECM barriers consistent with lower overall drug concentrations in PDAC (Fig. 1d). Similar to HNSCC, periostin and collagen I were the most abundant ECM proteins in PDAC. Notably, periostin was the only one of the five ECM proteins analyzed that exhibited a significant negative correlation with the tumor pan800 level (Pearson’s *r* = −0.54, *P* = 0.02, *n* = 18 TMA cores from four participants with PDAC; Fig. 5d), further implicating it as a barrier to drug penetration across solid tumors. To ensure comparability between HNSCC and PDAC tumors, only participants infused with a 50-mg flat dose of pan800 (*n* = 4) were included in this drug correlation analysis. This restriction was applied to minimize dose-related confounding and to enable direct comparison with the uniformly dosed HNSCC cohort. PDAC analyses are, therefore, presented as supportive evidence of directional concordance rather than standalone statistical proof.

To explore the relationship between ECM composition and drug delivery in PDAC, we analyzed ECM patterns in PDAC and identified five distinct pericellular ECM neighborhoods (Fig. 5e). Specifically, pancreatic ECM1 (or pECM1 to differentiate from HNSCC ECM) was enriched in collagen I, pECM2 was enriched in periostin, and collagen I, pECM3 was enriched in collagen IV, pECM4 was enriched in fibronectin and pECM5 had low density of all markers. These ECM neighborhoods exhibited spatially distinct distributions within PDAC tissues (Fig. 5f). Importantly, at peritumoral sites, enrichment of pECM2 neighborhoods showed a strong negative correlation with tumor pan800 uptake (Pearson’s *r* = −0.62, BH-adjusted *P* = 0.03, *n* = 18 TMA cores from 4 participants; Fig. 5g), suggesting that the combination of periostin and collagen I in pECM2 neighborhoods may pose a notable barrier to drug penetration in PDAC tumors.

To investigate the potential cellular sources of ECM production, we annotated cell types in PDAC TME (Supplementary Fig. 10b–d) and analyzed the cellular composition of human PDAC specimens (Fig. 5h–k). Compared to HNSCC, PDAC displayed a significantly higher fibroblast-to-tumor cell ratio. While fibroblasts in HNSCC were outnumbered by tumor cells (~1:3 ratio), PDAC exhibited a fibroblast-to-tumor ratio of ~2:1 (Fig. 5h,i), which is consistent with the higher ECM abundance in PDAC. Despite greater subtype diversity in PDAC fibroblasts than in HNSCC (two-tailed Mann–Whitney *U*-test, *P* < 0.001, *n* = 65 cores from 12 participants with PDAC, *n* = 111 cores from 18 participants with HNSCC; Fig. 5j), FAP^+^ CAFs are the most abundant fibroblast subtypes in both HNSCC and PDAC (Figs. 4b and 5k). HNSCC CAFs are predominantly composed of two subtypes (FAP^+^ CAFs and FAP^+^CD73^+^ CAFs, which together comprise >90% of all fibroblasts) (Fig. 4b), whereas PDAC fibroblasts included four major subtypes (FAP^+^, CD90^+^, PDGFRβ^+^ and αSMA^+^ CAFs), which together constituted >80% of the fibroblast population (Fig. 5k,l).

To examine the spatial organization of fibroblasts in the PDAC TME, we performed CN analysis. CN identification was evaluated across window sizes of 5–25 nearest cells, with window sizes of 10 providing optimal separation of tumor, vessel-associated and FAP^+^ fibroblast neighborhoods without oversmoothing in the PDAC cohort (Supplementary Fig. 11a). Fourteen distinct CNs were identified (Fig. 5m,n), including four fibroblast-enriched CNs (stroma enriched in FAP^+^, PDGFRb^+^, CD90^+^ and αSMA^+^), a vessel-enriched CN and two tumor CNs. Using CN–CN interaction analysis, we found that the frequency of tumor cell–FAP^+^ CAF interaction (pCN1–pCN10 interface) was inversely correlated with tumor pan800 level (Pearson’s *r* = −0.55, *P* = 0.02, BH-adjusted *P* = 0.13, *n* = 18 TMA cores from four participants; Fig. 5o), consistent with a trend toward reduced antibody access in regions enriched for peritumoral FAP^+^ stroma. We performed a sensitivity analysis of the spatial-context window size in PDAC tissues using *k* = 100, 140, 180, 220 and 260 nearest neighbors. The frequency of the tumor–FAP^+^ stroma interface again showed a consistent inverse association with mean tumor pan800 (Pearson *r* ≈ −0.33 to −0.55; Supplementary Fig. 11b–d). Further analysis revealed a strong positive correlation between the enrichment of pECM2 (periostin and collagen I) and pCN10 (FAP^+^ stroma) around PDAC tumors (Pearson’s *r* = 0.73, BH-adjusted *P* = 0.0079, *n* = 18 TMA cores from four participants; Fig. 5p), suggesting that peritumoral FAP^+^ CAFs may contribute to the formation of dense ECM barriers that constrain antibody transport in PDAC.

Because antibody delivery is highly spatially heterogeneous, driven by local vascular and stromal architecture, region-based analysis is required to capture intratumoral heterogeneity that would be obscured by participant-level averaging. Participant-aware sensitivity analyses indicate that cohort-level correlations reflect heterogeneous participant contributions, with some participants contributing more to the overall correlation magnitude than others (Supplementary Figs. 12–14). For the major drug-delivery associations, leave-one-participant-out analyses attenuated correlation magnitude for certain participant exclusions but preserved effect direction (Supplementary Figs. 12–14).

To validate the spatial associations identified by spatial proteomics, we performed orthogonal single-cell spatial transcriptomic profiling (Supplementary Methods and Supplementary Fig. 15) on a representative subset of the same pan800 clinical trial tissues. Using same-slide pan800 imaging followed by Xenium profiling, we tested two prespecified associations in both tumor types: (1) tumor drug delivery versus periostin (*POSTN*) expression in tumor-proximal *FAP*^+^ CAFs and (ii) tumor drug delivery versus tumor-proximal *FAP*^+^ CAF abundance, quantified as the ratio of neighboring FAP^+^ CAFs to tumor cells. In the HNSCC Xenium cohort (*n* = 10 TMA cores from three participants), tumor pan800 intensity was inversely correlated with *POSTN* expression in tumor-proximal *FAP*^+^ CAFs (*r* = −0.92, BH-adjusted *P* = 0.0002; Supplementary Fig. 15c), whereas the association with tumor-proximal *FAP*^+^ CAF abundance showed a concordant negative trend but did not reach statistical significance (*r* = −0.56, BH-adjusted *P* = 0.09; Supplementary Fig. 15d), likely reflecting limited power in this smaller cohort. In the PDAC Xenium cohort (*n* = 14 TMA cores from three participants), tumor pan800 intensity was inversely correlated with both *POSTN* expression in tumor-proximal *FAP*^+^ CAFs (*r* = −0.80, BH-adjusted *P* = 0.001; Supplementary Fig. 15e) and tumor-proximal FAP^+^ CAF abundance (*r* = −0.55, BH-adjusted *P* = 0.04; Supplementary Fig. 15f). Together, these Xenium analyses provide orthogonal, single-cell spatial transcriptomic validation that *POSTN* expression and tumor-proximal *FAP*^+^ CAF enrichment are reproducibly associated with reduced antibody delivery, with consistent effect direction across tumor types.

Across HNSCC and PDAC, we observed a stromal niche enriched in FAP^+^ CAFs and periostin-rich ECM that was associated with reduced therapeutic antibody delivery (Fig. 5q), supporting a model in which CAF–ECM organization may constrain antibody transport in solid tumors. Although limited in participant number, PDAC tissues exhibited effect sizes and directional trends concordant with those observed in the larger HNSCC cohort, providing supportive evidence for this hypothesis. These findings support the rationale for testing whether modulation of peritumoral FAP^+^ CAFs and periostin-rich ECM can improve antibody delivery in solid tumors.

## Discussion

This study presents an investigation of therapeutic antibody delivery and activity in situ at cellular resolution in human solid tumors. Through SSP analysis of tumor surgical specimens from participants with HNSCC and PDAC, we uncovered marked spatial heterogeneity in drug–target engagement across tumor types, participants, tissue regions and cell types. Importantly, we revealed peritumoral stromal niche of periostin-rich ECM and FAP^+^ CAFs associated with reduced antibody delivery in HNSCC, with directionally consistent trends in PDAC, suggesting a shared delivery-limiting stromal architecture. This study demonstrates in situ single-cell mapping of antibody pharmacology in clinical tumors, directly linking ECM spatial organization, CAF niches and drug exclusion. Conventional methods, such as plasma-based PK and whole-body PET/CT imaging, lack the resolution to capture the heterogeneity of intratumoral drug distribution. SSP overcomes this critical limitation by integrating high-resolution imaging of systemically infused, fluorescently labeled antibodies with multiplexed spatial proteomics, enabling simultaneous measurement of drug distribution, target engagement and TME architecture at unprecedented resolution within intact tissues. This study establishes SSP as a platform for in situ PK and PD analysis at cellular resolution in human tumors, providing a mechanistic framework to understand therapeutic response and advance translational success.

SSP is designed to map spatial constraints on antibody delivery at single-cell resolution rather than to model steady-state therapeutic PK. In this study, subtherapeutic but clinically relevant antibody exposures were used to preserve delivery-dependent gradients and enable visualization of binding-site barriers and stromal transport constraints that are masked at saturating doses. At high antibody doses, target saturation leads to more uniform antibody distribution, reducing spatial contrast among vessels, stroma and tumor binding. Consistent with prior work, the sensitivity of near-infrared fluorescence enables SSP to be applied across a range of clinically relevant dosing conditions, including in combination with unlabeled antibody. Thus, SSP studies can be tailored to address distinct pharmacologic questions and the present work leverages subsaturating exposure to interrogate spatial mechanisms limiting antibody delivery in solid tumors.

Although this study was not designed to assess clinical response, given the window-of-opportunity design, limited treatment duration and subtherapeutic dosing, we observed associations between intratumoral pan800 delivery and molecular PD markers of EGFR downregulation, linking spatial drug delivery to on-target biological activity. Notably, tumor uptake was markedly reduced in PDAC compared to HNSCC, even at exposures approaching clinically relevant levels, highlighting impaired delivery as a potential mechanism of resistance. Beyond panitumumab, the identification of periostin-rich ECM assemblies and FAP^+^ fibroblasts associated with poor delivery suggests broadly relevant barriers that may constrain antibody and antibody–drug conjugate efficacy across tumor types. More broadly, SSP enables direct measurement of drug exposure within heterogeneous tumors, reducing uncertainty in the interpretation of spatial omics data by distinguishing regions that are biologically unresponsive from those that may simply be underexposed to drug.

Given the clinical nature of this study, a nontargeting macromolecule control (for example, labeled IgG) could not be used to distinguish barriers specific to EGFR-targeted antibodies from those that generally limit macromolecule transport. Nevertheless, comeasurement of EGFR expression and pan800 distribution enables separation of target-mediated patterns (that is, tumor nest peripheral binding consistent with a binding-site barrier^23,39^) from nontarget-mediated transport barriers (that is, periostin-rich ECM and FAP^+^ CAF organization), which are considered independent of EGFR binding. Consistent with this interpretation, anti-human Fc staining of endogenous IgG in a representative tumor revealed more diffuse distribution than pan800, supporting target-mediated binding-site barrier effects rather than complete physical exclusion of macromolecules (Supplementary Fig. 16).

This study revealed heterogeneous antibody distribution within both HNSCC and PDAC tumors shaped by shared TME features. In HNSCC, pan800 penetration was highest near the tumor–stromal interface and lowest in central tumor regions, consistent with the binding-site barrier hypothesis^23,39^. Notably, even within EGFR-positive regions, some tumor cells failed to bind pan800, suggesting that antigen expression alone is insufficient to predict drug–target engagement. In this context, EGFR^+^pan800^−^ tumor cells likely represent regions with limited antibody delivery despite target expression, whereas the small EGFR^−^pan800^+^ population reflects low-EGFR tumor cells near the detection threshold with low antibody binding. These findings underscore the need for spatially resolved measurements of drug delivery in human tumors rather than reliance on target expression alone.

In PDAC, drug penetration was more globally suppressed, consistent with its much denser ECM and more diverse CAF populations (Fig. 5). Notably, we also observed high pan800 uptake in myeloid cells across both tumor types, suggesting that myeloid populations may modulate antibody distribution and efficacy. This aligns with prior findings that macrophages can interact with antibody–drug conjugates to release the cytotoxic payload and result in bystander killing^40^ or capture PD1 inhibitors to inhibit their binding to T cells and cause drug resistance^41^. Together, these observations underscore the importance of intratumoral PK/PD studies using SSP to identify the fate of systemically administered antibody therapeutics and disentangle the tumor intrinsic and TME factors influencing drug resistance. Incorporating SSP into early-phase clinical trials could also inform the optimization of biologically effective doses, consistent with the goals of the Food and Drug Administration’s Project Optimus initiative^42,43^.

Our incomplete understanding of drug delivery into solid tumors represents a critical opportunity to elucidate mechanisms underlying therapeutic resistance. A key mechanistic insight from this study is that ECM organization has a critical role in shaping intratumoral antibody distribution across two solid tumors (HNSCC and PDAC) with distinct TME features. While ECM is a well-established regulator of the TME, providing structural support and signaling cues to tumor and stromal cells^44,45^, its impact on drug delivery remains poorly understood. Prior studies have profiled ECM components (the ‘matrisome’) using mass spectrometry of tissue homogenates^46–48^ and preclinical models have shown that total content of collagen and sulfated glycosaminoglycans are inversely correlated with the IgG diffusion^31,32,45,49^. However, these approaches lack spatial resolution. Using SSP, we defined ECM ‘neighborhoods’ that surround individual cells and influence therapeutic access. In HNSCC, periostin-rich ECM neighborhoods were associated with reduced pan800 delivery. In PDAC, which exhibited substantially higher overall ECM density, an even stronger inverse association was observed between tumor pan800 uptake and peritumoral enrichment of periostin-rich ECM. These periostin-rich ECM networks likely impair vascular function, elevate interstitial pressure and form diffusion-limiting barriers to antibody transport. Notably, although prior studies have implicated periostin in cancer progression^50,51^, it was not shown that periostin is as abundant as collagen I in the solid tumor TME and exhibits the strongest inverse correlation with pan800 uptake across tumor types. These findings provide the spatially resolved evidence demonstrating that ECM organization restrict drug delivery in human tumors and identify periostin-rich ECM as a potential barrier to macromolecular drug delivery in solid tumors.

Despite distinct CAFs subtypes and organization in HNSCC and PDAC, our study uncovered a conserved stromal barrier across both tumor types: the enrichment of tumor-proximal FAP^+^ CAFs correlated with peritumoral accumulation of periostin-rich ECM and reduced drug delivery. While previous studies have defined transcriptional subtypes of CAFs through scRNA-seq in HNSCC^38^ and PDAC^50,51^ and implicated their role in ECM regulation, these findings have lacked spatial resolution and linkage to drug delivery. SSP enabled the multiplexed code-tection of CAFs subtypes, ECM composition and therapeutic antibody distribution at the protein level in intact human tissue. In both tumor types, FAP^+^ CAFs were the most abundant CAFs subtype. Xenium analysis demonstrates that tumor-proximal *FAP*^+^ CAFs express high levels of *POSTN* (periostin) in both HNSCC and PDAC, directly linking this CAF population to the periostin-rich ECM assemblies associated with reduced antibody penetration. In HNSCC, peritumoral FAP^+^ CAFs were spatially linked to dense ECM1 neighborhoods and reduced tumor drug uptake, suggesting that FAP^+^ CAFs may contribute to drug exclusion by driving ECM remodeling and reinforcing tumor encapsulation. Although PDAC exhibited greater CAF diversity and denser ECM overall, similar stromal barriers were observed. Peritumoral FAP^+^ CAFs in PDAC colocalized with pECM2 neighborhoods and exhibited an even stronger correlation with reduced tumor drug delivery. Participant-aware analyses highlight heterogeneous participant contributions to cohort-level correlations; consequently, region-level associations are interpreted with attention to participant-to-participant variability in effect magnitude. Together, these data support a model in which FAP^+^ CAFs contribute to periostin-rich ECM assembly and delivery-limiting stromal architecture across tumor types. While preclinical mouse models have described tumor-promoting roles for FAP^+^ CAFs and tumor-restraining function for αSMA^+^ CAFs^50^, our study provides spatially resolved human tissue evidence linking FAP^+^ CAF-associated periostin-rich ECM with reduced therapeutic delivery. These findings support the rationale for combining emerging FAP-targeting therapies, including antibody–drug conjugates^51^, chimeric antigen receptor T cell therapies^52^ and radionuclide theragostics^53^, with antibody therapeutics to improve drug delivery and response. Future studies using genetic or pharmacological perturbations of FAP^+^ CAF and periostin-rich ECM niches will be important to establish causal relationships and guide stromal-targeting strategies.

In summary, SSP provides a generalizable framework for studying intratumoral PK and PD characteristics of therapies and defining drug–target–TME interactions in situ. It is applicable across antibody-based therapeutics, including immune checkpoint inhibitors, antibody–drug conjugates and multispecific antibodies, as well as other macromolecular and cell-based therapies. While implementation currently requires inclusion in a window-of-opportunity trial to capture the intact tumor, SSP offers an unprecedented view of therapeutic distribution and activity within the native human TME, yielding mechanistic insights essential for rational drug development. By integrating SSP into early-phase clinical trials, future studies can refine participant selection, optimize dosing strategies and inform next-generation stromal-targeting therapies, ultimately improving therapeutic outcomes in solid tumors.

This study has two main limitations. First, SSP represents an exploratory, first-in-human, hypothesis-generating approach. As is typical for early-phase clinical molecular imaging trials, participant cohort sizes are modest but complemented by deep, multiplexed analyses and consistent finding across two distinct tumor types. Participant-aware analyses preserved effect direction for the major drug-delivery associations. However, a more precise quantification of interparticipant variability and of participant-level stability is currently constrained by the limited size of the current participant cohort. Future studies with larger cohorts can prospectively test these correlations and deepen our understanding of drug delivery in solid tumors. Although our analyses are correlative and do not establish causality in the absence of direct perturbation, the convergence of same-slide drug imaging, single-cell spatial proteomics and orthogonal single-cell spatial transcriptomics (Xenium) across two tumor types provides robust evidence that tumor-proximal FAP^+^CAF and periostin-rich ECM niches are reproducible delivery-limiting microenvironments in these human tumor cohorts. The spatial maps generated here define a human tissue reference framework for future perturbation studies aimed at testing causality. Second, SSP was demonstrated using an EGFR-targeting antibody administered at subtherapeutic imaging doses to preserve spatial delivery gradients. Extension to other classes of therapeutics will require agent-specific labeling or detection strategies. In particular, analysis of small-molecule drugs would require alternative approaches, such as matrix-assisted laser desorption/ionization mass spectrometry imaging, which are beyond the scope of the present study. Nonetheless, the SSP framework is modular and can be adapted to additional therapeutic modalities as appropriate detection technologies become available.

## Methods

### Human subjects and clinical trial design

For this study, 50 participants who were older than 18 years and who were diagnosed with HNSCC, PDAC or NSCLC were enrolled in three separate phase 1 clinical trials (NCT02415881, NCT03384238 and NCT03582124, respectively) investigating the safety and PK of the EGFR inhibitor pan800 as an optical imaging agent at Stanford Cancer Institute between February 2018 and December 2021. The PET/CT imaging of ^89^Zr-panitumumab in participants with HNSCC was performed in another clinical study (NCT03733210).

All participants were required to have adequate organ function at baseline and received systemic infusion of pan800 followed by surgical resection of their tumors. In these open-label, diagnostic clinical trials, both researchers and participants were aware of trial group assignments (single group assignment). Primary and secondary outcome measures are detailed in the protocols available at ClinicalTrials.gov.

In the phase 1 clinical trial for HNSCC, 29 participants were assigned to receive pan800 at 50-mg dose 1–5 days before surgical removal. Among these 29 participants, 18 participants had FFPE tissue samples available for subsequent fluorescence imaging and CODEX analysis (Supplementary Fig. 1a). In the phase 1 trial for PDAC, 15 participants were assigned to receive pan800 at 25 mg, 50 mg or 75 mg together with 100 mg of unlabeled panitumumab or pan800 alone at a flat 50-mg dose, 2–5 days before curative-intent surgery. Among these 15 participants, 14 participants had tissue samples available for drug quantification in tumor homogenates and 12 participants had FFPE tissue samples available for subsequent fluorescence imaging and CODEX analysis (Supplementary Table 4 and Supplementary Fig. 10a). In the phase 1 study in participants with NSCLC, six participants were assigned to receive pan800 at 25 mg, 50 mg or 75 mg 1–5 days before surgery. Four participants had tissue available for subsequent drug quantification in tumor homogenates.

The trial protocols and the use of participant tissue samples were approved by Stanford’s Institutional Review Board. The trial was conducted in accordance with the Declaration of Helsinki and Good Clinical Practice. All participants provided written informed consent. All participant clinical and biological data used for this study were fully anonymized.

### Drug measurement in fresh tissue specimens and plasma

Fresh surgical biopsy specimens were obtained from eligible clinical trial participants and processed following protocols below^54^. Briefly, participant tissue samples were cut into 25–35-mg pieces and disrupted with a homogenizer in RIPA buffer (50 mM Tris-HCl pH 7.4, 150 mM NaCl, 1% Triton X-100 and 0.1% SDS) containing protease inhibitor tablets (Roche). Next, 100 μl of the tissue homogenates were plated on a 96-well plate and intensity at 800 nm was measured in a near-infrared (NIR) plate reader (Tecan, SparkControl version 2.1). Control tissue samples from untreated participants were similarly weighed, homogenized in RIPA buffer and plated on the 96-well plate with known quantities of pan800 (0–5 mg ml^−1^). A standard curve of pan800 generated from the 800-nm fluorescence intensity of the control tissues was used to determine the quantity of pan800 present in the participant samples. The final concentrations of pan800 were normalized by tissue weight and pan800 dose and reported as the percentage injected dose per kg of tumor tissue.

### Tissue processing and drug imaging

TMAs were constructed from available FFPE tissue blocks of surgical specimens from participants enrolled in the clinical trials and from participants who were not infused with pan800 as control tissues. FFPE tissue slides were annotated to identify tumor areas by a board-certified pathologist (B.A.M. and E.F.). Multiple representative tumor regions from both tumor periphery and interior of each participant with were selected and assembled into TMAs. Tumor periphery and tumor interior were defined on the basis of pathologist-annotated tumor boundaries. For each sample, representative regions immediately adjacent to the tumor boundary were selected as tumor periphery, while regions located toward the geometric center of the tumor mass and separated from the boundary were selected as tumor interior. Each TMA consisted of up to 30 cores (1.5-mm diameter per core) from tumor and adjacent normal participant tissues from the pan800 trials, noninfused control participants and tonsil controls. TMAs were sectioned into 4-μm-thick tissue slices and mounted onto 25-mm glass coverslips (Electron Microscopy Sciences) that were pretreated with VectaBond (Vector Labs). These tissue coverslips were baked at 70 °C for at least 1 h and deparaffinized with xylene and hydrated using graded alcohol series. The coverslips were stained with Hoechst nuclear stain for 10 min and then mounted onto glass slides. These tissue slides were scanned in an Olympus VS200 slide scanner (VS200 ASW 3.3, build 24382) using DAPI and NIR wavelength channels and a ×40 oil lens (numerical aperture (NA) = 1.35) to visualize the spatial distribution of pan800.

### CODEX antibody panel construction

Commercially available, purified, carrier-free anti-human antibodies were conjugated to previously validated maleimide-modified short DNA oligonucleotides (Supplementary Tables 2 and 3)^27,55^. Following previously published protocols^56^, conjugations were performed at a 2:1 w/w ratio of the oligonucleotide to antibody with 50 μg or 100 μg of antibody per reaction. All conjugated antibodies were titrated on the tissue of interest and validated in CODEX multicycle experiments^27^. Briefly, appropriate FFPE tissue slides were stained with the conjugated antibody at a starting dilution of 1:100 and titrated according to signal-to-noise ratio using ATTO 550 and Alexa Fluor 647 as fluorescent reporters. The exposure times, fluorophores and optimal cycles of all the antibodies were optimized on the specific TMAs through CODEX multicycle experiments. The specificity, sensitivity and reproducibility of oligonucleotide-conjugated antibody staining have previously been demonstrated across multiplex experiments in healthy and diseased tissues^27,28,55–58^.

### CODEX tissue staining and imaging

After NIR microscopic drug imaging, the tissue coverslips were gently removed from the glass slides and the CODEX antibody staining and imaging were performed on the same tissue coverslips. Briefly, heat-induced epitope retrieval (HIER) was performed using Dako target retrieval solution (pH 9; Agilent, S236784–2) at 97 °C for 10 min, followed by 30 min of cooling to room temperature. Coverslips were washed for 10 min in 1× Tris-buffered saline immunohistochemistry (IHC) wash buffer with Tween-20 (Cell Marque). Afterward, a water-repellent barrier was drawn around the tissue region using a Bondic pen. Nonspecific binding was blocked for 1 h at room temperature using 100 μl of FFPE blocking buffer (S2 buffer containing B1 (1:20), B2 (1:20), B3 (1:20) and BC4 (1:15)). The coverslip was next stained with a cocktail of all CODEX antibodies (Supplementary Table 2) at a volume of 100 μl overnight at 4 °C in a sealed humidity chamber on a shaker. Following overnight staining, coverslips were washed in S4 buffer for 4 min, then fixed in S4 containing 1.6% paraformaldehyde solution for 10 min and washed with 1× PBS for 1 min. The coverslip was then incubated in ice-cold methanol for 5 min and washed with 1× PBS for 1 min. The final fixative solution (BS3) at a volume of 100 μl was added to the tissue and incubated for 20 min at room temperature, followed by 1 min in 1× PBS. The coverslip was then transferred to S4 solution and stored at 4 °C. The recipes for all the CODEX experimental buffers were previously detailed in a protocol paper^56^.

Before CODEX multicycle imaging, each coverslip was mounted onto a custom-made acrylic plate. The multicycle image acquisitions were conducted using Akoya’s CODEX microfluidics instrument connected to a Keyence BZ-X710 microscope configured with four fluorescent channels (DAPI, FITC, Cy3 and Cy5) and a ×20 Nikon objective (NA = 0.75). A 3 × 3 tiled acquisition with nine *z* planes was used for HNSCC TMAs. In the last cycle of the CODEX runs, nuclei were stained with DRAQ5 and imaged by the Cy5 channel. Hematoxylin and eosin staining and brightfield imaging were performed immediately after completion of the CODEX multicycle experiment on the same Keyence microscope.

### Opal multiplex immunofluorescence (mIF) staining and imaging

Following the NIR imaging of pan800, we detached the coverslip from the tissue slide and carried out a four-color multiplex fluorescence staining of the investigated HNSCC TMAs using antibodies to the following antigens: pan-cytokeratin (CK) (AE1/AE3, dilution: 1:550), EGFR (EP38Y, dilution: 1:100) and pEGFR (Tyr1173, dilution: 1:40). For fluorescence-labeling, we used an Opal Polaris seven-color manual IHC detection kit according to the manufacturer’s instructions^59^. After cutting 3-μm-thick sections with high-precision microtomes, specimens were incubated at 60 °C for 1 h and deparaffinized in a descending alcohol series. Pretreatment for mIF was carried out using antigen-demasking buffers specific for the chosen antigen in each staining round. The subsequent staining process was performed three times in a serial fashion. Sections were incubated with the antibody diluent for 10 min at room temperature, followed by incubation with the primary antibody either for 60 min at 29 °C. After applying Opal polymer horseradish peroxidase (HRP)-conjugated secondary antibody and Opal fluorophore solution each for 10 min, antibodies were removed by microwave treatment (HIER) before the next round of staining. Finally, the nuclei were counterstained with DAPI and after rinsing with PBS, samples were covered with a coverslip using ProLong gold mounting medium (Invitrogen, P36930). The antibodies, their dilutions, the according retrieval buffers and the sequence of usage are described in Supplementary Table 5. The four-color Opal slides were visualized using the PhenoImager Fusion (Akoya Biosciences).

### Antibody screening, validation and titration for Opal mIF staining

The antibodies applied for mIF were first screened and validated using uniplex IF stains HNSCC tissue and tonsil tissue followed by orthogonal validation using conventional DAB IHC. All validations were performed under the supervision of a dermatopathologist and a board-certified pathologist (M.H. and C.K.) and collated with known expression patterns published online (The Human Protein Atlas and Pathology Outlines), as well as the published literature.

After chromogen-based IHC was used for all targets (pEGFR, PanCK and EGFR), uniplex IF was conducted to optimize the antibody titrations, to generate spectral libraries required for mIF analysis, determine the antibody–OPAL–dye groups used for mIF and their optimal staining sequence. Briefly, after deparaffinization and fixation, 3-μm tissue sections were processed with retrieval buffers for 15 min in a microwave oven. Similar to mIF, sections were then incubated with the protein block followed by incubation with the primary antibody for 60 min at 29 °C and, after the application of the Opal polymer HRP secondary antibody and Opal fluorophore solution each for 10 min, slides were washed and counterstained with DAPI. To determine the optimal staining sequence for mIF, each uniplex IF was conducted twice with various HIER. In particular, for each antibody, we stained three uniplex IF with 1× and 5× heat pretreatments. Similar to IHC staining, the correct titration of the single antibodies in uniplex IF stains were chosen carefully to obtain a uniform, specific and correct staining pattern.

### CODEX image preprocessing and registration

The raw imaging data of pan800 acquired from Olympus slide scanner were converted from vsi format into ome-tiff using QuPath software. The raw CODEX imaging data were processed using a GPU-accelerated parallelized image processor named RAPID^22^. This method involves the following steps: (1) three-dimensional image deconvolution to reduce off-focus light; (2) selection of the best focus plane from the *z* stacks; (3) image stitching of individual tiles to one montage per cycle per wavelength channel per TMA core; (4) drift compensation to co-register the montages across imaging cycles; (5) subtraction of images acquired from a blank cycle (tissue autofluorescence background) from all the other imaging cycles; and (6) generation of a hyperstack consisting of all the wavelength channels across all imaging cycles per TMA spot.

After preprocessing, the two imaging datasets were coregistered using a customized method that involved the following steps. Firstly, the nuclear images of the two imaging data were extracted and min–max-normalized with extreme values removed. Secondly, phase correlation^60^ was used to estimate the initial geometric transformation. ‘Similarity’ transformation was used to take into account translation, rotation and isotropic scaling between the two nuclear images. Thirdly, deformable registration was applied to the initial registration result from step 2 to refine the transformation for nuclear images performed. This was performed using the diffeomorphic demons registration method^61,62^. Lastly, the same geometric transformations for nuclear images were applied to NIR drug images.

### Cell segmentation

Whole-cell-based segmentation was performed using Mesmer^21^, a deep-neural-network-based approach pretrained on a dataset named TissueNet that consists of about 1,000,000 manually labeled cells. A cytoplasmic image was generated by summing multiple epithelial markers (for example, PanCK, CK19, EGFR and Na^+^K^+^ATPase) and pan-lymphocyte marker CD45. The pretrained Mesmer model was used to segment the whole cells from the cytoplasmic images and the nuclei from the nuclear images. Both the whole-cell mask and the nuclear mask were generated and then matched for each cell on the basis of the maximum overlap.

### CODEX protein and drug quantification

For cell-surface markers, the protein expression was computed by dividing the total marker intensity of each cell by the cell size measured from whole-cell masks. In addition, for cell-surface markers, signal spillover compensation was performed by multiplying an inverse adjacency matrix with the cell-surface protein marker expression intensity. For nuclear markers (for example, Ki67, FoxP3 and p53), the protein expression was measured from the nuclear intensity normalized by the nuclear size of each cell measured from nuclear masks. Drug binding per cell was defined as the total pan800 intensity of each cell normalized by the cell size. The total EGFR expression and levels of pan800 binding in tumor cells from participants with HNSCC were gated by customized intensity thresholds determined by EGFR^−^pan800^−^ cells (that is, T cells and B cells). The gating results were also mapped back onto images and compared to the actual EGFR staining and pan800 fluorescence images to verify the gating results.

### Cell type identification

First, nucleated cells were identified and artifacts were removed by gating on Hoechst^+^DRAQ5^+^ cells using customized intensity threshold for each coverslip. Next, the protein expression of all the cells was *z*-normalized within each TMA^63^. Subsequently, the normalized protein expression of nucleated cells from all the participants was used for unsupervised Leiden clustering to identify distinct cell clusters. Initially, 42 antibody markers (CD45, CD8, CD4, CD3, CD15, FoxP3, CD25, PD1, CD16, CD14, CD79a, CD20, CD11c, CD11b, CD68, CD163, CD206, tryptase, HLA-DR, CD74, granzyme B, CD57, CD56, synaptophysin, CD31, CD34, PDPN, PDGFRb, vimentin, CD90, FAP, CD73, αSMA, PanCK, CK19, EGFR, maspin, CD271, Na^+^K^+^ATPase, CAIX, Ki67 and P53) were used to cluster the data into 58 clusters. Each cluster was manually assigned a cell type on the basis of combinations of canonical protein markers. Next, clustering was refined through an iterative process involving merging similar clusters, separating distinct clusters, manual annotation and visual validation using CODEX images. Finally, a total of 31 fine cell types and 15 coarse cell types were identified from the tissues from participants with HNSCC. To further identify subtypes of tumor cells, three markers (PDPN, Ki67 and CAIX) from all the tumor cells across participants with HNSCC were used for Leiden clustering, which identified nine tumor clusters. Similar clusters were merged and mixed clusters were further subclustered, resulting in four final tumor clusters (PDPN^+^ tumors, Ki67^+^ tumors, CAIX^+^ tumors and PDPN^−^CAIX^−^Ki67^−^ tumors). A similar cell type identification approach was conducted to annotate cell types in CODEX images of PDAC tissues. The Shannon diversity index^64^ was calculated to compare the CAF subtype diversity in HNSCC and PDAC.

### Tumor nest segmentation

In this study, a tumor nest was defined as a microanatomical region consisting of spatially connected tumor cells. To segment the tumor nest region, a combination of four membrane and cytoplasmic markers (PanCK, CK19, EGFR and Na^+^K^+^ATPase) was applied using a customized image segmentation method. First, all four markers were min–max-normalized and summed to generate one membrane image per TMA core. Next, the membrane images were smoothed with edges preserved by directional coherence enhancing diffusion^65^ and image contrast was adjusted using Matlab’s ‘imadjust’ function (Matlab R2024a). Next, the initial tumor nest regions were segmented using customized thresholds and combined with tumor cell segmentation masks to detect and remove areas that did not contain tumor cells. Lastly, the tumor nest segmentation masks were refined by multiple steps of morphological processing (image dilation, closing, filling holes and image opening).

### ECM neighborhood identification

Five ECM protein marker images (collagen I, collagen IV, fibronectin, tenascin C and periostin) were included in this analysis. Positive ECM staining areas were segmented using a two-step method. First, each ECM image was smoothed with edge preserved by directional coherence enhancing diffusion^65^. Next, the smoothed ECM images were binarized by Otsu’s image thresholding using the Matlab R2024a function ‘multithresh’. Following image segmentation, a circular window with a radius of 25 μm surrounding each cell was extracted and the area fraction of each ECM marker was computed by normalizing the number of positive ECM pixels by the total noncellular areas within each window for each ECM images. Each circular window of each center cell was converted into a vector of length 5, which was subsequently clustered using the *k*-means clustering algorithm implemented in Python’s scikit-learn as ‘MiniBatchKMeans’ with *k* = 4 for HNSCC and *k* = 5 for PDAC.

### Cell neighborhood identification

To identify CNs, each TMA core was scanned by a moving window consisting of a center cell and its nearest neighboring cells^27^. The cell type frequencies in each window were used as the input to a *k*-means clustering algorithm using Python’s scikit-learn implementation of MiniBatchKMeans. To determine the optimal window size (including the center cell), window sizes in the range of 5–25 were compared by comparing the histogram of cell type composition and frequencies and by evaluating the corresponding CN spatial image maps. A window size of 9 and a cluster number of 10 were selected to identify ten distinct CNs in HNSCC. A window size of 10 and a cluster number of 14 were selected to identify 14 distinct CNs in PDAC.

### Barycentric coordinate projection

Windows containing the 180 nearest spatially neighboring cells around a given index cell were extracted and frequencies of CN assignments to each individual cell in each window were computed^37^. In HNSCC, those windows where CN1, CN5 and CN6 or CN1, CN6 and CN9 comprised more than 70% of the cells were selected and projected linearly under the linear map sending the unit coordinate vectors to a point of an equilateral triangle. In PDAC, those windows where pCN1, pCN10 and pCN13 comprised more than 65% of the cells were selected. The projected points were colored by the CN assignment at the center of the window. The frequencies of the CNs assigned to cells corresponding to different vertices, edges and intermixing of three CNs mapped to a barycentric plot were computed and normalized by the total number of cells assigned to the corresponding three CNs per TME core. The Pearson’s correlation coefficients were calculated between the normalized CN frequencies and the tumor pan800 intensity of the same TMA core across all tissue samples.

### Opal multiplexed image processing and analysis

We first coregistered pan800 drug imaging with Opal multiplexed imaging of the same tissue using the shared nuclear image and a custom image registration method described above. We used previously developed tools to identify and classify the cells in each image. For cell identification, we used Mesmer^21^, a pretrained deep learning algorithm for automated, accurate cell segmentation using DAPI, PanCK and EGFR for whole-cell segmentation. Subsequently, we filtered out debris, background-subtracted pan800 signals and *z*-normalized all investigated markers (PanCK, EGFR, pEGFR and pan800) across the investigated TMAs. We used the resulting data for Leiden-based clustering to identify stroma cells and tumor cells^66^. The resulting annotated dataset was used to conduct CN analysis^27^. On the basis of the *k* = 15 nearest neighbors, we detected three main neighborhoods that reflected the three major tissue compartments (bulk tumor, tumor stroma boundary and stroma). We quantified the mean log-normalized expression of pEGFR and pan800 across these major tissue compartments. We compared the mean pEGFR expression and mean log-normalized pan800 expression within each of the unique tissue cores and conducted a Pearson correlation analysis to assess the relationship of pEGFR and pan800 expression within the HNSCC TME.

### scRNA-seq dataset analysis

We obtained preprocessed scRNA-seq count data from tumors from participants with HNSCC^38^ and extracted fibroblasts, tumor cells and endothelial cells on the basis of the annotations of the cells made by the authors. To clean the data, we excluded doublet cells and dying cells. Dying cells were identified by high levels of mitochondrial genes. Cells were labeled as doublets if they had expression of either immunoglobulin genes or T cell genes (CD3, CD8A and CD8B). Tumor cells, endothelial cells and fibroblasts from Puram et al. were extracted into three separate Seurat objects. Cells from lymph node samples were excluded from downstream analysis. The Seurat objects were reclustered using SCTransformed count data at resolution of 0.8 for fibroblasts. We identified five clusters for the fibroblast population with one cluster coming entirely from sample HNSCC24. The cells from this cluster were removed and the remaining cells were reclustered. To characterize the ECM programs of various cell types, we first identified differentially expressed genes (adjusted *P* value < 0.05) using Seurat (minimum expression percentage = 0.25, log fold change threshold = 0.25 and test.use = MAST). We further annotated ECM-associated genes by cross-referencing the matrisome genes previously defined^46^. The results were visualized using ComplexHeatmap package in R.

### Spatial transcriptomics

Spatial transcriptomic data were acquired by the Nanostring DSP platform (GeoMx DSP version 2.5.1145) using the whole-transcriptome atlas^67,68^. Briefly, a 5-μm FFPE section of HNSCC TMA was deparaffinized and stained overnight with antibodies targeting cancer cells (PanCK), endothelial cells (CD31) and CAFs (FAP). The section was hybridized with the RNA probes before being loaded into the instrument. A total of 24 regions of interest including four cell categories (PanCK^+^, CD31^+^, FAP^+^ and PanCK^−^CD31^−^FAP^−^) were selected for collection and library preparation. Sample processing and sequencing were performed by the Technology Access Program at NanoString. Probe measurements and quality control data were provided by NanoString.

### Differential gene expression analysis

The standR package^69^ was used for processing the raw counts. Regions of interest with a nucleus count < 50 or library size < 50,000 were excluded from the analysis. We identified 300 negative control genes across participants using standR, which were then used as input for Remove Unwanted Variation 4 (RUV 4) to account for batch effects. Principal component analysis and relative log expression plots were then assessed to see whether batch correction was successful and whether technical variation because of library size was accounted for. Differential expression was performed using the limma-voom pipeline^70^. In our analysis, it was of interest to see which genes were differentially expressed in different segment markers on the basis of the concentration of drug; hence, a design matrix was set up with drug concentration information (participant-level information) as the fixed effect in the model. The model matrix used matrices generated from RUV 4 as covariates to account for interparticipant variability. The statistics were performed at the participant level for each of the three markers (PanCK, FAP and CD31). Each participant (with multiple regions) was grouped into high versus low drug delivery. The ‘duplicate correlation’ function from limma was used to account for dependency of regions from the same participant (intraparticipant variability). Genes with low coverage in the dataset were also removed to allow a more accurate mean–variance relationship and reduce the number of statistical tests. A moderated *t*-test was used to compare genes between our contrast conditions and a corrected *P*-value cutoff of 0.05 was used to assess significant genes that were upregulated or downregulated using the BH method.

### Gene set enrichment analysis

We used a preranked GSEA approach^71^ to uncover functional pathways associated with the differentially expressed genes in our dataset. Genes were ranked on the basis of their log fold change values derived from the differential expression analysis and the clusterProfiler package (version 4.4) was used to conduct the enrichment analysis. To obtain comprehensive gene sets representing various biological processes and canonical pathways, we sourced curated gene sets from the Molecular Signatures Database (MSigDB) and Reactome database. To determine the statistical significance of the enrichment scores, we performed 1,000 permutations and applied the false discovery rate (FDR) correction to account for multiple testing. Gene sets with an FDR-adjusted *P* value below 0.05 were considered as significantly enriched. The interpretation of the enriched gene sets was guided by their biological functions, allowing us to gain valuable insights into the underlying mechanisms.

### Statistics

A total of 50 participants were enrolled across three phase 1 clinical trials involving HNSCC, PDAC and NSCLC. Bulk tumor drug quantification from fresh tissue homogenates was available for 25 participants with HNSCC, 14 participants with PDAC and four participants with NSCLC. High-resolution spatial analyses required intact FFPE tissue and successful CODEX profiling; accordingly, the spatial proteomics cohort comprised 18 participants with HNSCC and 12 participants with PDAC. Region-level analyses were performed on TMA cores selected to capture intratumoral heterogeneity, yielding variable numbers of analyzable regions depending on the specific biological question and predefined inclusion criteria (for example, presence of tumors, vasculature, specific CNs and/or ECM neighborhoods). For PDAC drug–ECM correlation analyses, we further restricted analyses to the uniformly dosed 50-mg cohort (*n* = 4 participants) to minimize dose-related confounding and evaluate cross-cancer consistency of the biological relationships observed in HNSCC.

Multiple statistical methods were applied. A two-tailed *P* value < 0.05 was considered statistically significant, unless otherwise specified. Significance levels are indicated as follows: *P < 0.05, **P < 0.01, ***P < 0.001, ****P < 0.0001 and NS (not significant; *P* > 0.05). Region-level analyses were performed using tissue regions (TMA cores) as the unit of analysis to capture intratumoral heterogeneity in antibody delivery. Because multiple regions may originate from the same participant, region-level analyses do not assume statistical independence at the participant level. Accordingly, region-level associations were evaluated using Pearson’s correlation with two-sided *P* values, with BH correction applied where appropriate, and were interpreted in conjunction with participant-aware robustness analyses. These included participant-colored visualizations, leave-one-participant-out sensitivity analyses, participant-aggregated analyses and regression models accounting for participant-level clustering, which together assess preservation of effect direction and heterogeneity in effect magnitude across participants. Continuous association analyses were conducted using Pearson’s correlation coefficient (*r*) with corresponding two-sided *P* values, shown alongside linear regression plots (seaborn. lmplot), with 95% confidence intervals displayed as shaded areas. Group comparisons in box plots were performed using the two-tailed Mann–Whitney *U*-test. Box plots display the median and interquartile range, with box limits representing the 25th and 75th percentiles. Clinical participant data were summarized using descriptive statistics. Python 3.7.16 with key libraries including scanpy 1.9.3, matplotlib 3.5.3, scipy 1.7.3, scikit-learn 1.0.2 and seaborn 0.11.2 was used for data and statistical analysis.

## Extended Data

## Supplementary Material

Supplementary Material

**Supplementary information** The online version contains supplementary material available at https://doi.org/10.1038/s41587-026-03152-x.

## Data Availability

Data supporting the findings of this study are available in the paper, its Supplementary Information and public repositories. The external HNSCC scRNA-seq dataset used for validation is available from the Gene Expression Omnibus (GEO) under accession number GSE103322. NanoString GeoMx digital spatial profiling data generated in this study are available from the GEO under accession number GSE249198. Source data are provided with this paper.

## Code availability

Code for processing CODEX multiplexed imaging data (https://github.com/nolanlab/RAPID), cell segmentation (https://github.com/van-valenlab/deepcell-tf), CN analysis (https://github.com/nolanlab/NeighborhoodCoordination), tissue schematics (https://github.com/nolanlab/TissueSchematics) and ECM neighborhood analysis (https://github.com/GuolanLu/ECM_neighborhoods) is available from GitHub.
