# Supplementary material for: Single-cell spatial pharmacobiology identifies conserved stromal barriers to therapeutic antibody delivery in human solid tumors
Source: Nat Biotechnol. Author manuscript; Available in PMC 2026 Jul 22. (PMC13390730; doi:10.1038/s41587-026-03152-x)
Supplement: Supplementary Material [file NIHMS2196166-supplement-Supplementary_Material.pdf]

Contents

Supplementary Methods ... 2
- **Evaluation of image registration accuracy** ... 2
- **Single-cell spatial transcriptomics (Xenium) for orthogonal validation.** ... 3

Supplementary Tables ... 4
- **Supplementary Table 1** ... 4
- **Supplementary Table 4** ... 5
- **Supplementary Table 5** ... 6

Supplementary Figures ... 7
- **Supplementary Figure 1** ... 7
- **Supplementary Figure 2** ... 9
- **Supplementary Figure 3** ... 11
- **Supplementary Figure 4** ... 12
- **Supplementary Figure 5** ... 14
- **Supplementary Figure 6** ... 15
- **Supplementary Figure 7** ... 16
- **Supplementary Figure 8** ... 18
- **Supplementary Figure 9** ... 19
- **Supplementary Figure 10** ... 20
- **Supplementary Figure 11** ... 21
- **Supplementary Figure 12** ... 23
- **Supplementary Figure 13** ... 24
- **Supplementary Figure 14** ... 26
- **Supplementary Figure 15** ... 28
- **Supplementary Figure 16** ... 30

Supplementary Methods

Evaluation of image registration accuracy

To assess the accuracy of co-registration between pan800 fluorescence images and CODEX multiplexed images, we performed quantitative evaluation on nuclear images acquired from the same FFPE tissue section at two stages of the imaging workflow. Specifically, nuclei were imaged during pan800 fluorescence scanning on an Olympus VS200 slide scanner (DAPI channel acquired concurrently with pan800) and subsequently imaged during each cycle of CODEX multiplexed imaging on an Akoya/Keyence platform (DAPI/DRAQ5 channel). Although both nuclear datasets originate from the same physical tissue section, they are acquired on different microscopy platforms and at different processing stages, following deparaffinization, antigen retrieval, antibody staining, and multiple fluidic cycles. These factors introduce differences in optical configuration, image contrast, background characteristics, point-spread function, and potential local tissue deformation. We therefore prioritized geometry-based evaluation metrics that are robust to inter-platform photometric variability and minor processing-induced distortions.

We used Target Registration Error (TRE) [1] as the primary metric to quantify spatial alignment accuracy at single-cell resolution. TRE provides an intensity-independent measure of geometric correspondence and is well suited for evaluating registration accuracy across imaging platforms. Nuclei were segmented independently in the target image (TI) and the registered image (RI) using Cellpose[2] with identical parameters for both datasets. For each nucleus in the TI, the nearest nuclear centroid in the RI was identified, and the Euclidean distance between the paired centroids was defined as the TRE for that nucleus. This procedure was applied across all evaluated tissue regions (n = 111). TRE values were summarized as mean ± standard deviation. Across all regions, the mean TRE was 0.97 ± 0.41 µm, indicating subcellular registration accuracy. This displacement is small relative to a typical nuclear diameter of approximately 10–12 µm, demonstrating that the registration pipeline achieves high spatial fidelity despite sequential tissue processing and inter-platform imaging differences.

As a complementary metric, we evaluated registration accuracy using the Dice Similarity Coefficient (DSC) on paired nuclear segmentation masks. The standard DSC was computed as:

$$\mathrm{DSC} = \frac{|\mathrm{RI} \cap \mathrm{TI}| + |\mathrm{TI} \cap \mathrm{RI}|}{|\mathrm{TI}| + |\mathrm{RI}|}$$

where RI and TI denote the nuclear masks in the registered and target images, respectively. Using this standard definition, the mean DSC across tissue regions was 0.76 ± 0.05. This value reflects partial overlap between nuclear masks and is sensitive to small discrepancies in nuclear boundaries, which can arise from intensity and contrast differences between imaging systems rather than from true spatial misregistration. To account for biologically reasonable uncertainty in nuclear boundary definition, we implemented a refined DSC metric by dilating each nuclear mask by one pixel (approximately 0.4 µm), defining dilated masks. The refined DSC was calculated as:

$$\mathrm{Refined\_DSC} = \frac{|\mathrm{RI} \cap \mathrm{Dilate\_TI}| + |\mathrm{TI} \cap \mathrm{Dilate\_RI}|}{|\mathrm{TI}| + |\mathrm{RI}|}$$

Given typical nuclear dimensions, this one-pixel tolerance represents a conservative allowance for boundary variability while preserving sensitivity to true misalignment. Under this refined definition, the mean DSC increased to 0.92 ± 0.05, indicating strong cell-level correspondence between registered images.

Together, TRE and refined DSC analyses demonstrate that the phase-correlation followed by diffeomorphic demons registration pipeline achieves high spatial accuracy at the single-cell level, despite potential tissue distortions introduced by tissue processing and sequential imaging. The discrepancy between standard and refined DSC values primarily reflects minor differences in nuclear boundary delineation attributable to inter-platform imaging characteristics rather than genuine registration error.

Single-cell spatial transcriptomics (Xenium) for orthogonal validation.

Tissue processing and data acquisition. Single-cell spatial transcriptomics (Xenium, 10x Genomics) was used as an orthogonal modality to assess the relationship between FAP^{+}CAFs, periostin, and antibody delivery. Tissue microarray (TMA) cores from a representative subset of the same HNSCC and PDAC cohorts analyzed by CODEX and pan800 imaging were selected to fit within a single Xenium slide imaging area (10 mm × 20 mm). Prior to Xenium processing, FFPE slides were deparaffinized, nuclei were stained, and near-infrared microscopy was performed using the same pan800 imaging parameters to quantify tumor drug delivery. Following drug imaging, coverslips were removed and commercial Xenium Prime protocols were applied on the same tissue sections to measure 5,106 transcripts (Xenium Prime 5K Human Pan Tissue & Pathways Panel, and a custom gene set of ECM- and fibroblast-associated genes, Xenium onboard analysis software version: xenium-3.3.1.1).

Data processing and cell annotation. The overall transcriptomic quality was high, with adjusted negative control codeword rate at 0.0%, adjusted negative control probe rate at 0.1%, and adjusted genomic control probe rate at 1.2%. During quality check, TMA cores that were damaged or lost during tissue processing or had no tumor cells left were removed, and high-quality Xenium data were obtained from 10 HNSCC ($n = 3$ patients) and 14 PDAC cores ($n = 3$ patients) for further analysis. Cells with fewer than 40 transcripts or fewer than 15 detected genes were also excluded. Data were normalized using counts per 10,000 (CP10K) followed by log1p transformation. Highly variable genes (top 3,000) were selected for downstream analysis. Dimensionality reduction was performed using principal component analysis (50 components), followed by neighborhood graph construction (30 nearest neighbors) and Leiden clustering. Cell clusters were annotated based on canonical marker gene expression, with FAP^{+} CAFs confirmed by expression of FAP.

Correlation analysis. Tumor-proximal FAP^{+} CAFs were defined as FAP-expressing fibroblasts located within 40 µm of tumor cells. Sensitivity analyses using 30 µm and 50 µm thresholds yielded similar correlations and did not change conclusions. For each TMA core, we quantified (i) the mean log1p-transformed POSTN expression in tumor-proximal FAP^{+} CAFs and (ii) the mean pan800 fluorescence intensity across tumor cells. Pearson's correlation was used to assess associations between tumor drug delivery and (i) POSTN expression in tumor-proximal FAP^{+} CAFs and (ii) tumor-proximal FAP^{+} CAF abundance, with two-tailed p values adjusted for multiple comparisons using the Benjamini–Hochberg method.

Supplementary Tables

Supplementary Table 1. Clinicopathological characteristics of the HNSCC cohort.

Number of HNSCC patients	18
Age at study inclusion (Range)	60 (42-76)
Infusion dose of panitumumab-IRDye800	50mg flat dose
Gender	
- Male	11 (61.1%)
- Female	7 (38.9%)
Primary tumor location	
- Oral	15 (83.3%)
- Maxilla	2 (11.1%)
- Neck	1 (5.6%)
Mean tumor size (mm, 95% CI)	43.0 (33.6-52.4)
T stage	
- T2	4 (22.2%)
- T3	5 (27.8%)
- T4a	8 (44.4%)
- T4b	1 (5.6%)
N stage	
- N0	12 (66.7%)
- N2b	2 (11.1%)
- N3b	4 (22.2%)
AJCC stage	
- II	3 (16.7%)
- III	3 (16.7%)
- IVA	8 (44.4%)
- IVB	4 (22.2%)

Supplementary Table 4. Clinicopathological characteristics of the PDAC cohort.

Number of PDAC patients	12
Age at study inclusion (Range)	68 (40-83)
Gender	
- Male	8 (66.7%)
- Female	4 (33.3%)
Primary tumor location	
- Head	7 (58.3%)
- Tail	3 (25%)
- Uncinate process	1 (8.3%)
- Ampulla	1 (8.3%)
Mean tumor size (mm, range)	31.7 (13.0-52.0)
T stage	
- T1c	2 (16.7%)
- T2	5 (41.7%)
- T3	3 (25.0%)
- T3b	1 (8.3%)
- T4	1 (8.3%)
N stage	
- N0	3 (25.0%)
- N1	4 (33.3%)
- N2	5 (41.7%)
Dosing cohort	
- 25 mg panitumumab-IRDye800 + 100mg panitumumab	1 (8.3%)
- 50 mg panitumumab-IRDye800 + 100mg panitumumab	4 (33.3%)
- 75 mg panitumumab-IRDye800 + 100mg panitumumab	3 (25.0%)
- 50 mg panitumumab-IRDye800	4 (33.3%)

Supplementary Table 5. Resource table for Opal multiplexed fluorescence imaging

Reagent or Resource	Manufacturer	Identifier
**Chemicals and Reagents**		
Xylenes	FisherScientific	X54S24S24
99.8 % EtOH	Carl Roth GmbH	K928.3
Tris-HCl (Pufferan) p.a.	Carl Roth GmbH	9090.3
NaCl (99,8%)	Carl Roth GmbH	9265.1
Formalin solution (neutral buffered 10 %)	Sigma-Aldrich	HT501128-4L
Fluorescence Mounting Medium	Agilent DAKO	S302380-2
OPAL Polaris 7-Color (manual Kit)	Akoya Biosciences	NEL861001KT
Opal Polymer HRP (Ms+Rb)	Akoya Biosciences	ARH1001EA
Opal 690	Akoya Biosciences	-
Opal 620	Akoya Biosciences	-
Opal 780	Akoya Biosciences	-
TSA-Dig	Akoya Biosciences	-
DAPI	Akoya Biosciences	FP1490
Antibody Diluent	Akoya Biosciences	ARD1001EA
Antibody Diluent	DAKO	S0809
Amplification Diluent	Akoya Biosciences	IF1498
AR9 Buffer	Akoya Biosciences	AR900250ML
Eukitt (mounting medium)	Avantor delivered by VWR	03989-100 ml
PBS (without Ca2+, Mg2+) (10X)	gibco	14200-075
tri-Natriumcitrate (Dihydrat)	Carl Roth GmbH	3580.1
Dual endogenous enzyme block	DAKO	S2003
Hematoxylin	Agilent (DAKO)	CS700
ImmPRESS HRP Horse Anti-Mouse IgG (Kit)	Vector Laboratories	MP-7402
ImmPRESS HRP Horse Anti-Rabbit IgG (Kit)	Vector Laboratories	MP-7401
ImmPACT_DAB Substrate, HRP	Vector Laboratories	SK-4105
Antibodies and Proteins		
Pan-cytokeratin [AE1/AE3]	Biolegend	914204
EGFR [EP38Y]	abcam	ab174481
pEGFR [Tyr1173]	Invitrogen	44-794G
**Critical Commercial Instruments, Consumables, Kits and Assays**		
PhenoImager Fusion	Akoya Biosciences	
ST4020 small linear stainer	Leica	14050946425
Precisio Compact Oven with Aluminum Chamber and Analog Display	VWR	97018-372
Dye box for 10 slides	Carl-Roth GmbH	H552.1
Dye box and inserts (PMP)	Carl-Roth GmbH	2290.2 / 2291.2
LTS filter tips, 10 µl	Rainin	30389225
LTS filter tips, 200 µl	Rainin	30389239
LTS filter tips, 1000 µl	Rainin	30389212
Cellstar Tubes (15ml)	Greiner Bio-one	188271
Reaction tubes 1,5ml	Greiner Bio-one	618201
Microscope slides ThermoScientific (Superfrost Plus)	Menzel GmbH	1800AMNZ

Supplementary Figures

Supplementary Figure 1. **Data analysis for HNSCC patients**.

(A) The CONSORT diagram of the HNSCC clinical study.

(B) Dot plot of protein marker expression for each cell type at a higher granularity. Results depict about 1,000,000 cells analyzed in surgical specimens from patients with HNSCC.

(C-E) Cell density distribution by marker expressions for three major fibroblast subtypes.

(F-G) High reproducibility for identifying representative fibroblast subsets (a new set of annotations for 1,000 representative fibroblasts in each fibroblast subtype was compared against the initial annotations to calculate the recall and precision).

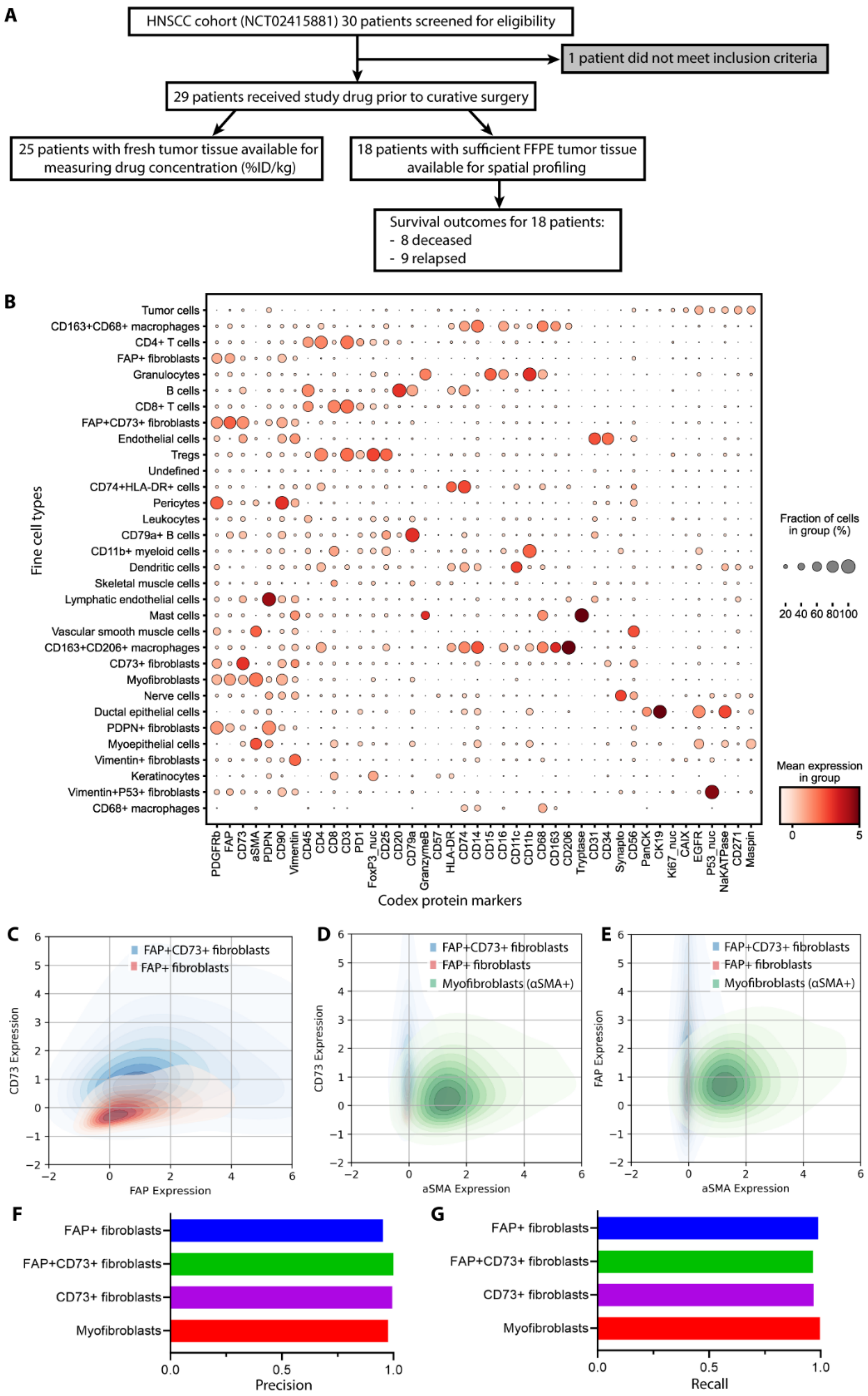
A
HNSCC cohort (NCT02415881) 30 patients screened for eligibility
1 patient did not meet inclusion criteria
29 patients received study drug prior to curative surgery
25 patients with fresh tumor tissue available for measuring drug concentration (%ID/kg)
18 patients with sufficient FFPE tumor tissue available for spatial profiling
Survival outcomes for 18 patients:
- 8 deceased
- 9 relapsed
B
Fine cell types
Codex protein markers
Fraction of cells in group (%)
20 40 60 80 100
Mean expression in group
0 5
C
FAP+CD73+ fibroblasts
FAP+ fibroblasts
CD73 Expression
FAP Expression
D
FAP+CD73+ fibroblasts
FAP+ fibroblasts
Myofibroblasts (αSMA+)
CD73 Expression
aSMA Expression
E
FAP Expression
aSMA Expression
F
FAP+ fibroblasts
FAP+CD73+ fibroblasts
CD73+ fibroblasts
Myofibroblasts
Precision
G
Recall

Supplementary Figure 2. EGFR and pan800 thresholding.

(A) Sensitivity analysis of EGFR and pan800 thresholding. The fraction of $EGFR^+$/$pan800^+$ tumor cells was recalculated after applying ±10% shifts to the original EGFR and pan800 intensity thresholds. Across these perturbations, the $EGFR^+$/$pan800^+$ population varied by less than 2%, demonstrating robustness of quadrant proportions to reasonable threshold variation.

(B-D) Representative examples of EGFR- and pan800-gated tumor cell maps. Representative tumor regions illustrating tumor cell classification based on EGFR and pan800 fluorescence intensity. Magenta arrows indicate $EGFR^-$/$pan800^+$ tumor cells, and cyan arrows indicate $EGFR^+$/$pan800^+$ tumor cells. B) Tumor cell maps showing EGFR/pan800-based gating results derived from CODEX and pan800 imaging. C) EGFR fluorescence imaging (CODEX) performed on the same tissue section shown in (B). D) EGFR IHC staining performed on adjacent serial tissue sections corresponding to the regions shown in (B), confirming low but detectable EGFR expression in $EGFR^-$/$pan800^+$ tumor cells.

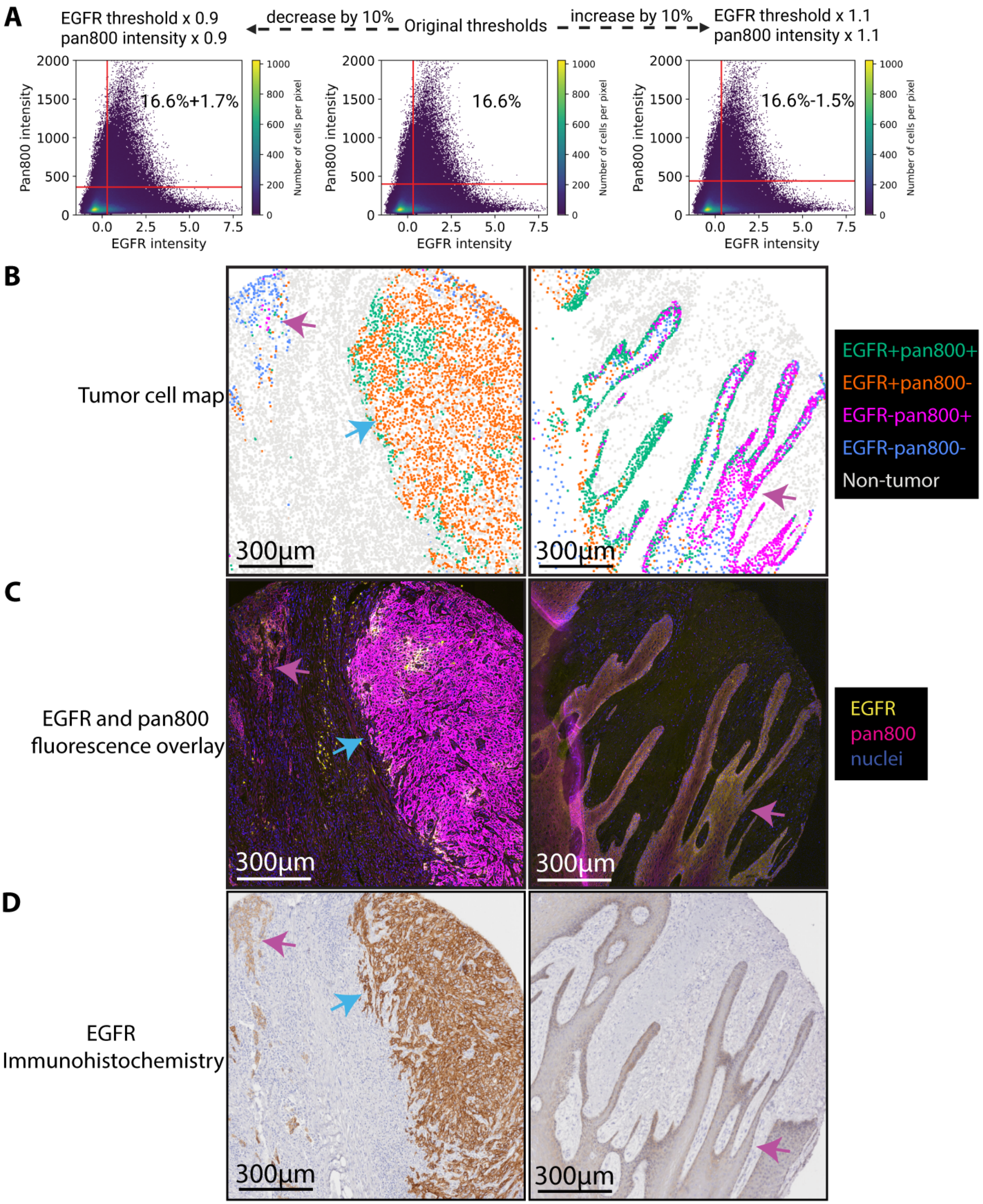
A
EGFR threshold x 0.9
pan800 intensity x 0.9
decrease by 10%
Original thresholds
increase by 10%
EGFR threshold x 1.1
pan800 intensity x 1.1
16.6%+1.7%
16.6%
16.6%-1.5%
Pan800 intensity
EGFR intensity
Number of cells per pixel
B
Tumor cell map
EGFR+pan800+
EGFR+pan800-
EGFR-pan800+
EGFR-pan800-
Non-tumor
C
EGFR and pan800
fluorescence overlay
EGFR
pan800
nuclei
D
EGFR
Immunohistochemistry
300µm

Supplementary Figure 3. **ECM analysis for HNSCC**.

(A) Representative examples of ECM segmentation masks corresponding to ECM images shown in Figure 3A.

(B) Box plots of the area fractions of five ECM proteins by patients (18 patients). Box plots show median and IQR. Statistical comparisons were performed using two-tailed Mann–Whitney U test. ns = not significant; * $p < 0.05$, ** $p < 0.01$; *** $p < 0.001$, **** $p < 0.0001$.

(C) Representative overlay images showing periostin and collagen I in relation to tumor regions and $CD31^{+}$ vasculature. Magenta arrows indicate preferential enrichment of periostin at tumor–stroma boundaries, whereas collagen I is more broadly distributed within the surrounding stroma. Cyan arrows highlight peritumoral blood vessels that are embedded within periostin-rich ECM.

(D-E) ECM neighborhood compositions in D) primary tumors and E) metastatic lymph nodes (N = 18 HNSCC patients).

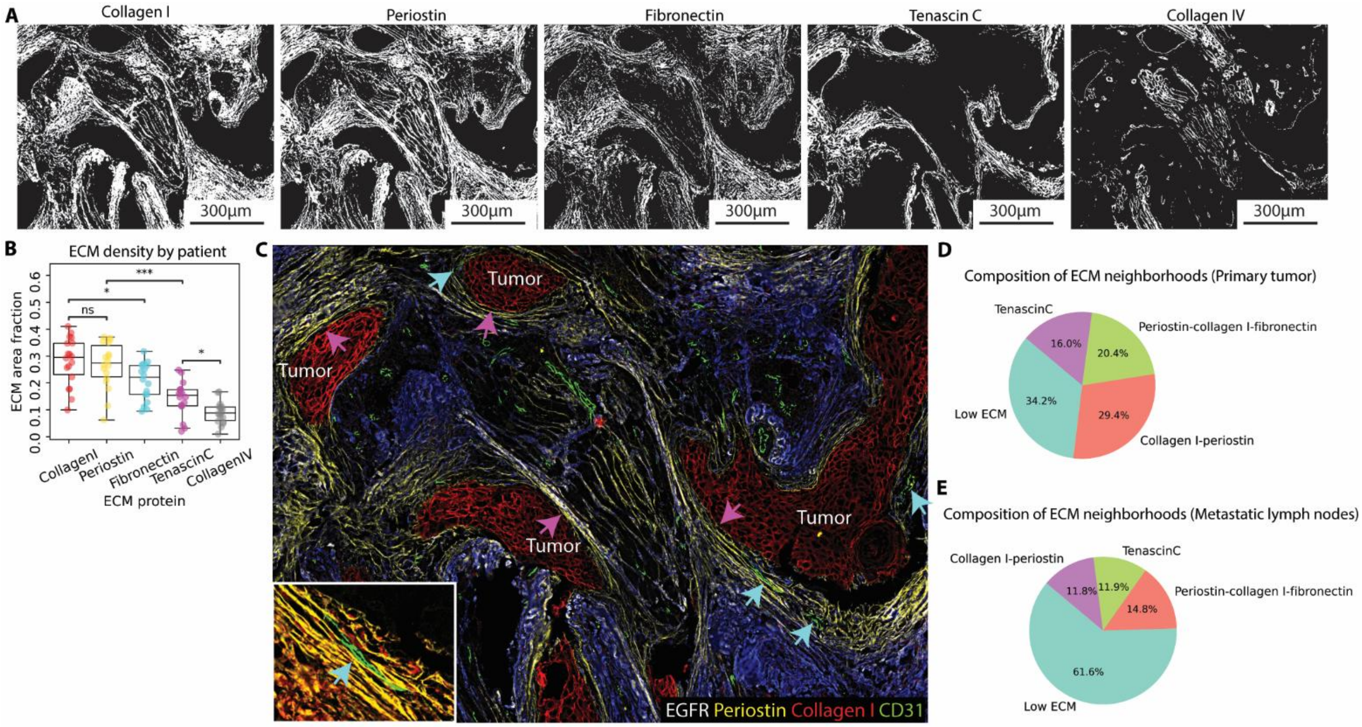

Supplementary Figure 4. Robustness of ECM neighborhood clustering to cluster number selection and across spatial scales.

(A) Adjusted Rand Index values comparing ECM neighborhood clustering results for k = 4, 5, and 6, indicating moderate-to-substantial concordance across resolutions.

(B–D) ECM neighborhood clustering heatmaps for k = 4, 5, and 6, showing preservation of dominant ECM-low and periostin–enriched neighborhoods as clustering granularity increases.

(E) Sankey diagram illustrating correspondence and continuity of ECM neighborhoods across k = 4, 5, and 6, demonstrating stability of major ECM structures across clustering resolutions.

(F-I) ECM neighborhood clustering heatmaps and ECM2–pan800 correlations computed using neighborhood radii of 12.5 µm, 25 µm, 50 µm, and 100 µm. Across all radii, ECM2 frequency remains inversely associated with pan800 intensity, with maximal correlation observed at 25 µm (Pearson's r = -0.34, two-sided p = 0.0012). Associations were assessed using Pearson's correlation, with two-tailed p values. Shaded area indicates 95 confidence interval of regression curve.

A Adjusted Rand Index (ARI) values comparing ECM neighborhood clusterings across k = 4, 5, and 6.

Comparison	ARI
k=4 vs k=5	0.587
k=4 vs k=6	0.673
k=5 vs k=6	0.629

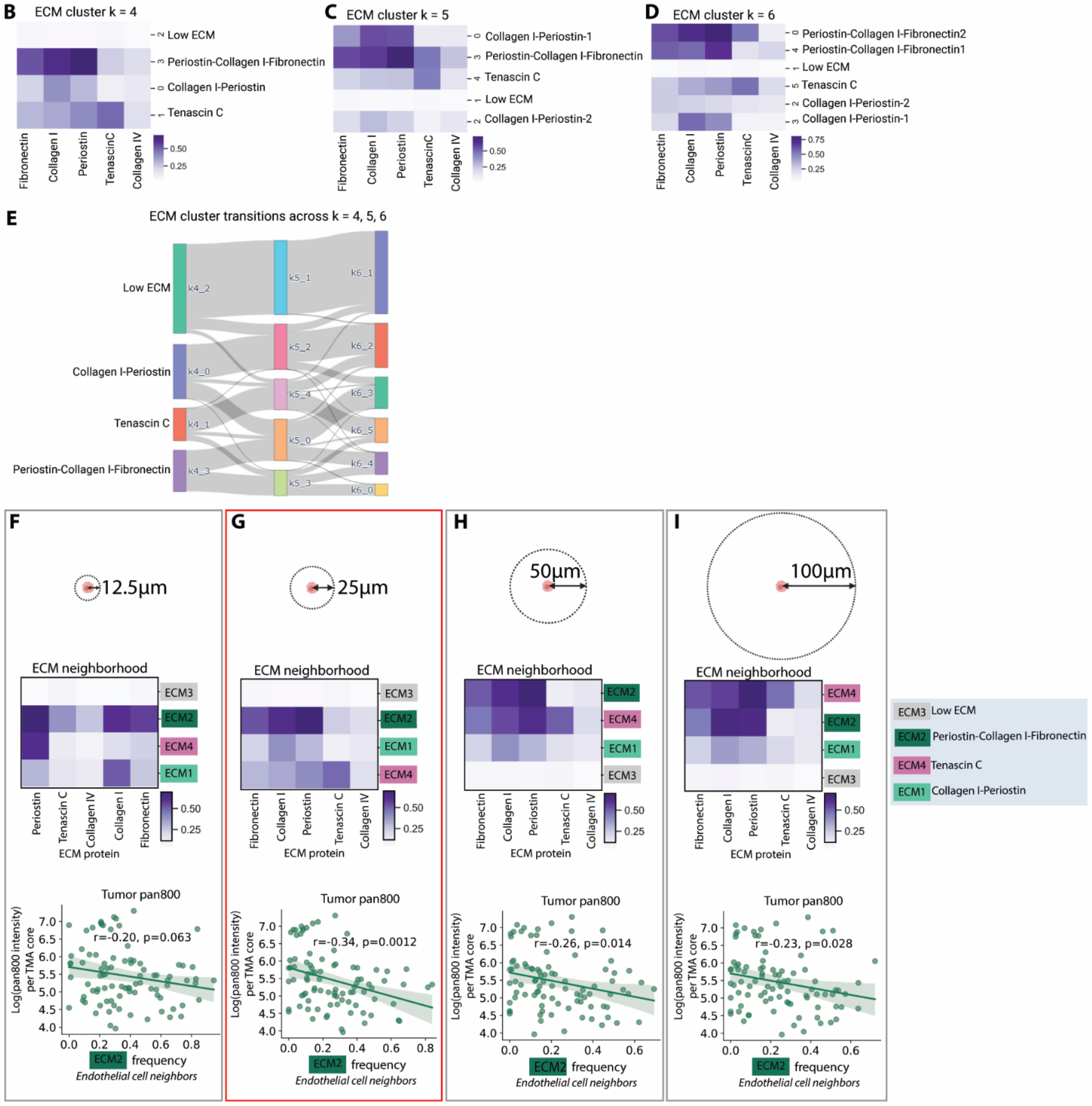

Supplementary Figure 5. Quantification of tumor-adjacent vessels and periostin-rich ECM.

(A) Schematic illustrating the quantification method. Blood vessels ($CD31^+$) were identified within a tumor-adjacent zone extending 100 µm outward from the tumor boundary. Tumor-adjacent vessel area fraction was calculated as the total vessel area within this zone, normalized to the zone area. Periostin density was quantified within a 50-µm neighborhood surrounding each tumor-adjacent vessel, restricted to non-cellular ECM space. Panel A was created with BioRender.com.

(B) Scatter plot showing the inverse correlation between vessel-associated periostin density and tumor-adjacent vessel area fraction across primary HNSCC tissue regions (n = 102 TMA cores). Pearson correlation coefficient (r) and two-sided p value are shown (Pearson $r = -0.385$, $p < 0.01$).

(C–D) Representative image overlays of pan800, periostin, and CD31 in regions with high tumor drug delivery (C) and low tumor drug delivery (D). In regions with high drug delivery, tumor-adjacent vessels display open, rounded lumens and are surrounded by periostin-low ECM. In regions with low drug delivery, tumor-adjacent vessels exhibit reduced luminal area and are embedded within periostin-rich ECM.

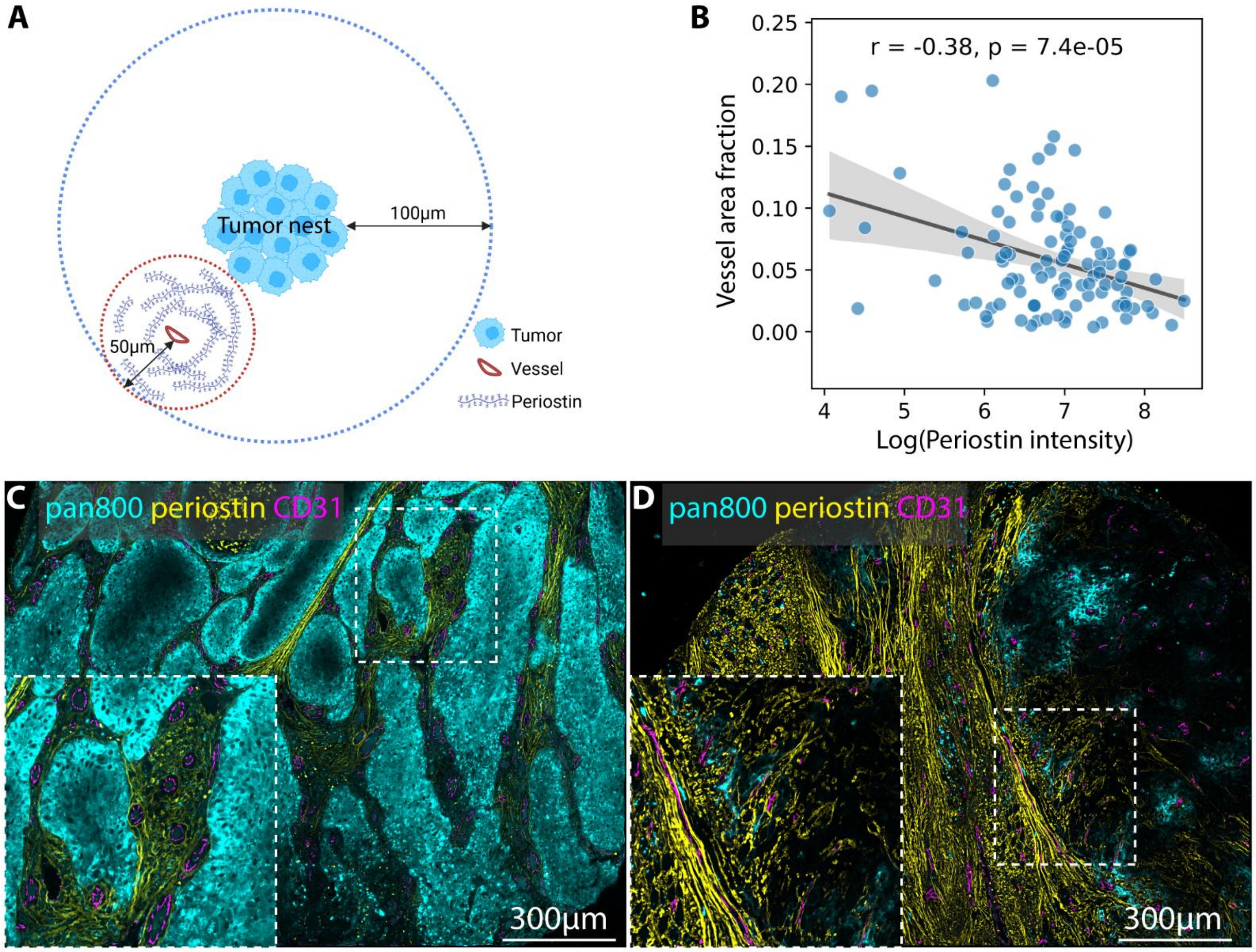

Supplementary Figure 6. Evaluation of batch effects across TMAs and coverslips.

(A) UMAP of all cells colored by TMA ID (equivalent to coverslip ID) and by cell type, showing no segregation driven by TMA.

(B) Expression of CAIX, Ki67, and PDPN mapped onto the UMAP, demonstrating that the only partial separation corresponds to a biologically distinct hypoxic tumor subtype rather than a technical batch effect.

(C) Per-core scatter plots colored by TMA ID showing relationships between log-transformed tumor pan800 intensity versus frequency of ECM2 in the neighborhood of endothelial cells (corresponding to Figure 3H).

(D) Per-core scatter plots colored by TMA ID showing relationships between log-transformed tumor pan800 intensity versus ECM1 frequency around tumor cells (corresponding to Figure 3J).

Cores from different TMAs are interspersed, indicating minimal batch effects after per-TMA Z-normalization. Each point represents one TMA core. Pearson correlation coefficient (r) and two-sided p value are shown. Shaded area indicates 95 confidence interval of regression curve.

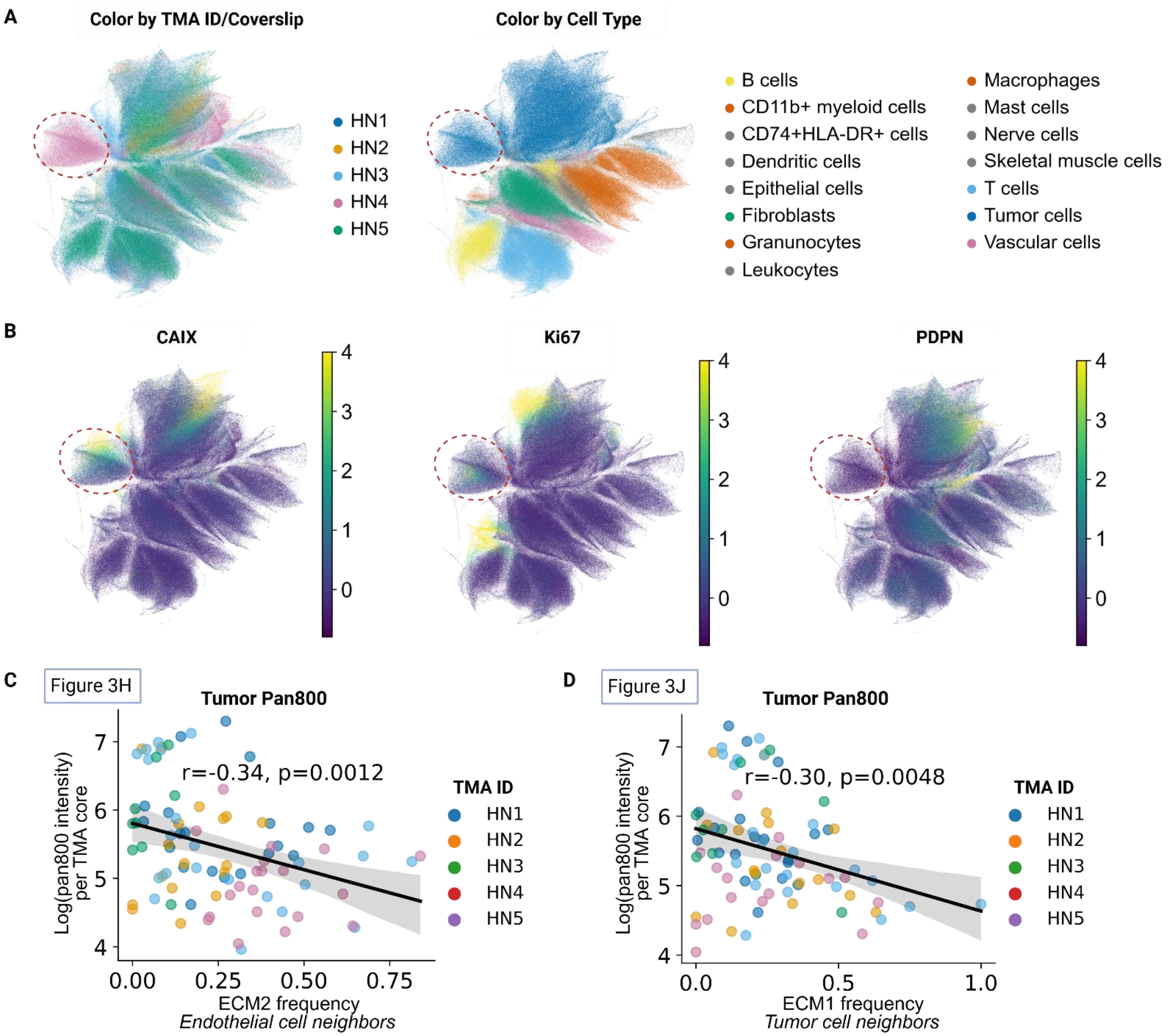

Supplementary Figure 7. Cell neighborhood (CN) analysis in HNSCC.

(A) CN analysis across window sizes ranging from 5 to 25 nearest cells in HNSCC. Red dotted line outlined vascular cells, including endothelial cells, pericytes, and vascular smooth muscle cells. Black dotted line outlined tumor cells. Orange dotted line outlined FAP+ fibroblasts and blue dotted line outlined FAP+CD73+ fibroblasts. A window size of 9 nearest cells provided the best biological granularity, clearly separating tumor-enriched, vessel-associated, and FAP^+ fibroblast neighborhoods while avoiding over-smoothing observed at larger window sizes. This window size was therefore used for downstream HNSCC CN analyses.

(B-C) CN compositions of HNSCC B) primary tumors (N=18 patients) and C) lymph node metastasis (N=4 patients).

(D) Scatter plot of tumor pan800 uptake versus frequency of CN1 surrounding tumor edges per TMA core (N = 59 cores). Pearson’s r, with two-tailed p value was shown. Shaded area indicates 95 confidence interval of regression curve.

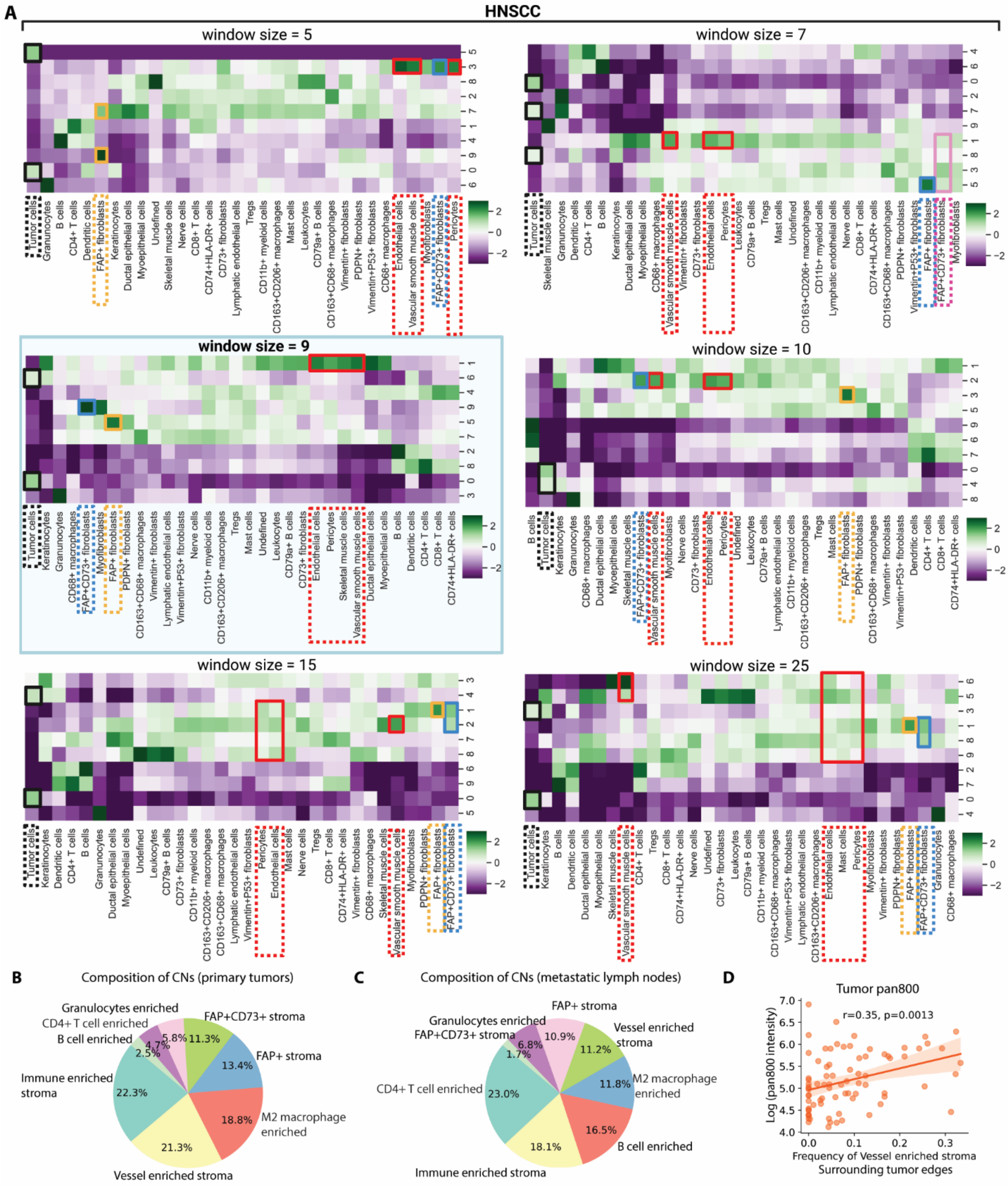

A
HNSCC
window size = 5
window size = 7
window size = 9
window size = 10
window size = 15
window size = 25
B
Composition of CNs (primary tumors)
Granulocytes enriched
CD4+ T cell enriched
B cell enriched
FAP+CD73+ stroma
FAP+ stroma
Immune enriched stroma
M2 macrophage enriched
Vessel enriched stroma
2.5%
4.7%
5.8%
11.3%
13.4%
22.3%
18.8%
21.3%
C
Composition of CNs (metastatic lymph nodes)
Granulocytes enriched
FAP+CD73+ stroma
FAP+ stroma
Vessel enriched stroma
M2 macrophage enriched
CD4+ T cell enriched
B cell enriched
Immune enriched stroma
1.7%
6.8%
10.9%
11.2%
11.8%
23.0%
16.5%
18.1%
D
Tumor pan800
r=0.35, p=0.0013
Log (pan800 intensity)
Frequency of Vessel enriched stroma Surrounding tumor edges

Supplementary Figure 8. Sensitivity analysis of spatial-context (SC) window size in HNSCC.

(A) Barycentric spatial-context maps generated using k-nearest-neighbor windows with k = 100, 140, 180, 220, and 260 in HNSCC tissues. Each point represents a local window summarized by the composition of three cellular neighborhoods (CNs) and projected into barycentric space.

(B) Association between the frequency of the tumor–FAP^{+} stroma interface and mean tumor pan800 across window sizes. Pearson correlation coefficients are shown for each k, demonstrating a consistent inverse association across window-size choices (nominal two-sided p values).

(C) Agreement of SC label assignments across window sizes, quantified by the adjusted Rand index (ARI) relative to the reference window size k = 180. SC labels show highest agreement at intermediate window sizes (k = 140–220), with reduced agreement at smaller and larger windows.

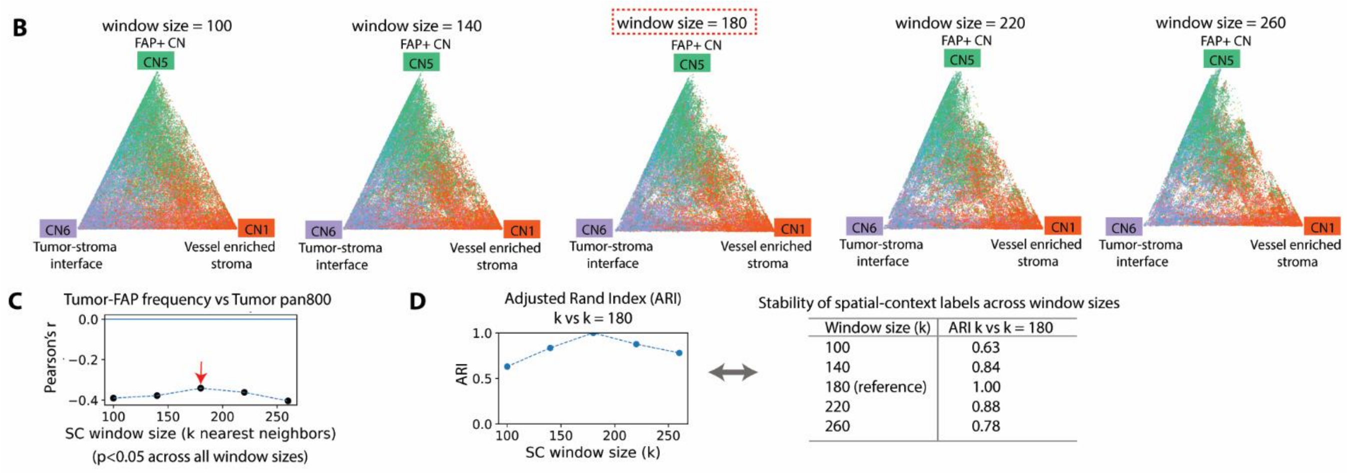

Supplementary Figure 9. Nanostring GeoMx DSP data analysis in HNSCC patients.

(A) Method overview for spatial transcriptomics using Nanostring GeoMx DSP.

(B-C) Gene set enrichment analysis (GSEA) pathway enrichment plots for pathways associated with B) degradation of extracellular matrix in FAP+ cells and C) EGFR downregulation in PanCK+ cells in patients with high tumor concentrations of pan800 (N = 6 patients).

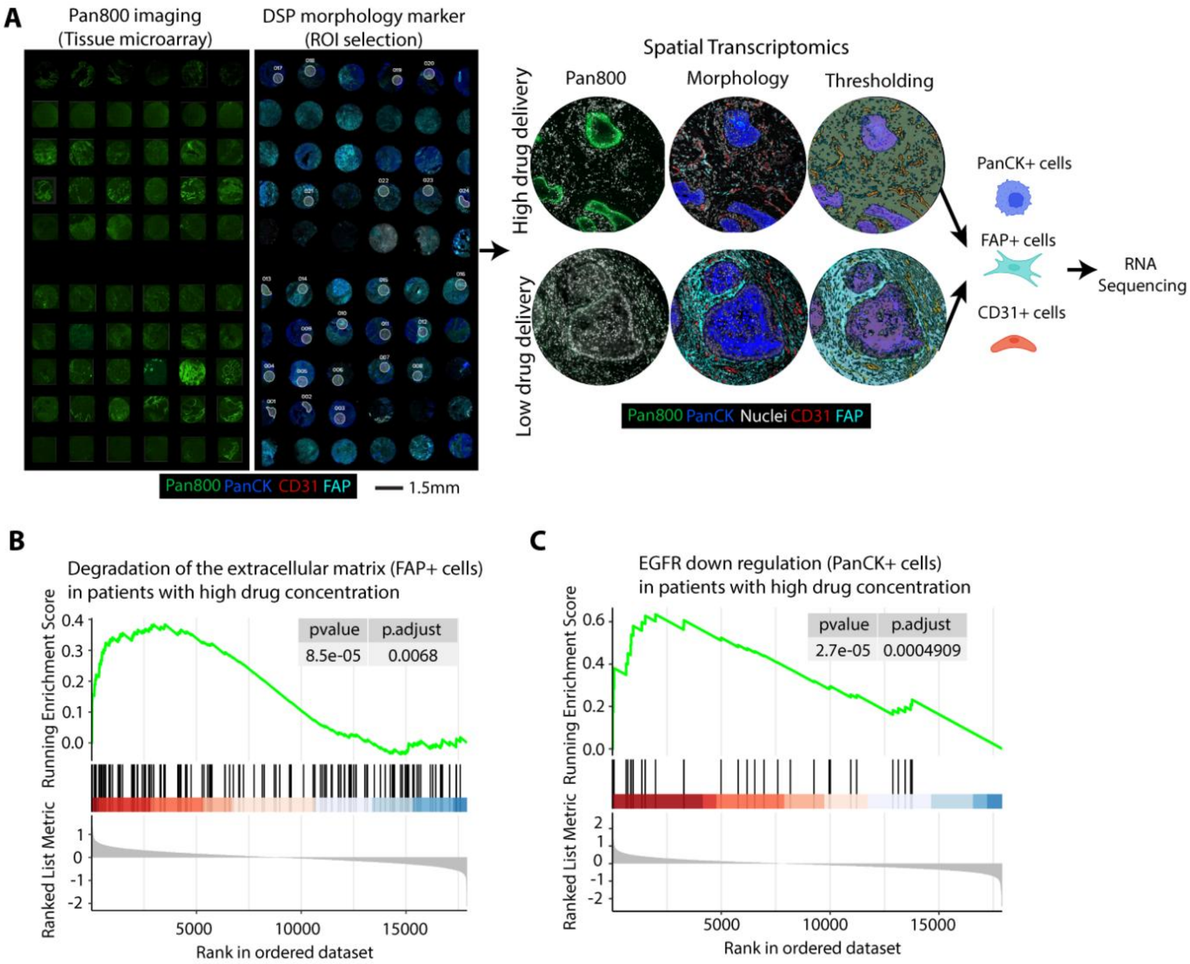

Supplementary Figure 10. Data analysis in PDAC patients.

(A) The CONSORT diagram of the PDAC clinical study.

(B) Dot plot of protein marker expression for each cell type at a high granularity for PDAC tissues (N = 12 patients).

(C) Dot plot of protein markers for fibroblasts in PDAC tissues (N = 12 patients).

(D) Composition of fibroblast subtypes in PDAC tissues (N = 12 patients).

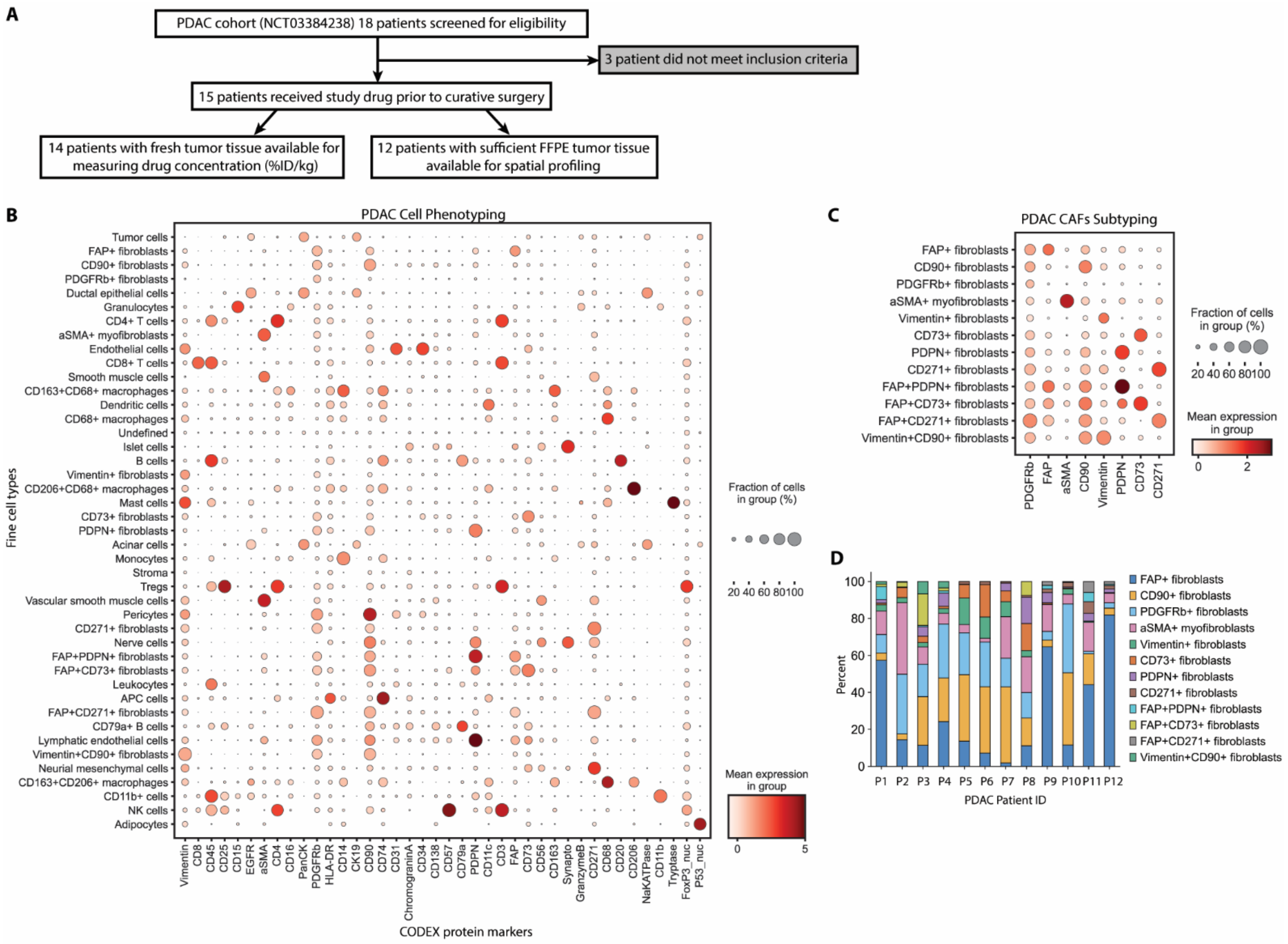

Supplementary Figure 11. Sensitivity analysis of window size in PDAC patients.

(A) CN analysis across window sizes ranging from 5 to 25 nearest cells in PDAC. Red dotted line outlined vascular cells, including endothelial cells, pericytes, and vascular smooth muscle cells. Black dotted line outlined tumor cells. Orange dotted line outlined FAP+ fibroblasts. A window size of 10 nearest cells yielded optimal biological separation of tumor-enriched, vessel-associated, and FAP^+ fibroblast neighborhoods, reflecting the distinct cellular density and stromal organization of PDAC relative to HNSCC.

(B) Barycentric spatial-context maps generated using k-nearest-neighbor windows with k = 100, 140, 180, 220, and 260 in PDAC tissues. Each point represents a local window projected into barycentric space based on the relative composition of three cellular neighborhoods.

(C) Association between the frequency of the Tumor–FAP^+ stroma interface and mean tumor pan800 across window sizes in PDAC. Pearson correlation coefficients demonstrate a directionally consistent inverse association at intermediate window sizes (nominal two-sided p values).

(D) Agreement of SC label assignments across window sizes, quantified by the adjusted Rand index (ARI) relative to the reference window size k = 180. SC labels exhibit maximal agreement at intermediate window sizes (k = 140–220), with lower agreement at smaller and larger windows.

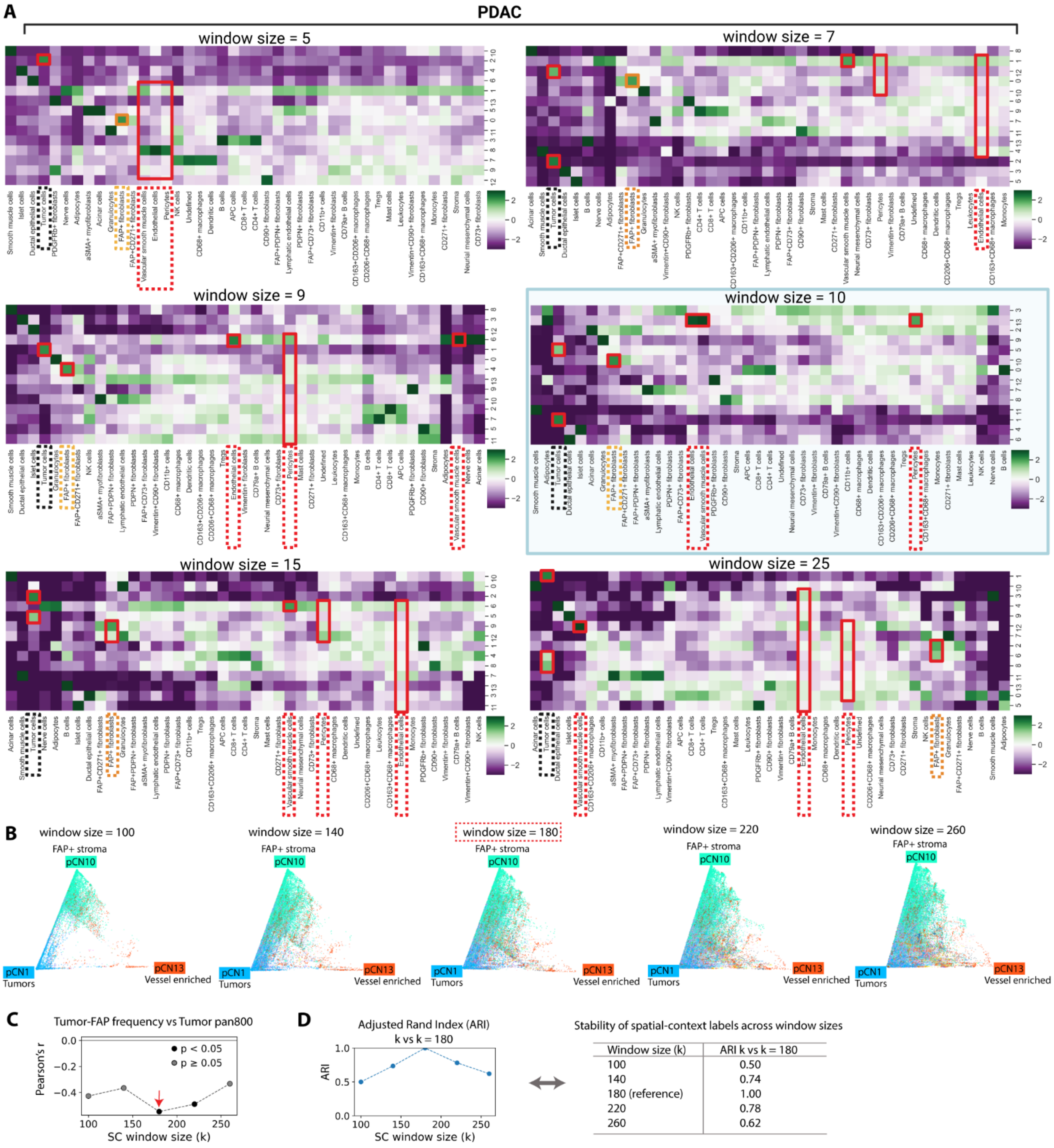

Window size (k)	ARI k vs k = 180
100	0.50
140	0.74
180 (reference)	1.00
220	0.78
260	0.62

Supplementary Figure 12. Patient-level robustness of region-based correlations for Figures 3J, 4J, and 4K.

(A, C, E) Region-level scatter plots corresponding to Figures 3J, 4J, and 4K, respectively. Each dot represents one tissue region (TMA core) and is colored by patient identity, allowing visual assessment of patient-level contributions to the observed correlations.

(B, D, F) Leave-one-patient-out analyses for the correlations shown in Figures 3J, 4J, and 4K. Each dot represents the Pearson correlation coefficient recomputed after excluding one patient at a time. Dot color indicates the statistical significance of the correlation based on the associated p value. The horizontal dashed line denotes the mean correlation coefficient across all LOO runs.

For Figures 3J and 4J, exclusion of patients 1 or 17 attenuated correlation strength while preserving effect direction; statistical significance was reduced in the most affected exclusions (from $p < 0.01$ to $p < 0.05$).

For Figure 4K, exclusion of patient 17 substantially attenuated the correlation and reduced it below statistical significance, indicating greater patient-to-patient heterogeneity for this stromal-coupling relationship.

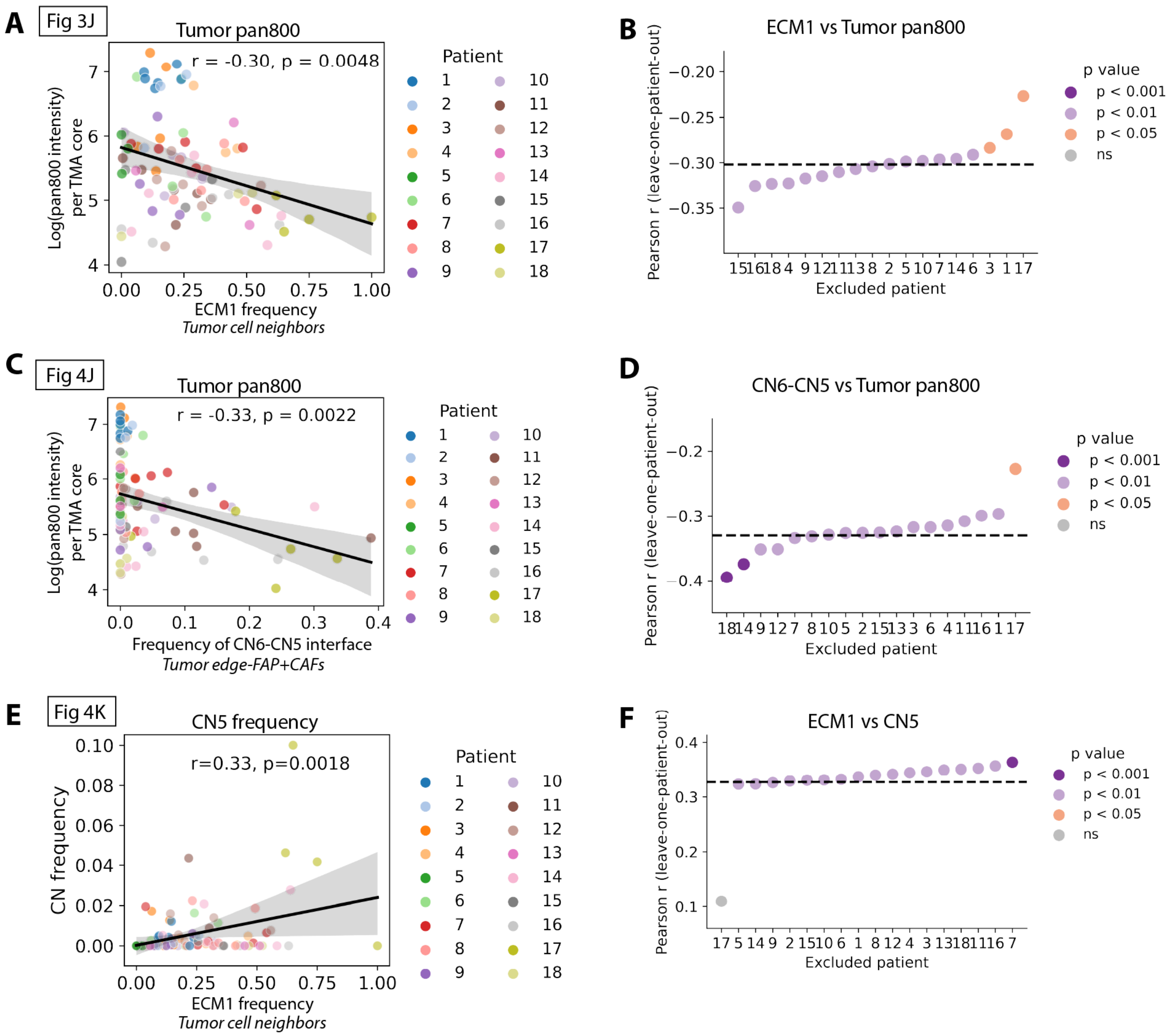

Supplementary Figure 13. Patient-level robustness of region-based correlations for Figure 3H, 4G, and 4H.

(A, C, E) Region-level correlation corresponding to Figure 3H, Figure 4G, and Figure 4H, respectively. Each dot represents one tissue region (TMA core) and is colored by patient identity, allowing visual assessment of patient-level contributions to the observed correlations.

(B, D, F) Leave-one-patient-out analysis for the correlation shown in Figure 3H, Figure 4G, and Figure 4H. Each dot represents the Pearson correlation coefficient recomputed after excluding one patient at a time. Dot color indicates the statistical significance of the correlation based on the associated p value. The horizontal dashed line denotes the mean correlation coefficient across all LOO runs.

For Figure 3H, the negative direction of the association and statistical significance were preserved across all exclusions.

For Figure 4G, leave-one-patient-out analyses demonstrated that the negative direction of the association was preserved across all patient exclusions. The correlation remained statistically significant for the majority of exclusions (15 of 18), with a small number of exclusions yielding reduced significance due to decreased variance.

For Figure 4H, the positive direction of the association and statistical significance was preserved across all exclusions.

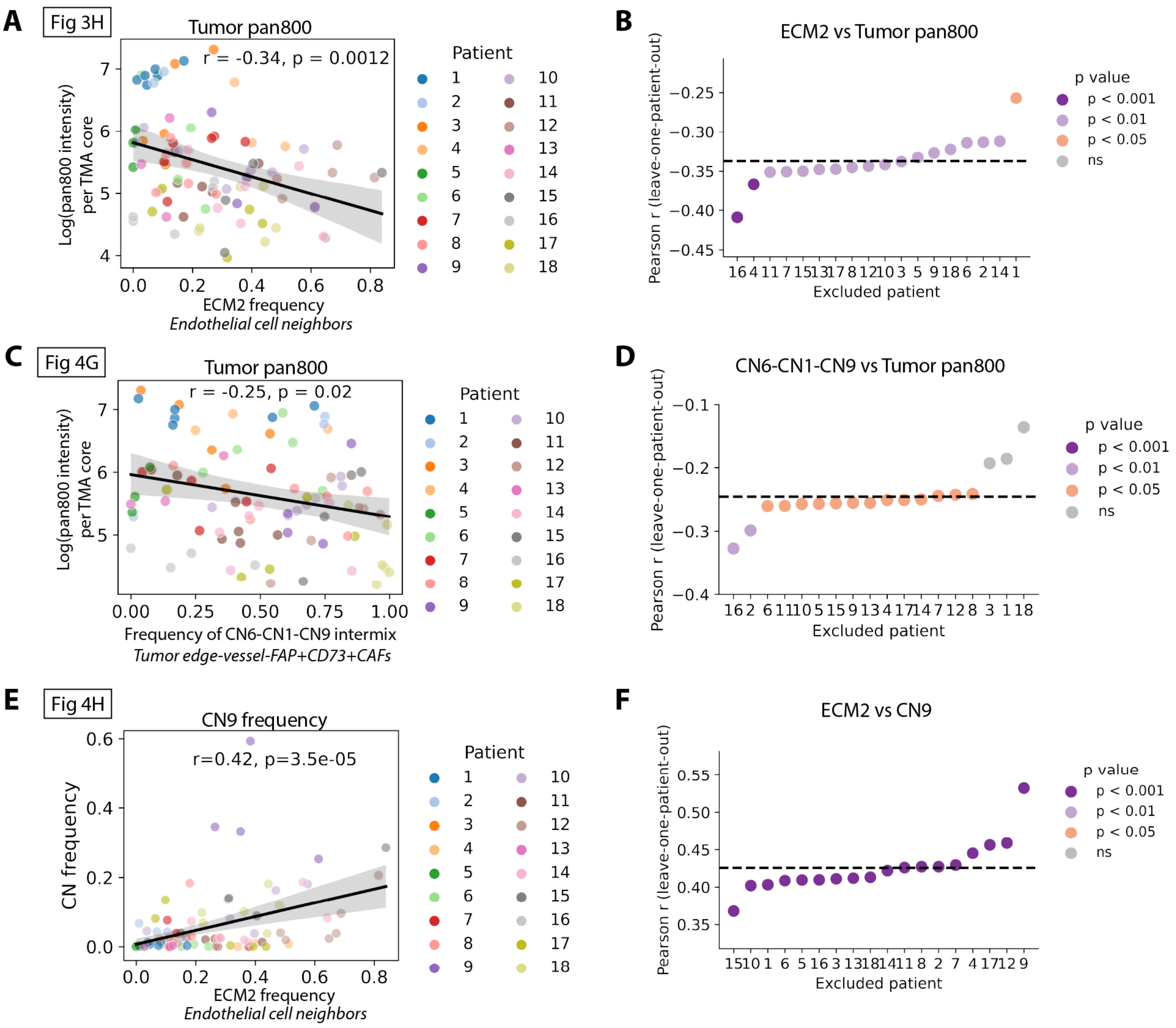
A
Fig 3H
Tumor pan800
r = -0.34, p = 0.0012
Log(pan800 intensity) per TMA core
ECM2 frequency
Endothelial cell neighbors
Patient
B
ECM2 vs Tumor pan800
Pearson r (leave-one-patient-out)
Excluded patient
p value
p < 0.001
p < 0.01
p < 0.05
ns
C
Fig 4G
Tumor pan800
r = -0.25, p = 0.02
Log(pan800 intensity) per TMA core
Frequency of CN6-CN1-CN9 intermix
Tumor edge-vessel-FAP+CD73+CAFs
Patient
D
CN6-CN1-CN9 vs Tumor pan800
Pearson r (leave-one-patient-out)
Excluded patient
p value
p < 0.001
p < 0.01
p < 0.05
ns
E
Fig 4H
CN9 frequency
r=0.42, p=3.5e-05
CN frequency
ECM2 frequency
Endothelial cell neighbors
Patient
F
ECM2 vs CN9
Pearson r (leave-one-patient-out)
Excluded patient
p value
p < 0.001
p < 0.01
p < 0.05
ns

Supplementary Figure 14. Patient-level assessment of region-based correlations in Figures 5G, 5O, and 5P.

(A, C, E) Region-level correlation corresponding to Figure 5G, Figure 5O, and Figure 5P, respectively. Each dot represents one tissue region (TMA core) and is colored by patient identity, allowing visual assessment of patient-level contributions to the observed correlations.

(B, D, F) Leave-one-patient-out analysis for the correlation shown in Figure 5G, Figure 5O, and Figure 5P. Each dot represents the Pearson correlation coefficient recomputed after excluding one patient at a time. Dot color indicates the statistical significance of the correlation based on the associated p value. The horizontal dashed line denotes the mean correlation coefficient across all LOO runs.

For Figure 5G, the negative direction of the association was preserved across all exclusions. The correlation remained statistically significant for two of four exclusions (patients 1 and 2), with reduced statistical significance upon exclusion of patients 3 and 4, reflecting decreased variance rather than reversal of the association.

For Figure 5O, the negative direction of the association was preserved across all exclusions. The correlation remained statistically significant upon exclusion of patients 2, 3, and 4, while exclusion of patient 1 reduced the correlation strength and resulted in loss of statistical significance.

For Figure 5P, the positive direction of the association was preserved across all exclusions. The correlation remained statistically significant for three of four exclusions (patients 1–3), with reduced correlation strength and loss of statistical significance upon exclusion of patient 4, indicating that this patient contributes substantial variation to the overall association.

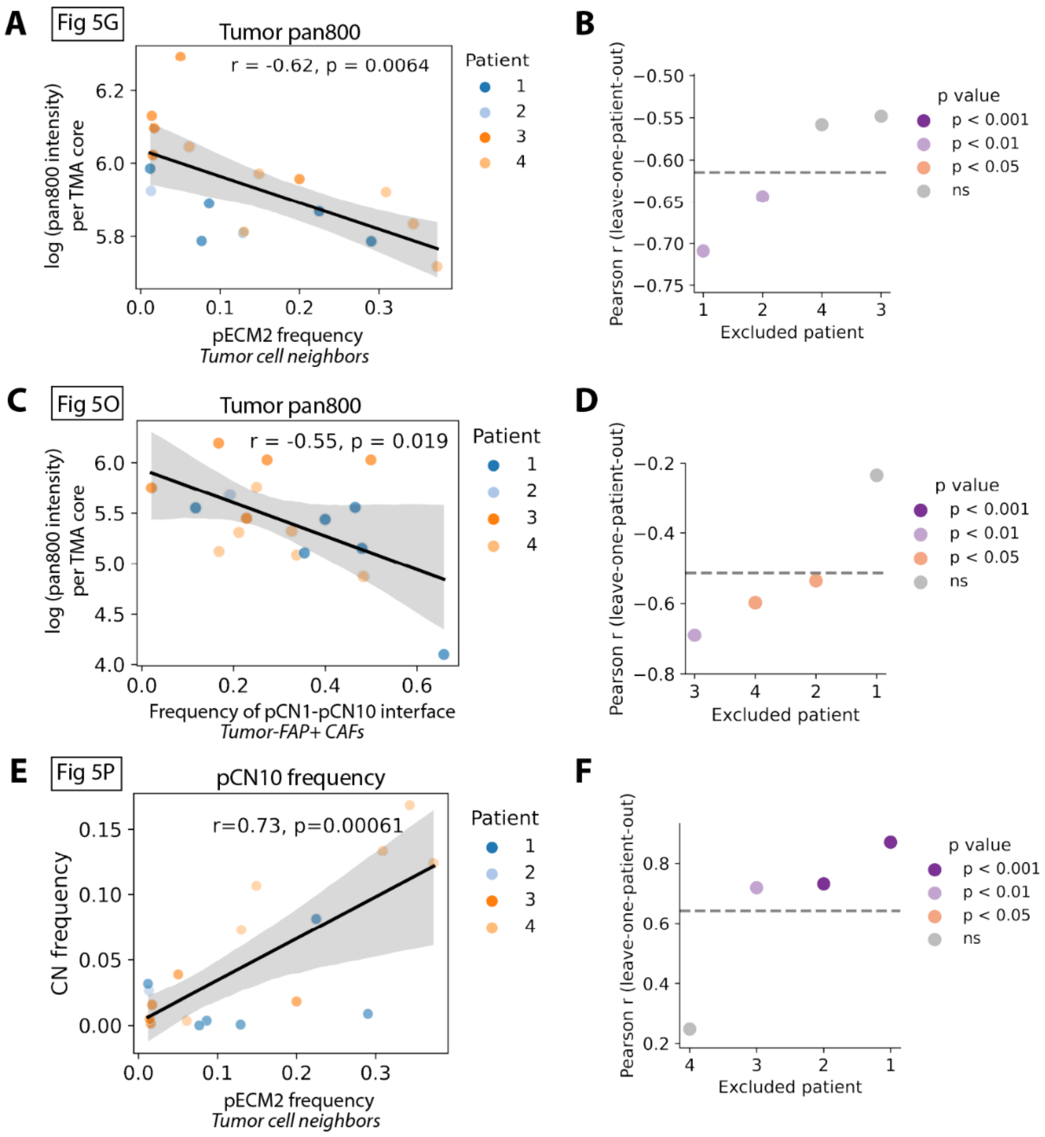
A
Fig 5G
Tumor pan800
r = -0.62, p = 0.0064
log (pan800 intensity) per TMA core
pECM2 frequency
Tumor cell neighbors
Patient
1
2
3
4
B
Pearson r (leave-one-patient-out)
Excluded patient
p value
p < 0.001
p < 0.01
p < 0.05
ns
C
Fig 5O
Tumor pan800
r = -0.55, p = 0.019
log (pan800 intensity) per TMA core
Frequency of pCN1-pCN10 interface
Tumor-FAP+ CAFs
Patient
D
Pearson r (leave-one-patient-out)
Excluded patient
p value
E
Fig 5P
pCN10 frequency
r=0.73, p=0.00061
CN frequency
pECM2 frequency
Tumor cell neighbors
Patient
F
Pearson r (leave-one-patient-out)
Excluded patient
p value

Supplementary Figure 15. Single-cell spatial transcriptomics (Xenium) independently validates associations between tumor drug delivery and tumor-proximal FAP$^+$ CAF niches.

(A) Xenium spatial transcriptomic profiling of representative HNSCC and PDAC tissue microarray (TMA) cores analyzed following same-slide pan800 imaging.

(B) Expression of FAP and POSTN genes (encoding periostin) across major cell types in HNSCC (n = 10 TMA cores from 3 patients) and PDAC tissues (n = 14 TMA cores from 3 patients).

(C-D) Scatter plots showing the relationship between log-transformed tumor pan800 intensity and (C) log-transformed POSTN expression in tumor-proximal FAP$^+$ CAFs and (D) the ratio of tumor-proximal FAP$^+$ CAFs to tumor cells in HNSCC tissues.

(E-F) Scatter plots showing the relationship between log-transformed tumor pan800 intensity and (E) log-transformed POSTN expression in tumor-proximal FAP$^+$ CAFs and (F) the ratio of tumor-proximal FAP$^+$ CAFs to tumor cells in PDAC tissues (n = 14 TMA cores from 3 patients).

(G-H) Representative examples of regions with relatively high pan800 delivery (G) versus low pan800 delivery (H), and corresponding tumor–FAP$^+$ CAF spatial maps, illustrating that lower pan800 delivery is associated with higher tumor-proximal FAP$^+$ CAF abundance and higher POSTN expression.

For all scatter plots (C-F), associations were assessed using Pearson's correlation, with two-tailed p values adjusted for multiple comparisons using the Benjamini–Hochberg method. Shaded area indicates 95 confidence interval of regression curve.

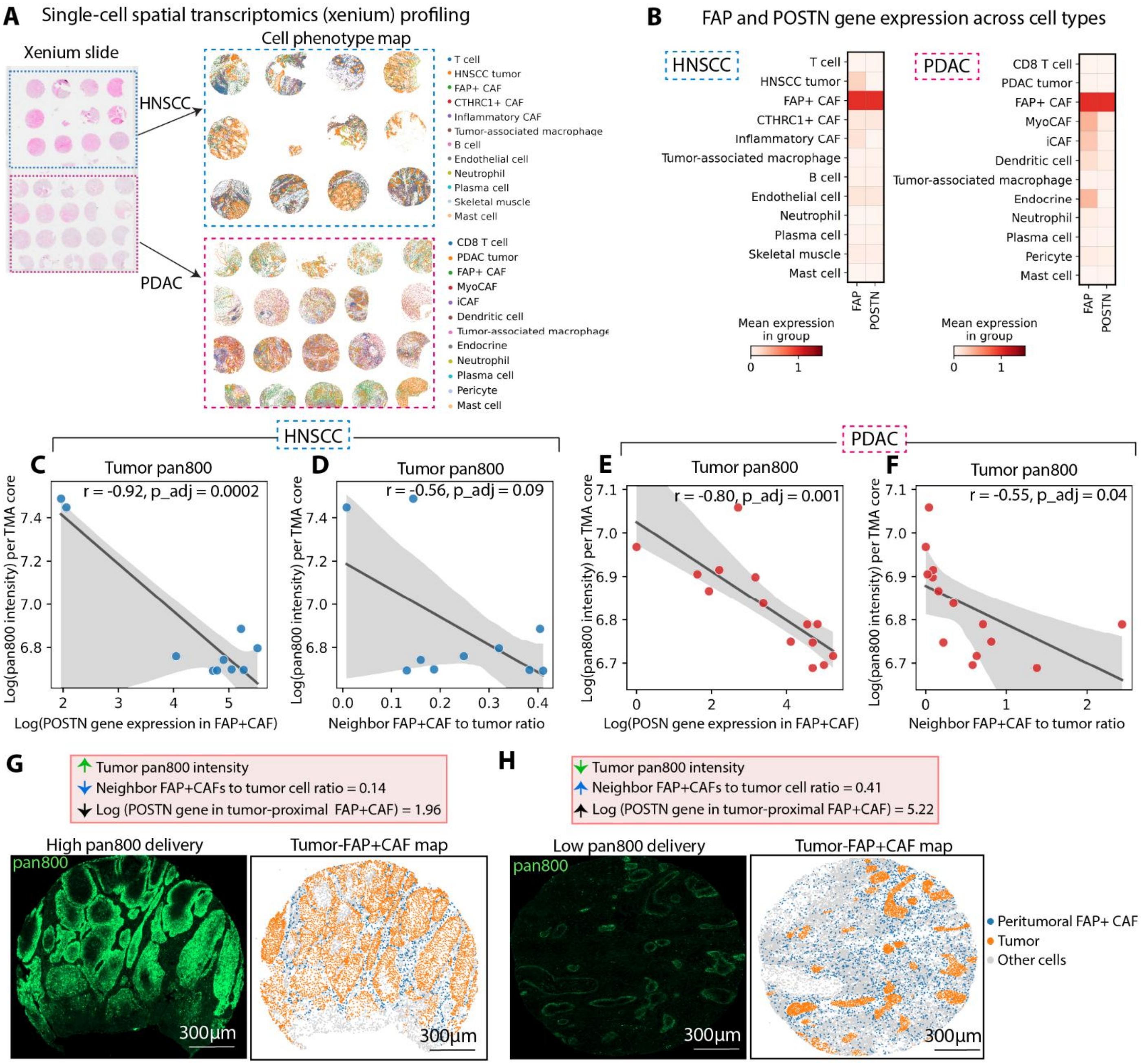

A Single-cell spatial transcriptomics (xenium) profiling
Xenium slide
Cell phenotype map
HNSCC
PDAC
T cell
HNSCC tumor
FAP+ CAF
CTHRC1+ CAF
Inflammatory CAF
Tumor-associated macrophage
B cell
Endothelial cell
Neutrophil
Plasma cell
Skeletal muscle
Mast cell
CD8 T cell
PDAC tumor
MyoCAF
iCAF
Dendritic cell
Endocrine
Pericyte
B FAP and POSTN gene expression across cell types
FAP
POSTN
Mean expression in group
C Tumor pan800 r = -0.92, p_adj = 0.0002
Log(POSTN gene expression in FAP+CAF)
D Tumor pan800 r = -0.56, p_adj = 0.09
Neighbor FAP+CAF to tumor ratio
E Tumor pan800 r = -0.80, p_adj = 0.001
Log(POSN gene expression in FAP+CAF)
F Tumor pan800 r = -0.55, p_adj = 0.04
Log(pan800 intensity) per TMA core
G
Tumor pan800 intensity
Neighbor FAP+CAFs to tumor cell ratio = 0.14
Log (POSTN gene in tumor-proximal FAP+CAF) = 1.96
High pan800 delivery
Tumor-FAP+CAF map
H
Tumor pan800 intensity
Neighbor FAP+CAFs to tumor cell ratio = 0.41
Log (POSTN gene in tumor-proximal FAP+CAF) = 5.22
Low pan800 delivery
pan800
300µm
Peritumoral FAP+ CAF
Tumor
Other cells

Supplementary Figure 16. Comparison of pan800 drug distribution and endogenous human IgG localization in a representative HNSCC tumor.

(A) Near-infrared fluorescence imaging of panitumumab-IRDye800 (pan800) in a representative tumor tissue from an HNSCC patient systemically infused with pan800, showing heterogeneous intratumoral drug distribution with reduced signal within tumor nest interiors.

(B) Immunohistochemical staining for anti-human Fc IgG on the same tissue section, visualizing the distribution of endogenous human IgG as a proxy for generic macromolecule delivery. Endogenous IgG exhibits a more diffuse intratumoral distribution and is detectable within tumor nests where pan800 signal is reduced (representative regions indicated by paired arrows in red, cyan, and magenta).

The presence of endogenous IgG within tumor nests supports interpretation that reduced pan800 accumulation reflects target-mediated binding-site barrier effects rather than complete physical exclusion of macromolecules, while remaining consistent with stroma-associated transport limitations.

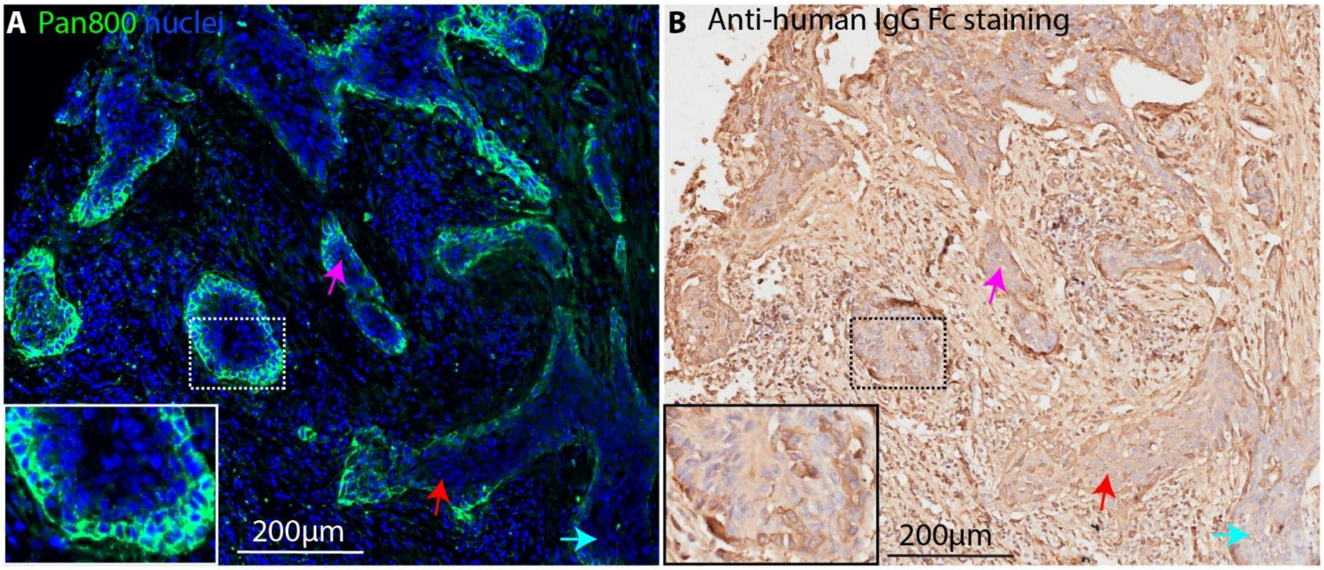

References

1. Gatenbee CD, Baker AM, Prabhakaran S, Swinyard O, Slebos RJC, Mandal G, Mulholland E, Andor N, Marusyk A, Leedham S, et al. Virtual alignment of pathology image series for multi-gigapixel whole slide images. *Nat Commun*. 2023;14:4502. doi: 10.1038/s41467-023-40218-9
2. Stringer C, Wang T, Michaelos M, Pachitariu M. Cellpose: a generalist algorithm for cellular segmentation. *Nat Methods*. 2021;18:100-106. doi: 10.1038/s41592-020-01018-x
